# Copper-Catalyzed Synthesis of Masked (Hetero)Aryl Sulfinates

**DOI:** 10.1021/acs.orglett.3c03621

**Published:** 2024-01-08

**Authors:** May R. Merino, Xinlan A. F. Cook, David C. Blakemore, Ian B. Moses, Neal W. Sach, Andre Shavnya, Michael C. Willis

**Affiliations:** † Department of Chemistry, University of Oxford, Chemistry Research Laboratory, Mansfield Road, Oxford OX1 3TA, U.K.; ‡ Medicine Design, Pfizer Inc., Eastern Point Road, Groton, Connecticut 06340, United States; § Pharmaceutical Sciences, Pfizer Inc., Discovery Park, Ramsgate Road, Kent CT13 9ND, U.K.; ∥ Medicine Design, La Jolla Laboratories, Pfizer Inc., 10777 Science Center Drive, San Diego, California 92121, United States

## Abstract

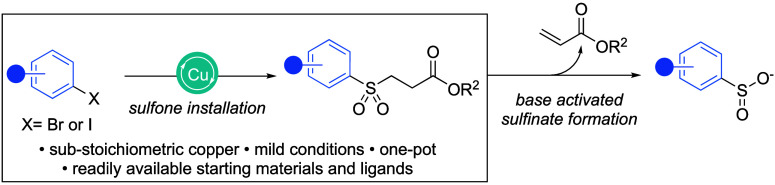

Catalysis using substoichiometric copper facilitates the synthesis of masked (hetero)aryl sulfinates under mild, base-free conditions from aryl iodides and the commercial sulfonylation reagent sodium 1-methyl 3-sulfinopropanoate (SMOPS). The development of a *tert*-butyl ester variant of the SMOPS reagent allowed the use of aryl bromide substrates. The sulfones thus generated can be unmasked and functionalized in situ to form a variety of sulfonyl-containing functional groups.

Aryl sulfinates^1,2^ are key precursors for the synthesis of medicinally relevant sulfonyl derivatives, including sulfones, sulfonamides, and sulfonyl halides.^3^ Sulfinates are also versatile synthetic reagents, with the potential to react as either “electrophilic”^4−6^ or “nucleophilic”^7−12^ components in cross-coupling reactions. Classical syntheses of sulfinates often require harsh reaction conditions and toxic reagents; methods include the reduction of sulfonyl chlorides using zinc, oxidation of thiols with hydrogen peroxide, or the insertion of toxic SO_2_ gas into organometallics.^2^ More recently, SO_2_ surrogates^13^ such as DABSO,^14^ sodium dithionite,^15^ and metabisulfites^16−18^ have been used in conjunction with aryl halides in the synthesis of aryl sulfinates. Although many of these methods have enjoyed success, the intrinsic ionic nature of sulfinate salts can present practical challenges during isolation and purification due to their insolubility in organic media and their hygroscopicity.^2,19^ For example, the removal of water is often required during the isolation of sulfinates, which is impractical and can lead to downstream issues when ensuring the complete dryness of these products. As a consequence, sulfinates are often reacted and functionalized in situ, and their direct isolation remains a challenge.

An effective strategy to avoid the isolation of sulfinate salts is to use molecules that behave as “masked sulfinates”, i.e., molecules that release a sulfinate functional group under specific reaction conditions. Benzothiazolesulfinate,^20^ rongalite,^21,22^ and closely related rongacyl^23^ have been used primarily in the synthesis of aliphatic masked sulfinates. More recently the TBS-protected derivative of rongalite, TBSOMS-Na (**1**), has been applied as a nucleophilic coupling partner in both alkylation and copper-catalyzed arylation reactions, delivering aryl variants (Scheme 1a).^24^ The TBSOMS-Na chemistry, although efficient, faces drawbacks, particularly in large-scale applications, as the reaction requires either expensive iodonium triflate salts or a noncommercial ligand. The sulfinate reagent itself is also noncommercial. The use of a fluoride source can also lead to functional group compatibility issues. In the context of a sulfonamide synthesis, Baskin and Wang reported the use of β-ester sulfones as masked sulfinates,^25^ with the sulfinates being liberated under basic conditions, and this chemistry has now been widely applied in industrial laboratories.^26−29^ In the original report, the sulfones were prepared from the combination of sodium-3-methoxy-3-oxopropane-1-sulfinate **2** (SMOPS) with alkyl and aryl halides. For aryl substrates, a copper-mediated coupling procedure was used, and although effective, the chemistry requires a large excess of both SMOPS and copper iodide (usually 3 equiv), as well as a high temperature of 110 °C, and yields from aryl bromides are only modest (Scheme 1b). The SMOPS reagent is now commercially available.

**Scheme 1:**
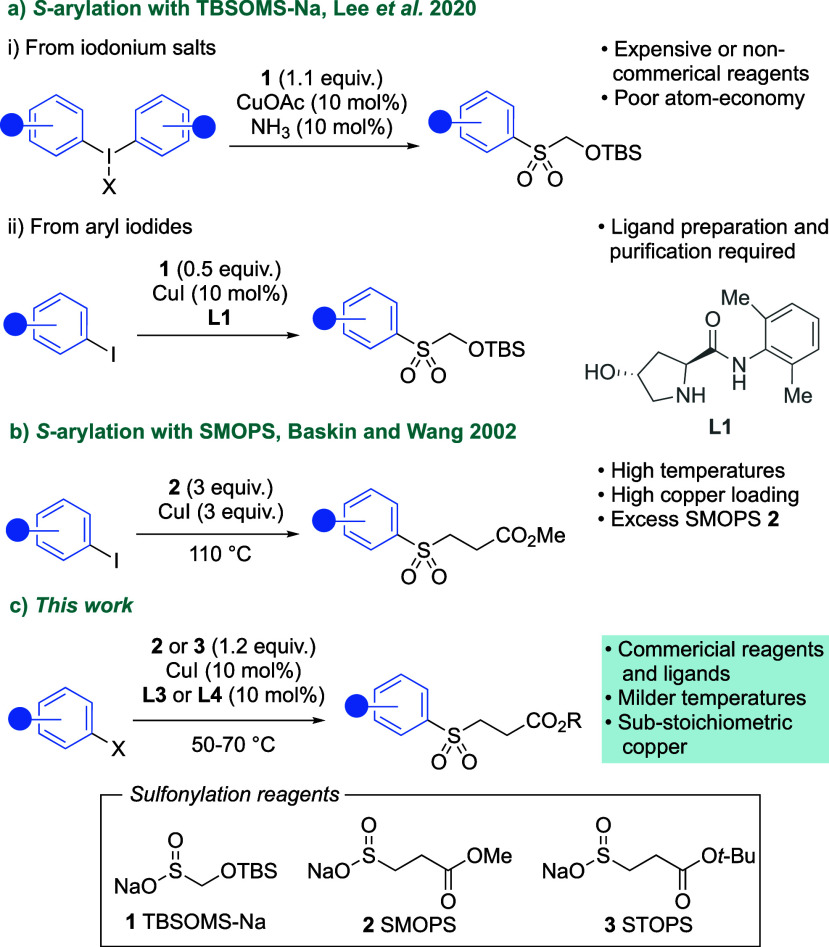
(a) TBSOMS-Na as a sulfonylation agent. (b) SMOPS as a sulfonylation agent. (c) This work: copper-catalyzed synthesis of aryl sulfones.

Our interest in aryl sulfinates, and in particular 2-pyridyl sulfinates, stems from the long-term collaboration between the University of Oxford and Pfizer’s research and development organization, aimed at identifying solutions to the synthesis of aryl-linked heteroaromatics. This partnership has established that 2-pyridyl sulfinates, and related masked variants, are versatile reaction partners in palladium-catalyzed desulfinative coupling reactions.^19^ 2-Pyridyl β-ester sulfones performed well as “masked sulfinates”,^19d^ and in our prior work the required sulfones were prepared using a two-step synthesis starting from pyridine thiols. In the current report, we describe the development of an improved one-step procedure for the preparation of (hetero)aryl β-ester sulfones and in particular 2-pyridyl variants. The developed chemistry involves the copper-catalyzed coupling of (hetero)aryl halides and the SMOPS reagent, but importantly it uses substoichiometric amounts of copper salt, typically 10 mol %, along with only 1.2 equiv of the SMOPS reagent and is conducted at moderate reaction temperatures (Scheme 1c).

In our initial investigations (Scheme 2), we focused on the coupling of 2-iodopyridine **4a** and SMOPS reagent (**2**). We explored the use of 6-hydroxypicolinamide ligands^30^ with conditions adapted from Lee.^24^ Evaluation of a small selection of these ligands gave a maximum yield of 50% with ligand **L2**. After this encouraging result, a high throughput screen was performed (see Supporting Information), which uncovered that commercial Ullmann ligands were most effective, with the cheap and widely available ligand **L3** outperforming other ligands, including oxalamides such as **L4**. We found that the removal of base was beneficial (entry 4) and that the reaction worked well with aryl iodide as the limiting reagent and SMOPS in slight excess (1.2 equiv). Finally, increasing the reaction temperature from 35 to 50 °C gave the optimized conditions (entry 5).

**Scheme 2:**
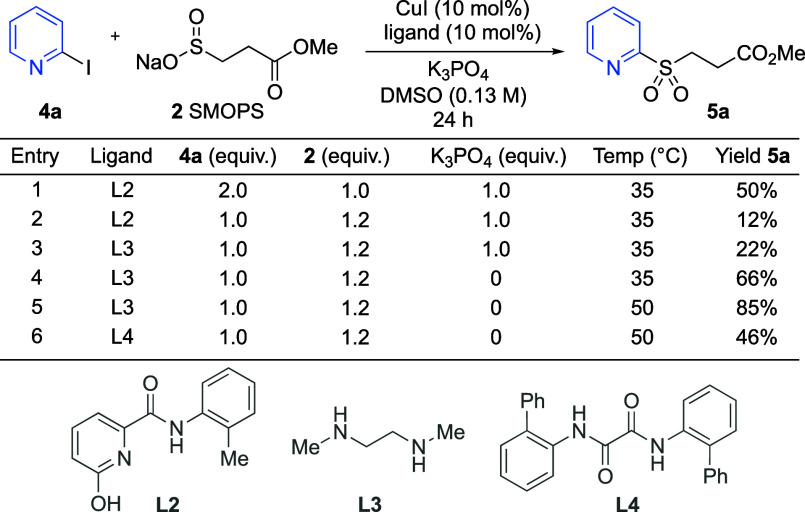
Selected Optimization Studies

We next explored the scope of the process with respect to the (hetero)aryl iodide component (Scheme 3a). In general, pyridyl substrates featuring a variety of substituents worked well, although substrates featuring electron-donating substituents delivered the most efficient reactions (**5a**–**5i**). It was encouraging that 2,3-disubstituted pyridines (**5c**) could be prepared, as this arrangement was challenging for earlier methods.^24,31^ Pyrazine, pyrimidine, and quinoline examples were also successful (**5j**–**5l**). Notably, 2-sulfonylpyridines and pyrimidines have recently been used as covalent inhibitors.^32,33^ Electronically varied benzenes were also competent substrates (**5m**–**p**).

**Scheme 3:**
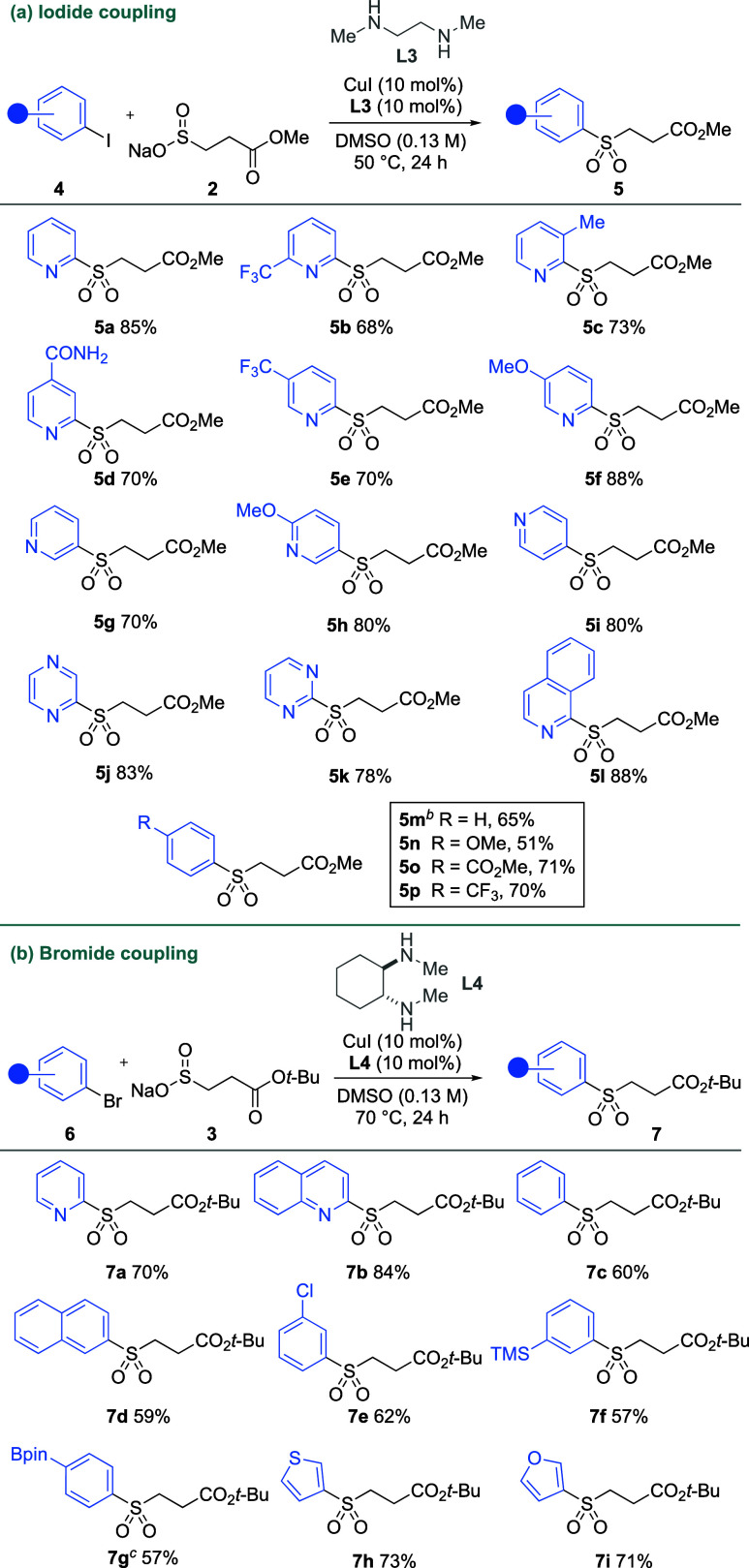
(a) (Hetero)Aryl Iodide and (b) (Hetero)Aryl Bromide Coupling Scope (a) Iodide coupling reaction conditions: **4** (0.2 mmol, 1.0 equiv), SMOPS (**2**) (0.24 mmol, 1.2 equiv), CuI (10 mol %), **L3** (10 mol %), DMSO (0.13 M), 50 °C, 24 h. Reaction performed on 0.5 mmol scale. (b) Bromide coupling reaction conditions: **6** (0.2 mmol, 1.0 equiv), STOPS (**3**) (0.24 mmol, 1.2 equiv), CuI (10 mol %), **L4** (10 mol %), DMSO (0.13 M), 70 °C, 24 h. 20 mol % CuI used, the corresponding alcohol was isolated after oxidation (7g*, see the Supporting Information).

Following the successful coupling of (hetero)aryl iodides with SMOPS, our focus was turned to aryl bromide substrates. The use of aryl bromides is attractive due to their generally lower cost and greater structural diversity. Using the optimized conditions from the iodide investigation on 2-bromoquinoline **6b**, with an increased temperature of 70 °C, provided sulfone **7b** in 48% yield. From trialing the same ligands as before, we found that cyclic Ullmann ligand **L4** worked best. After a further round of optimization (see Supporting Information), we found that the increased temperature caused hydrolysis of the methyl ester portion of the product, leading to a significant reduction in yield. This was confirmed by LCMS analysis and stability tests of the product under the reaction conditions, where up to 40% of the product was depleted after 24 h. In addition, increasing the temperature to 90 °C, or extending the reaction time to 48 h, gave further reduced yields. To counteract this, we proposed replacing the methyl ester in the sulfonylation reagent with a *tert*-butyl ester. The new reagent, STOPS (**3**), was readily accessed in three steps on a large scale without need for purification by flash chromatography. Employing this new reagent improved the yields for aryl bromide couplings; for example, the yield of sulfone **7b** increased from 66% to 84% (Scheme 3b). Using this modified protocol, we explored the aryl bromide scope (Scheme 3b) and found that a variety of substituents were well tolerated; thus, products including chloride (**7e**), silyl (**7f**), and boronic ester (**7g**), were successfully formed. The boronic ester product **7g** was converted to the corresponding phenol 7g* for isolation. Products derived from electron-rich heterocycles such as thiophene **7h** and furan **7i** were both isolated in good yields.

Aryl chloride substrates were briefly explored, but only low yields of sulfones could be achieved.

To demonstrate the utility of the products as sulfinate precursors, we explored the functionalization of methyl ester and *tert*-butyl ester sulfones **5a** and **7a** (Scheme 4). Simply stirring the sulfones with sodium methoxide (1.0 equiv) generated the corresponding sulfinates within 15 min at room temperature, after which the addition of an electrophile forms the desired sulfonyl derivative in a good yield. The base lability of the methyl ester sulfones has been explored previously,^19^ and we were pleased to observe that the *tert*-butyl ester sulfones worked analogously. Reactivity was exemplified in the formation of sulfone **8a** by the addition of *tert*-butyl bromoacetate as the electrophile. The sulfonyl fluoride PyFluor (**8b**), a deoxyfluorination agent, was prepared by the addition of NFSI to the in situ generated sulfinates.^34^ We also showcased the formation of sulfonamides by the addition of NCS and morpholine to the sulfinates, which provided sulfonamide **8c** in good yields.

**Scheme 4:**
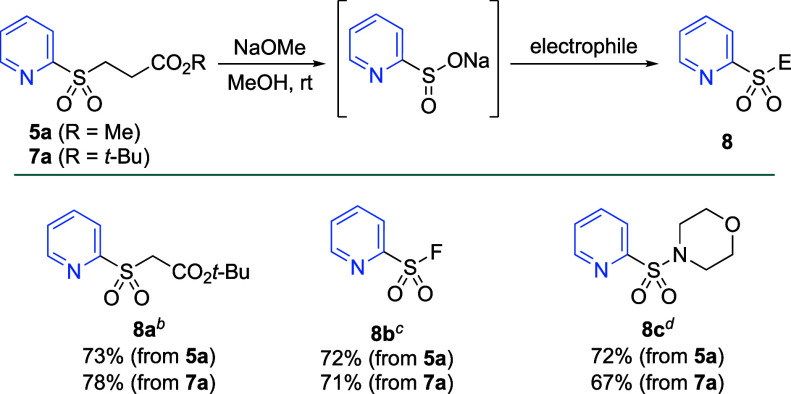
Functionalization of Masked (Hetero)Aryl Sulfinates Reaction conditions: **5a** or **7a** (0.2 mmol, 1.0 equiv), NaOMe (30% w/w in MeOH, 0.2 mmol, 1.0 equiv), DMSO (0.1 M), 15 min, rt. General reaction conditions, then *tert*-butyl bromoacetate (0.4 mmol, 2.0 equiv). General reaction conditions, then NFSI (0.3 mmol, 1.5 equiv). General reaction conditions, then NCS (0.4 mmol, 2.0 equiv) and morpholine (0.4 mmol, 2.0 equiv).

In summary, we have developed a copper-catalyzed method for the preparation of β-ester (hetero)aryl sulfones, which serve as effective masked sulfinate reagents. The reaction is base-free and uses mild conditions and commercially available starting materials when using (hetero)aryl iodide substrates. For aryl bromide substrates, a modified, readily accessible sulfinate reagent STOPS is used, which is prepared in three steps. The sulfones formed can be unmasked to form sulfinates under basic conditions, which can be functionalized to form a range of sulfonyl functional groups.

## Data Availability

The data underlying this study are available in the published article and its Supporting Information.

## Supporting Information Available

Experimental procedures and supporting characterization data and spectra (PDF)
