# Supplementary material for: Copper-Catalyzed Synthesis of Masked (Hetero)Aryl Sulfinates
Source: Org Lett. 2024 Jan 8;26(14):2817–20. doi: 10.1021/acs.orglett.3c03621 (PMC11020165; doi:10.1021/acs.orglett.3c03621)
Supplement: Supplementary file 1 — ol3c03621_si_001.pdf [file ol3c03621_si_001.pdf]

Supporting Information

Copper-Catalyzed Synthesis of Masked (Hetero)Aryl Sulfinates

May R. Merino,[a] Xinlan A. F. Cook,[a] David C. Blakemore,[b] Ian B. Moses,[c] Neal W. Sach,[d] Andre Shavnya[b] and Michael C. Willis[a*]

[*] michael.willis@chem.ox.ac.uk

[a] Department of Chemistry, University of Oxford, Chemistry Research Laboratory, Mansfield Road, Oxford, OX1 3TA, UK

[b] Medicine Design, Pfizer Inc., Eastern Point Road, Groton, CT, 06340, USA

[c] Pharmaceutical Sciences, Pfizer Inc., Discovery Park, Ramsgate Road, CT13 9ND, UK

[d] Medicine Design, La Jolla Laboratories, Pfizer Inc., 10777 Science Center Drive, San Diego, CA, 92121, USA

Table of Contents

1. General considerations 3
2. Optimisation of copper-catalyzed reactions 4
   2.1. Optimisation of coupling from iodides 4
   2.2. Optimisation of coupling from bromides 5
3. Coupling from aryl chloride 7
4. Copper-catalyzed coupling conditions high throughput screen 8
   4.1 Screening parameters 8
   4.2 Experimental procedure 8
   4.3 Results from screen 9
5. Experimental procedures 11
   5.1. Synthesis of sulfonylation reagents 11
   5.2. Substrate Scopes 13
   5.3. Functionalisation of masked sulfinates 23
6. NMR Spectra 26
7. References 60

1. General considerations

All reactions were performed under an inert atmosphere with constant magnetic stirring, unless otherwise stated, using clean, oven-dried glassware. All inert gases were sourced from the University of Oxford's internal supplies and dried through $CaCl_2$ drying columns. Reactions were monitored by thin-layer chromatography (TLC) which was performed on Merck Kieselgel 60 PF254 pre-coated aluminium backed TLC sheets and visualized under UV light (λ = 254 nm and 365 nm) and/ or staining using $KMnO_4$. All chemicals used, including all iodide and bromide substrates **4** and **6**, were obtained from commercial sources, unless otherwise stated. Dry solvents were obtained from the University of Oxford internal solvent drying system (Innovative Technology Inc. PS-400-7), wherein they are purified through dried alumina columns. Dry DMSO was purchased from Fischer, stored over molecular sieves and fitted with an AcroSeal™. Solvents used were degassed thoroughly by vigorously bubbling with a stream of N_2 for a minimum of 20 minutes prior to use. Solvents used for purification, or not needed dry, were reaction grade from either Honeywells or Sigma Aldrich. When referring to the solvent 'Petrol', what is meant is the fractions of petroleum ether which boil within the range 40 – 60 °C. Flash column chromatography was performed by dry loading the compound onto celite and adding this to a slurried column, using Geduran® Si 60, 40-63 micron silica gel, and the indicated eluent system.

^{1}H, ^{13}C and ^{19}F NMR spectra were recorded on a Brüker AVIII 400 Ultrashield spectrometer in deuterated solvent at the stated operating frequency. ^{13}C NMR spectra were recorded using the ^{1}H decoupling method; ^{19}F NMR spectra were recorded using the ^{1}H decoupling method. Acquisitions were carried out at room temperature unless otherwise stated. Chemical shifts (δ/ ppm) are reported in parts per million (ppm) and referenced relative to the residual solvent peak ($CDCl_3$: δH = 7.26 ppm, δC = 77.16 ppm; $(CD_3)_2SO$: δH = 2.50 ppm, δ = 39.52 ppm). ^{19}F NMR spectra were referenced externally to $CFCl_3$ (δF = 0.0 ppm). Chemical shifts from ^{1}H NMR and ^{19}F NMR spectrometry are recorded to two decimal places, whereas chemical shifts from ^{13}C NMR spectrometry are recorded to 1 decimal place. Coupling constants (*J*) are given in Hertz (Hz). Multiplicity is assigned using the following abbreviations: singlet (s), doublet (d), triplet (t), quartet (q), quintet (quin), multiplet (m), broad (br), apparent (app.).

High resolution mass spectra were obtained on an ACQUITY I-Class PLUS UPLC System (Waters, Milford, MA, USA) coupled to an ACQUITY RDa mass spectrometer (Waters, Milford, MA, USA) equipped with an ESI probe, in positive ion mode with a TOF mass analyzer. Values quoted are a ratio of mass to charge in Daltons to four decimal places. The mass found was compared to the mass calculated from the monoisotopic molecular formula, and all results were found to be within a 5 ppm error of the calculated values.

Melting point values were recorded in degrees Celsius (°C) using a STUART Scientific Melting Point Apparatus SMP1 scientific hot-stage microscope. Infrared spectra were determined neat using a Brüker Tensor 27 FT-IR spectrometer with an internal range of 600 – 4000 cm^{-1} and all absorptions are given in wavenumbers to the nearest whole number (cm^{-1}). Compound names are generated by PerkinElmer ChemDraw Professional.

For high throughput reaction screening, LC/MS analysis used a 0.1% $AcOH/NH_4CO_2H/H_2O$ based gradient over 0.8 min running from 5-95% MeCN using a Waters Acquity UPLC BEH C18 30 × 2.1 mm column at 100 °C with a flow rate of 2.5 mL min^{-1} and a detection wavelength of 210-360 nm. 0.5 µL injections were made directly from diluted reaction mixtures and ionization monitored in just positive mode.

2. Optimisation of copper-catalyzed reactions

2.1. Optimisation of coupling from iodides

Table 1. Ligand and base equivalents screen

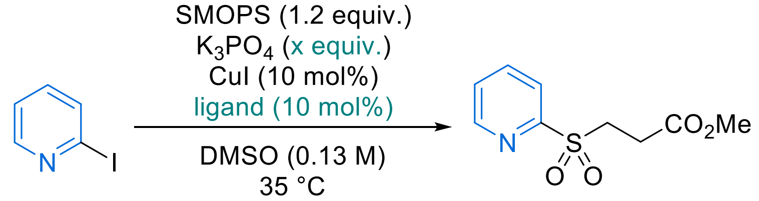

4a (1.0 equiv.) **5a**

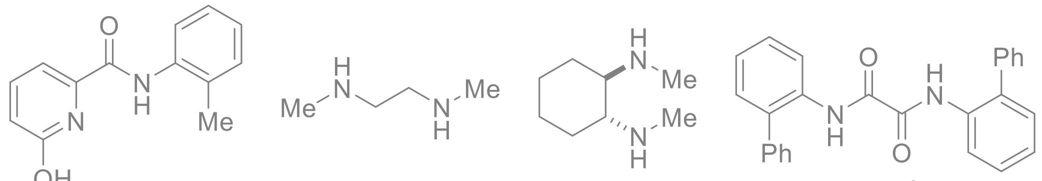

L2[a] **L3** **L4** **L5**[b]

Entry	Ligand	K_3PO_4 equiv.	NMR yield 4a remaining (%)	NMR yield 5a (%)
**1**	L2	1.0	50	**12**
**2**	L2	0.1	70	**20**
**3**	L2	0	76	**20**
**4**	L3	1.0	14	**22**
**5**	L3	0.1	31	**57**
**6**	L3	0	18	**66**
**7**	L4	1.0	7	**32**
**8**	L4	0.1	9	**64**
**9**	L4	0	10	**63**
**10**[c]	L5	0	9	**46**

Reactions performed on 0.2 mmol scale. NMR yields determined by quantitative ^{1}H NMR spectroscopy of crude reaction mixture using 1,3,5-trimethoxybenzene as an internal standard. [a] Prepared according to procedure outlined by Singer *et al.*[1] [b] Prepared according to procedure outlined by Ma *et al.*[2] [c] Reaction performed at 50 °C.

Table 2. Temperature screen

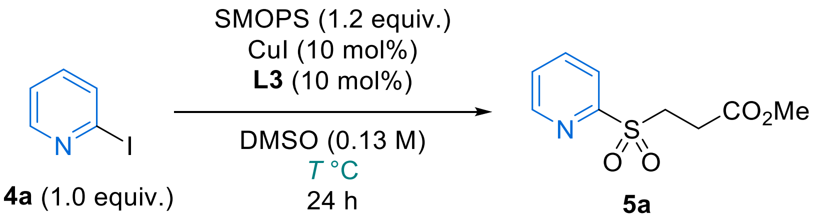

Entry	Temp (°C)	NMR yield 4a remaining (%)	NMR yield 5a (%)
**1**	35	18	**66**
**2**	50	9	**85**

Reactions performed on 0.2 mmol scale. NMR yields determined by quantitative ^{1}H NMR spectroscopy of crude reaction mixture using 1,3,5-trimethoxybenzene as an internal standard.

2.2. Optimisation of coupling from bromides

Note: Initial optimisation was performed using SMOPS (**2**) to give sulfone **7b***.

Table 3. Ligand screen

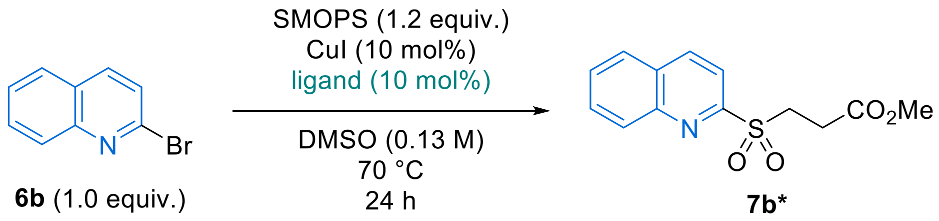

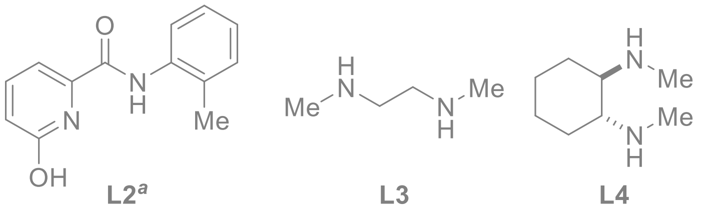

Entry	Ligand	Additive	NMR yield 6b remaining (%)	NMR yield 7b* (%)
**1**	**L2**	None	46	**28**
**2**	**L2**	K_3PO_4 (10 mol%)	60	**40**
**3**	**L3**	None	33	**53**
**4**	**L3**	3Å MS	45	**36**
**5**	**L3**	NaI (2.0 equiv.)	42	**41**
**6**	**L3**	SMOPS (2.0 equiv.)	13	**55**
**7**	**L4**	None	14	**66**

Reactions performed on 0.2 mmol scale. NMR yields determined by quantitative ^{1}H NMR spectroscopy of crude reaction mixture using 1,3,5-trimethoxybenzene as an internal standard. [a] Prepared according to procedure outlined by Singer *et al.*[1]

Table 4. Copper source screen

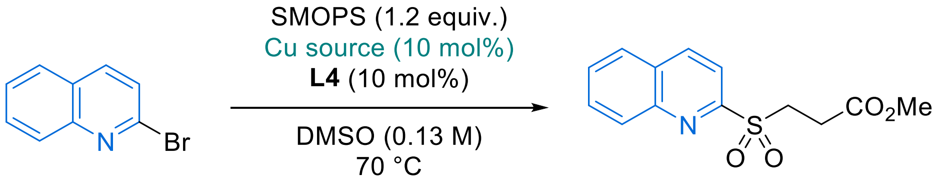

6b (1.0 equiv.) **7b***

Entry	Copper source	NMR yield 6b remaining (%)	NMR yield 7b* (%)
**1**	CuI	14	**66**
**2**	CuBr	15	**37**
**3**	Cu_2O	17	**44**

Reactions performed on 0.2 mmol scale. NMR yields determined by quantitative ^{1}H NMR spectroscopy of crude reaction mixture using 1,3,5-trimethoxybenzene as an internal standard.

Table 5. Temperature and time screen

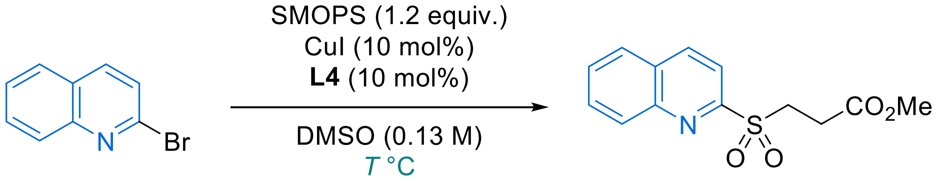

6b (1.0 equiv.) **7b***

Entry	Temp. (°C)	Time (h)	NMR yield 6b remaining (%)	NMR yield 7b* (%)
**1**	70	18	19	**60**
**2**	70	24	14	**66**
**3**	70	48	20	**50**
**4**	90	18	18	**43**

Reactions performed on 0.2 mmol scale. NMR yields determined by quantitative ^{1}H NMR spectroscopy of crude reaction mixture using 1,3,5-trimethoxybenzene as an internal standard.

Table 6. Sulfonylation reagent screen

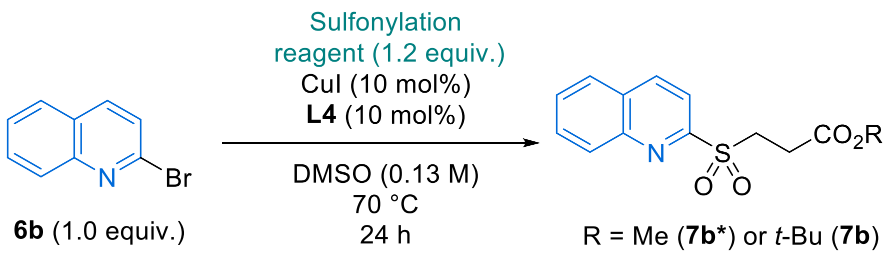

Entry	Sulfonylation reagent	NMR yield 6b remaining (%)	NMR yield 7b*/7b (%)
1	SMOPS **2** (R = Me)	14	**66**
2	STOPS **3** (R = *t*-Bu)	10	**84**

Reactions performed on 0.2 mmol scale. NMR yields determined by quantitative ^{1}H NMR spectroscopy of crude reaction mixture using 1,3,5-trimethoxybenzene as an internal standard.

3. Coupling from aryl chloride

Table 7. Aryl chloride coupling screen

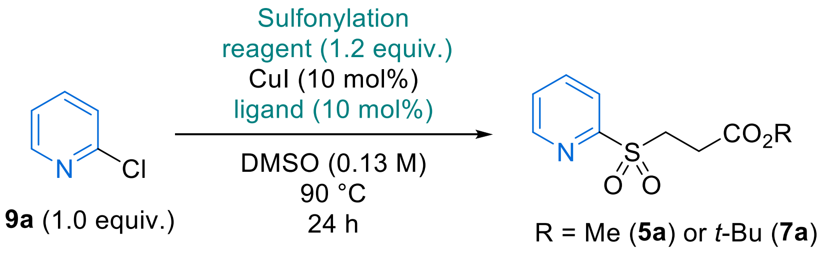

Entry	Sulfonylation reagent	Ligand	NMR yield 9a remaining (%)	NMR yield 5a/7a (%)
1	SMOPS **2** (R = Me)	**L3**	29	**2**
2	SMOPS **2** (R = Me)	**L4**	30	**4**
3	STOPS **3** (R = *t*-Bu)	**L3**	53	**9**
4	STOPS **3** (R = *t*-Bu)	**L4**	46	**15**

Reactions performed on 0.2 mmol scale. NMR yields determined by quantitative ^{1}H NMR spectroscopy of crude reaction mixture using 1,3,5-trimethoxybenzene as an internal standard.

Reactions from chloride substrates unsuccessful and not pursued further.

4. Copper-catalyzed coupling conditions high throughput screen

4.1 Screening parameters

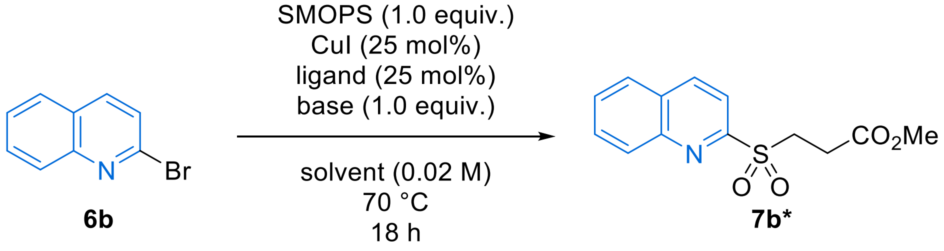

The screen examined 12 ligands, 8 bases (including no base) and 3 solvents.

Ligand	Short form ligand name	Base	Solvent
4,7-Hydroxy-1,10-phenanthroline	4,7-2OH-Phen	None	MeCN
Salicylaldoxime	Salicylaldoxime	$NaHCO_3$	DMSO
N-(2-Ethyl-6-methylphenyl)-oxalamic acid	DMPAO-EtMe	Na_2CO_3	DMA
N-(2-Cyanophenyl)pyridine-2-carboxamide	AbbvieLigand	DIPEA	
1,3-Di(2-pyridyl)-1,3-propanedione	1,3-Di(2-Py)-1,3-PropDione	K_3PO_4	
N-(2,6-Dimethylphenyl)pyridine-2-carboxamide	2PyCONH-2,6diMePh	K_2CO_3	
Undisclosed ligand 1	HMNPCAlt1	CsOPiv	
(*R*)-BINOL	(R)-BINOL	Cs_2CO_3	
Undisclosed ligand 2	PHMPO		
DMEDA (**L3**)	MeNHCH2CH2NHMe		
1,4,7-Triazacyclononane	1,4,7-TCN		
(*R*,*R*)-(−)-*N*,*N'*-Dimethyl-1,2-cyclohexanediamine	DMCyHDiA		

4.2 Experimental procedure

Note: All reactions were set-up and performed in a glove box and in 8 × 20 mm glass vials with pre-dispensed stirrer bars. The reactions were performed on a 0.002 mmol scale.

Example procedure

The following procedure gave the highest yield with the simplest combination of reagents without base: SMOPS **2** (0.348 mg, 0.00200 mmol, 1.0 equiv., 10.0 µL of 0.2 M solution in H_2O) and CuI (0.0952 mg, 0.00050 mmol, 25 mol%, 10 µL of 0.05 M solution in MeCN) were first dispensed before evaporation to dryness. 2-Bromoquinoline (0.416 mg, 0.00200 mmol, 1.0 equiv., 100 µL of 0.02 M solution in DMSO and internal standard) and DMEDA (**L3**) (0.0441 mg, 25 mol%, 0.00050 mmol, 25 mol%, 10 µL of 0.05 M solution in THF) were then added. The reaction was crimp sealed in the glove-box environment before being stirred at 70 °C for 18 h. After cooling to r.t. the plates were diluted with DMF (200 µL) and analyzed by LC/MS.

4.3 Results from screen

For the highest yielding reaction, the following products were identified in the LC/MS trace at 310 nm:

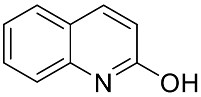

A
MW146+ve
0.233 min

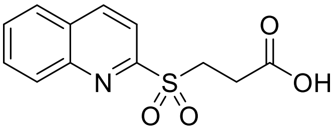

B
MW266+ve
0.261 min

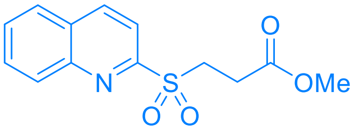

C (**7b***)
MW280+ve
0.338 min

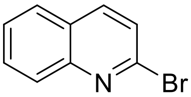

D (**6b**)
MW208/210+ve
0.403 min

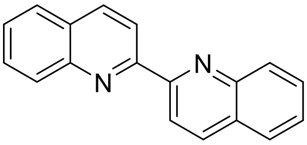

E
MW257+ve
0.573 min

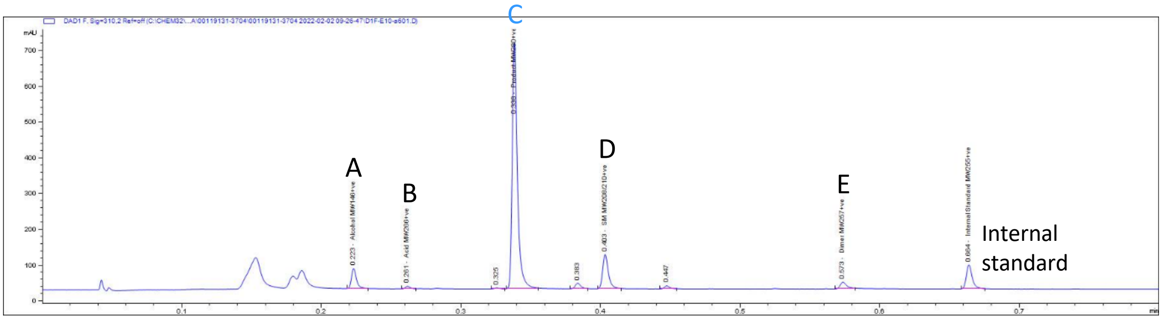

Summary of all reactions

The following graph (presented as a Spotfire on the next page) summarizes all reactions performed in the screen. Spotfire presents yield **7b*** by %Area UV (vertical axis) vs. yield **7b*** by SIM ion count (horizontal axis) in excellent correlation. The results are separated by solvent (see title at top of each graph panel), the base used is represented by shape, ligands are labelled by color and name. The highest vertical spot represents the recommended conditions (simplest and best: DMSO as solvent, no base, DMEDA (**L3**) as ligand).

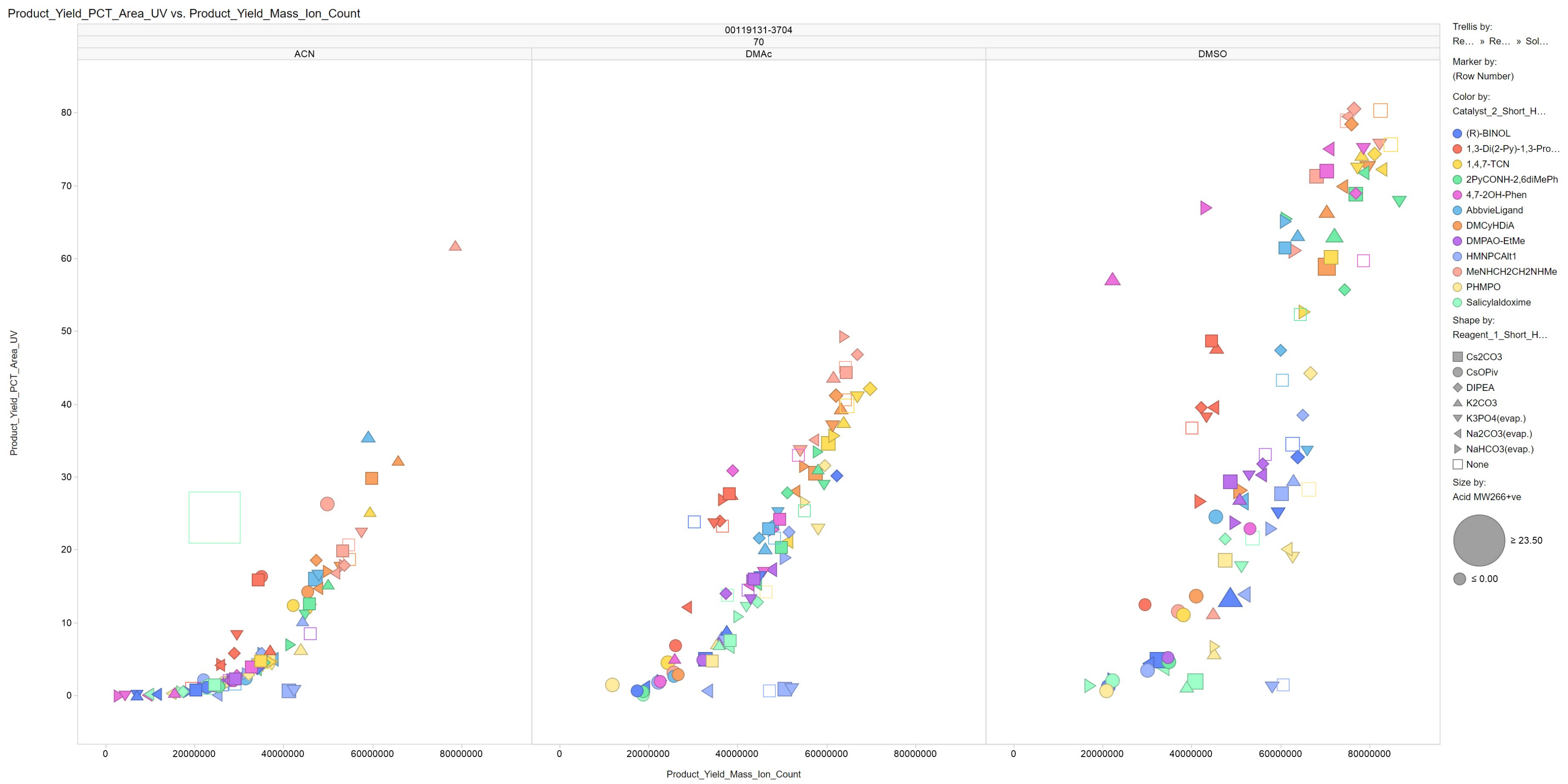
Product_Yield_PCT_Area_UV vs. Product_Yield_Mass_Ion_Count
00119131-3704
70
ACN
DMAc
DMSO
Product_Yield_PCT_Area_UV
Product_Yield_Mass_Ion_Count
Trellis by:
Re… » Re… » Sol…
Marker by:
(Row Number)
Color by:
Catalyst_2_Short_H…
(R)-BINOL
1,3-Di(2-Py)-1,3-Pro…
1,4,7-TCN
2PyCONH-2,6diMePh
4,7-2OH-Phen
AbbvieLigand
DMCyHDiA
DMPAO-EtMe
HMNPCAlt1
MeNHCH2CH2NHMe
PHMPO
Salicylaldoxime
Shape by:
Reagent_1_Short_H…
Cs2CO3
CsOPiv
DIPEA
K2CO3
K3PO4(evap.)
Na2CO3(evap.)
NaHCO3(evap.)
None
Size by:
Acid MW266+ve
≥ 23.50
≤ 0.00

5. Experimental procedures

5.1. Synthesis of sulfonylation reagents

Three step synthesis of sodium 3-methoxy-3-oxopropane-1-sulfinate, SMOPS (2)

Adapted from procedure described by Baskin *et al.*[3]

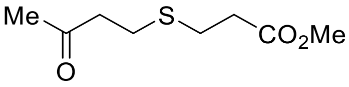

Methyl 3-((3-oxobutyl)thio)propanoate (2a)

Methyl 3-mercaptopropionate (22.2 mL, 200 mmol, 1.0 equiv.) and methyl vinyl ketone (18.3 mL, 220 mmol, 1.1 equiv.) were added to an oven-dried 1 L round-bottom flask fitted with a suba-seal and the vessel was vacuum/N_2 purged (× 3), before adding anhydrous, degassed THF (300 mL) and anhydrous Et_3N (2.8 mL, 20 mmol, 0.1 equiv.). After heating in an aluminium heating block for 5 h at 50 °C, the solvent was removed *in vacuo* to yield the crude sulfide product as a brown oil (38.0 g, quantitative) which was used in the next step without purification.

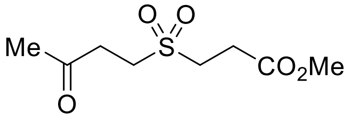

Methyl 3-((3-oxobutyl)sulfonyl)propanoate (2b)

Methyl 3-((3-oxobutyl)thio)propanoate (**2a**) (38.0 g, 200 mmol, 1.0 equiv.) was added to a 500 mL three-necked round-bottom flask, with $Na_2WO_4{\cdot}2H_2O$ (3.30 g, 10 mmol, 5 mol%), Aliquat® 336, (4.04 g, 10 mmol, 5 mol%), EtOAc (100 mL), cyclohexane (50 mL) and H_2O (20 mL). The reaction mixture was stirred and heated to 60 °C in an aluminium heating block before a 30% aqueous solution of H_2O_2 (83 mL, 655 mmol, 3.28 equiv.) was added dropwise over a 1 h period. After stirring for 4 h at 60 °C, the reaction mixture was cooled to r.t. and diluted with EtOAc (100 mL) and H_2O (100 mL). The organic layer was separated and the aqueous layer was extracted with EtOAc (2 × 50 mL). The combined organic extracts were washed with brine (50 mL), dried over Na_2SO_4, and filtered through a pad of silica (~4 cm). After being concentrated *in vacuo*, the crude product was stirred as a suspension in EtOAc (50 mL) and Petrol (50 mL) for 18 h and collected by filtration to yield the product as a white fluffy solid (26.6 g, 60%).

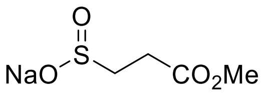

Sodium 3-methoxy-3-oxopropane-1-sulfinate, SMOPS (2)

A freshly made solution of NaOMe was prepared by adding sodium metal (258 mg, 11.2 mmol, 1.0 equiv.) to anhydrous MeOH (25 mL) in an oven-dried 50 mL round-bottom flask fitted with a $CaCl_2$ drying tube. Once all the sodium had dissolved, the NaOMe solution was added dropwise to a solution of methyl 3-((3-oxobutyl)sulfonyl)propanoate (**2b**) (2.50 g, 11.2 mmol, 1.0 equiv.) in anhydrous, degassed THF (120 mL). The reaction mixture was stirred at r.t. for 2 h, then concentrated *in vacuo*. The resulting white solid was stirred as a suspension in Et_2O and EtOAc for 2 h, then filtered and

washed with further Et_2O and EtOAc. Drying by vacuum filtration yielded the final sulfinate product as a white powder (1.60 g, 82%).

^{1}H NMR (400 MHz, DMSO-d_6) δ 3.55 (s, 3H), 2.40 (t, *J* = 8.0 Hz, 2H), 2.01 (t, *J* = 8.0 Hz, 2H); **^{13}C NMR** (101 MHz, DMSO-d_6) δ 173.9, 56.0, 51.2, 26.0; **HRMS** (ESI) *m/z*: $[M\text{-}Na]^-$ calcd for $C_4H_7O_4S^-$ 151.0060, found 151.0061; **R_f** = 0.0 (10% MeOH in CH_2Cl_2). Data is consistent with literature.[3]

Three step synthesis of sodium 3-(*tert*-butoxy)-3-oxopropane-1-sulfinate, STOPS (3)

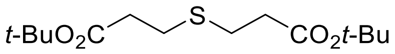

Di-*tert*-butyl 3,3'-thiodipropionate (3a)

Adapted from procedure described by Detty *et al.*[4]

tert-Butyl acrylate (29.3 mL, 200 mmol, 2.0 equiv.) was firstly dissolved in MeOH (200 mL), then a solution of $NaSH{\cdot}H_2O$ (13.8 g, 150 mmol, 1.5 equiv.) in water (100 mL) and aqueous sat. $NaHCO_3$ (100 mL) was added. The reaction mixture was stirred at r.t. for 1 h, then diluted with water (400 mL) and extracted with Et_2O (3 × 150 mL). The combined organic extracts were washed with brine (50 mL), dried over Na_2SO_4, and concentrated *in vacuo* to yield the crude sulfide product as a pale brown oil (29.0 g, 83%) which was used in the next step without purification.

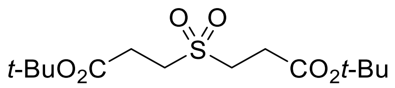

Di-*tert*-butyl 3,3'-sulfonyldipropionate (3b)

Adapted from procedure described by Baskin *et al.*[3]

Di-*tert*-butyl 3,3'-thiodipropionate (**3a**) (24.0 g, 82.6 mmol, 1.0 equiv.) was placed in a 500 mL three-necked round-bottom flask, with $Na_2WO_4{\cdot}2H_2O$ (1.35 g, 4.1 mmol, 5 mol%), Aliquat® 336 tricaprylylmethylammonium chloride, (1.65 g, 4.1 mmol, 5 mol%), EtOAc (40 mL), cyclohexane (20 mL) and H_2O (10 mL). The reaction mixture was stirred and heated to 60 °C in an aluminium heating block, before a 30% aqueous solution of H_2O_2 (34 mL, 270.9 mmol, 3.28 equiv.) was added dropwise over a 1 h period. After being stirred for 4 h at 60 °C, the reaction mixture was cooled to r.t. and diluted with EtOAc (100 mL) and H_2O (100 mL). The organic layer was separated and the aqueous layer was extracted with EtOAc (2 × 50 mL). The combined organic extracts were washed with brine (50 mL), dried over Na_2SO_4, and filtered through a pad of silica (~4 cm). After being concentrated *in vacuo*, the crude product was stirred as a suspension in a mixture of EtOAc (50 mL) and Petrol (50 mL) for 18 h and collected by filtration to yield the product as a white fluffy solid (20.2 g, 76%).

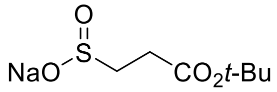

Sodium 3-(*tert*-butoxy)-3-oxopropane-1-sulfinate, STOPS (3)

To an oven-dried round-bottom flask was added di-*tert*-butyl 3,3'-sulfonyldipropionate (**3b**) (356.3 mg, 1.1 mmol, 1.0 equiv.) and NaO*t*-Bu (105.7 mg, 1.1 mmol, 1.0 equiv.). The vessel was vacuum/N_2 purged (× 3), before adding anhydrous *t*-BuOH (10 mL, 0.11 M), and the reaction mixture was stirred at 50 °C in an aluminium heating block for 3 h. The reaction mixture was concentrated *in vacuo*,

washed with Et_2O and Petrol, then collected by vacuum filtration to give the final sulfinate product as a white solid (169.5 mg, 72%).

^{1}H NMR (400 MHz, DMSO-d_6) δ 2.26 (t, *J* = 8.7 Hz, 2H), 1.93 (t, *J* = 8.1 Hz, 2H), 1.38 (s, 9H); **^{13}C NMR** (101 MHz, DMSO-d_6) δ 172.8, 79.3, 56.4, 27.8, 27.6; **HRMS** (ESI) *m/z*: $[M-Na]^-$ calcd for $C_7H_{13}O_4S^-$ 193.0540, found 193.0548; **IR** $_{vmax}$ (neat)/cm^{-1} 1718, 1366, 1232, 1155, 1039, 988, 843, 682; **mp** 160 – 162 °C; **R_f** = 0.1 (10% MeOH in CH_2Cl_2).

5.2. Substrate Scopes

5.2.1. General procedure A: Cu-catalysed sulfone formation from iodides

To an oven-dried microwave vial (10 mL) aryl iodide (if solid) (0.20 mmol, 1.0 equiv.), SMOPS (41.8 mg, 0.24 mmol, 1.2 equiv.) and CuI (3.8 mg, 0.02 mmol, 10 mol%) were added. The vial was capped and vacuum/N_2 purged (× 3), before adding anhydrous, degassed DMSO (1.5 mL, 0.13 M), aryl iodide (if liquid) (0.20 mmol, 1.0 equiv.) and **L3** (2 µL, 0.02 mmol, 10 mol%). The vial was further sealed with parafilm, then stirred at 50 °C in an aluminium heating block for 24 h. The reaction mixture was then cooled to r.t. and 10 mL of water was added. The product was extracted from the aqueous with EtOAc (3 × 10 mL). The combined organic extracts were washed with brine, dried over Na_2SO_4 and concentrated *in vacuo*. The crude product was purified by flash column chromatography (EtOAc in Petrol) to afford the desired aryl sulfone product.

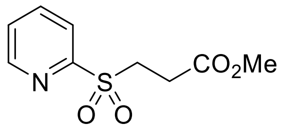

Methyl 3-(pyridin-2-ylsulfonyl)propanoate (5a)

Prepared according to **general procedure A**: 2-iodopyridine (21 µL, 0.20 mmol, 1.0 equiv.), SMOPS (41.8 mg, 0.24 mmol, 1.2 equiv.), CuI (3.8 mg, 0.02 mmol, 10 mol%) and **L3** (2 µL, 0.02 mmol, 10 mol%) were dissolved in DMSO (1.5 mL, 0.13 M). The reaction mixture was stirred at 50 °C for 24 h. After aqueous work up, the crude product was purified by flash column chromatography on silica gel (50% EtOAc in Petrol) to give the product as a colourless oil (39.0 mg, 85%).

Also prepared on a 1 mmol scale: SMOPS (209.0 mg, 1.2 mmol, 1.2 equiv.) and CuI (19.0 mg, 0.1 mmol, 10 mol%) were added to an oven-dried round-bottom flask (25 mL). The reaction vessel was fitted with a suba-seal and vacuum/N_2 purged (× 3), before adding anhydrous, degassed DMSO (7.5 mL, 0.13 M), 2-iodopyridine (106 µL, 1.0 mmol, 1.0 equiv.) and **L3** (11 µL, 0.1 mmol, 10 mol%). The reaction mixture was heated to 50 °C in an aluminium heating block and stirred for 24 h, after which the mixture was quenched with water (15 mL). The product was extracted from the aqueous with EtOAc (3 × 15 mL), and the combined organics were washed with brine (15 mL) and dried over Na_2SO_4, before concentrating *in vacuo*. The crude product was purified by flash column chromatography on silica gel (50% EtOAc in Petrol) to give the product as a colourless oil (192.6 mg, 84%).

^{1}H NMR (400 MHz, $CDCl_3$) δ 8.73 (ddd, *J* = 4.7, 1.7, 0.9 Hz, 1H), 8.07 (dt, *J* = 7.8, 1.1 Hz, 1H), 7.97 (td, *J* = 7.8, 1.7 Hz, 1H), 7.57 (ddd, *J* = 7.6, 4.7, 1.2 Hz, 1H), 3.74 – 3.69 (m, 2H), 3.66 (s, 3H), 2.85 – 2.80 (m, 2H); **^{13}C NMR** (101 MHz, $CDCl_3$) δ 170.6, 157.0, 150.4, 138.5, 127.8, 122.3, 52.5, 47.7, 27.5; **HRMS** (ESI) *m/z*: $[M+Na]^+$ calcd for $C_9H_{11}NO_4SNa^+$ 252.0301, found 252.0299; **R_f** = 0.32 (60% EtOAc in Petrol). Data is consistent with the literature.[5]

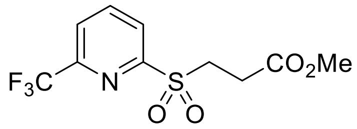

Methyl 3-((6-(trifluoromethyl)pyridin-2-yl)sulfonyl)propanoate (5b)

Prepared according to **general procedure A**: 2-iodo-6(trifluoromethyl)pyridine (54.6 mg, 0.20 mmol, 1.0 equiv.), SMOPS (41.8 mg, 0.24 mmol, 1.2 equiv.), CuI (3.8 mg, 0.02 mmol, 10 mol%) and **L3** (2 µL, 0.02 mmol, 10 mol%) were dissolved in DMSO (1.5 mL, 0.13 M). The reaction mixture was stirred at 50 °C for 24 h. After aqueous work up, the crude product was purified by flash column chromatography on silica gel (30 – 40% EtOAc in Petrol) to give the product as a colourless oil (40.5 mg, 68%).

^{1}H NMR (400 MHz, $CDCl_3$) δ 8.26 (dd, *J* = 7.9, 1.1 Hz, 1H), 8.20 (td, *J* = 7.9, 0.7 Hz, 1H), 7.93 (dd, *J* = 7.7, 1.1 Hz, 1H), 3.78 (app. dd, *J* = 8.0, 7.1 Hz, 2H), 3.68 (s, 3H), 2.89 (app. dd, *J* = 8.0, 7.1 Hz, 2H); **^{13}C NMR** (151 MHz, $CDCl_3$) δ 170.5, 158.0, 148.8 (q, $^2J_{C-F}$ = 36.6 Hz), 140.5, 124.4 (q, $^3J_{C-F}$ = 2.5 Hz), 120.7 (q, $^1J_{C-F}$ = 274.6 Hz), 52.6, 47.5, 29.9, 27.4; **^{19}F NMR** (377 MHz, $CDCl_3$) δ −67.8; **HRMS** (ESI) *m/z*: $[M+Na]^+$ calcd for $C_{10}H_{10}F_3NO_4SNa^+$ 320.0175, found 320.0176; **IR** $_{vmax}$ (neat)/cm^{-1} 1744, 1441, 1340, 1200, 1159, 1127, 1085, 825, 745; **R$_f$** = 0.34 (30% EtOAc in Petrol).

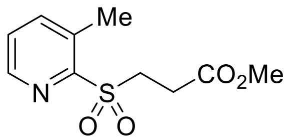

Methyl 3-((3-methylpyridin-2-yl)sulfonyl)propanoate (5c)

Prepared according to **general procedure A**: 2-iodo-3-methylpyridine (24 µL, 0.20 mmol, 1.0 equiv.), SMOPS (41.8 mg, 0.24 mmol, 1.2 equiv.), CuI (3.8 mg, 0.02 mmol, 10 mol%) and **L3** (2 µL, 0.02 mmol, 10 mol%) were dissolved in DMSO (1.5 mL, 0.13 M). The reaction mixture was stirred at 50 °C for 24 h. After aqueous work up, the crude product was purified by flash column chromatography on silica gel (40% EtOAc in Petrol) to give the product as a white solid (35.5 mg, 73%).

^{1}H NMR (400 MHz, $CDCl_3$) δ 8.41 (ddd, *J* = 4.6, 1.6, 0.7 Hz, 1H), 7.69 (ddd, *J* = 7.8, 1.6, 0.8 Hz, 1H), 7.41 (dd, *J* = 7.8, 4.6 Hz, 1H), 3.95 – 3.90 (m, 2H), 3.73 (s, 3H), 2.99 – 2.94 (m, 2H), 2.71 (s, 3H); **^{13}C NMR** (101 MHz, $CDCl_3$) δ 171.1, 155.8, 146.0, 141.6, 133.2, 127.0, 52.4, 47.6, 28.1, 18.0; **HRMS** (ESI) *m/z*: $[M+Na]^+$ calcd for $C_{10}H_{13}NO_4SNa^+$ 266.0458, found 266.0464; **IR** $_{vmax}$ (neat)/cm^{-1} 1742, 1565, 1440, 1364, 1308, 1157, 1122, 826, 803, 743; **mp** 44 – 46 °C; **R$_f$** = 0.43 (40% EtOAc in Petrol).

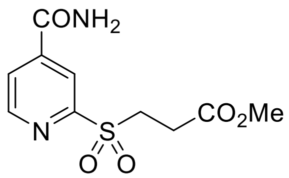

Methyl 3-((4-carbamoylpyridin-2-yl)sulfonyl)propanoate (5d)

Prepared according to **general procedure A**: 2-iodopyridine-4-carboxamide (49.6 mg, 0.20 mmol, 1.0 equiv.), SMOPS (41.8 mg, 0.24 mmol, 1.2 equiv.), CuI (3.8 mg, 0.02 mmol, 10 mol%) and **L3** (2 µL, 0.02 mmol, 10 mol%) were dissolved in DMSO (1.5 mL, 0.13 M). The reaction mixture was stirred at 50 °C for 24 h. After aqueous work up, the crude product was purified by flash column

chromatography on silica gel (80 – 90% EtOAc in Petrol) to give the product as an off-white fluffy solid (38.2 mg, 70%).

^{1}H NMR (400 MHz, DMSO) δ 8.95 (dd, *J* = 4.9, 0.8 Hz, 1H), 8.54 (s, 1H), 8.40 (dd, *J* = 1.6, 0.8 Hz, 1H), 8.13 (dd, *J* = 4.9, 1.6 Hz, 1H), 7.97 (s, 1H), 3.75 (t, *J* = 7.2 Hz, 2H), 3.55 (s, 3H), 2.75 (t, *J* = 7.2 Hz, 2H); **^{13}C NMR** (101 MHz, DMSO) δ 170.3, 164.7, 157.0, 151.3, 143.9, 125.8, 119.7, 51.9, 47.4, 27.2; **HRMS** (ESI) *m/z*: $[M+Na]^+$ calcd for $C_{10}H_{12}N_2O_5SNa^+$ 295.0359, found 295.0361; **IR** $_{vmax}$ (neat)/cm^{-1} 3654, 3449, 3185, 1733, 1694, 1620, 1547, 1442, 1399, 1319, 1256, 1177, 1136, 1116, 994, 911, 848, 783; **mp** 108 – 110 °C; **R$_f$** = 0.22 (80% EtOAc in Petrol).

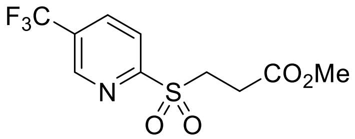

Methyl 3-((5-(trifluoromethyl)pyridin-2-yl)sulfonyl)propanoate (5e)

Prepared according to **general procedure A**: 2-iodo-5-(trifluoromethyl)pyridine (54.6 mg, 0.20 mmol, 1.0 equiv.), SMOPS (41.8 mg, 0.24 mmol, 1.2 equiv.), CuI (3.8 mg, 0.02 mmol, 10 mol%) and **L3** (2 µL, 0.02 mmol, 10 mol%) were dissolved in DMSO (1.5 mL, 0.13 M). The reaction mixture was stirred at 50 °C for 24 h. After aqueous work up, the crude product was purified by flash column chromatography on silica gel (20% EtOAc in Petrol) to give the product as a white solid (41.3 mg, 70%).

^{1}H NMR (400 MHz, $CDCl_3$) δ 9.00 (s, 1H), 8.28 – 8.20 (m, 2H), 3.79 (app. dd, *J* = 8.2, 7.1 Hz, 2H), 3.69 (s, 3H), 2.87 (app. dd, *J* = 8.2, 7.1 Hz, 2H); **^{13}C NMR** (151 MHz, $CDCl_3$) δ 170.4, 160.3, 147.4 (q, $^{3}J_{C\text{-}F}$ = 3.9 Hz), 136.1 (q, $^{3}J_{C\text{-}F}$ = 3.4 Hz), 130.4 (q, $^{2}J_{C\text{-}F}$ = 34.0 Hz), 122.6 (q, $^{1}J_{C\text{-}F}$ = 273.2 Hz), 122.1, 52.6, 47.6, 27.4; **^{19}F NMR** (377 MHz, $CDCl_3$) δ -62.68; **HRMS** (ESI) *m/z*: $[M+Na]^+$ calcd for $C_{10}H_{10}F_3NO_4SNa^+$ 320.0175, found 320.0182; **R$_f$** = 0.19 (30% EtOAc in Petrol). Data is consistent with the literature.[5]

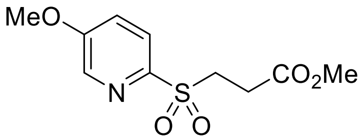

Methyl 3-((5-methoxypyridin-2-yl)sulfonyl)propanoate (5f)

Prepared according to **general procedure A**: 2-iodo-5-methoxypyridine (47.0 mg, 0.20 mmol, 1.0 equiv.), SMOPS (41.8 mg, 0.24 mmol, 1.2 equiv.), CuI (3.8 mg, 0.02 mmol, 10 mol%) and **L3** (2 µL, 0.02 mmol, 10 mol%) were dissolved in DMSO (1.5 mL, 0.13 M). The reaction mixture was stirred at 50 °C for 24 h. After aqueous work up, the crude product was purified by flash column chromatography on silica gel (20% EtOAc in Petrol) to give the product as a colourless oil (45.6 mg, 88%).

^{1}H NMR (400 MHz, $CDCl_3$) δ 8.38 (dd, *J* = 2.9, 0.6 Hz, 1H), 8.03 (dd, *J* = 8.7, 0.6 Hz, 1H), 7.34 (dd, *J* = 8.7, 2.9 Hz, 1H), 3.95 (s, 3H), 3.67 (s, 3H), 3.70 – 3.63 (m, 2H), 2.87 – 2.75 (m, 2H); **^{13}C NMR** (101 MHz, $CDCl_3$) δ 170.7, 158.7, 148.2, 139.2, 124.2, 120.4, 56.2, 52.4, 48.3, 27.6; **HRMS** (ESI) *m/z*: $[M+Na]^+$ calcd for $C_{10}H_{13}NO_5SNa^+$ 282.0407, found 282.0414; **R$_f$** = 0.31 (50% EtOAc in Petrol). Data is consistent with the literature.[5]

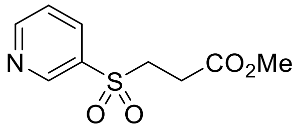

Methyl 3-(pyridin-3-ylsulfonyl)propanoate (5g)

Prepared according to **General procedure A**: 3-iodopyridine (41.0 mg, 0.20 mmol, 1.0 equiv.), SMOPS (41.8 mg, 0.24 mmol, 1.2 equiv.), CuI (3.8 mg, 0.02 mmol, 10 mol%) and **L3** (2 µL, 0.02 mmol, 10 mol%) were dissolved in DMSO (1.5 mL, 0.13 M). The reaction mixture was stirred at 50 °C for 24 h. After aqueous work up, the crude product was purified by flash column chromatography on silica gel (30% EtOAc in Petrol) to give the product as an off-white solid (32.1 mg, 70%).

^{1}H NMR (400 MHz, $CDCl_3$) δ 9.12 (dd, *J* = 2.4, 0.8 Hz, 1H), 8.91 (dd, *J* = 4.8, 1.7 Hz, 1H), 8.21 (ddd, *J* = 8.0, 2.4, 1.6 Hz, 1H), 7.54 (ddd, *J* = 8.0, 4.8, 0.8 Hz, 1H), 3.65 (s, 3H), 3.49 (t, *J* = 7.5 Hz, 2H), 2.81 (t, *J* = 7.5 Hz, 2H); **^{13}C NMR** (101 MHz, $CDCl_3$) δ 170.3, 154.8, 149.4, 136.2, 135.3, 124.1, 52.6, 52.0, 27.5; **HRMS** (ESI) *m/z*: $[M+Na]^+$ calcd for $C_9H_{11}NO_4SNa^+$ 252.0301, found 252.0312; **IR** $_{vmax}$ (neat)/cm^{-1} 1738, 1576, 1419, 1317, 1259, 1198, 1159, 1103, 1022, 804, 757, 704, 620; **mp** 60 – 64 °C; **R$_f$** = 0.35 (100% EtOAc).

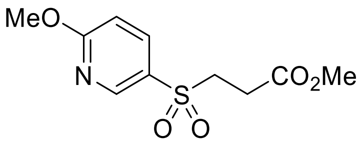

Methyl 3-((6-methoxypyridin-3-yl)sulfonyl)propanoate (5h)

Prepared according to **General procedure A**: 5-iodo-2-methoxypyridine (47.0 mg, 0.20 mmol, 1.0 equiv.), SMOPS (41.8 mg, 0.24 mmol, 1.2 equiv.), CuI (3.8 mg, 0.02 mmol, 10 mol%) and **L3** (2 µL, 0.02 mmol, 10 mol%) were dissolved in DMSO (1.5 mL, 0.13 M). The reaction mixture was stirred at 50 °C for 24 h. After aqueous work up, the crude product was purified by flash column chromatography on silica gel (30% EtOAc in Petrol) to give the product as a white solid (41.5 mg, 80%).

^{1}H NMR (400 MHz, $CDCl_3$) δ 8.68 (dd, *J* = 2.6, 0.7 Hz, 1H), 7.99 (dd, *J* = 8.8, 2.6 Hz, 1H), 6.87 (dd, *J* = 8.8, 0.7 Hz, 1H), 4.02 (s, 3H), 3.66 (s, 3H), 3.43 (app. dd, *J* = 8.1, 7.1 Hz, 2H), 2.78 (app. dd, *J* = 8.1, 7.1 Hz, 2H); **^{13}C NMR** (101 MHz, $CDCl_3$) δ 170.5, 167.5, 149.1, 138.2, 127.9, 111.9, 54.7, 52.5, 52.2, 27.8; **HRMS** (ESI) *m/z*: $[M+Na]^+$ calcd for $C_{10}H_{13}NO_5SNa^+$ 282.0407, found 282.0414; **R$_f$** = 0.43 (35% EtOAc in Petrol). Data is consistent with the literature.[5]

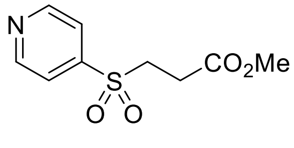

Methyl 3-(pyridin-4-ylsulfonyl)propanoate (5i)

Prepared according to **General procedure A**: 4-iodopyridine (41.0 mg, 0.20 mmol, 1.0 equiv.), SMOPS (41.8 mg, 0.24 mmol, 1.2 equiv.), CuI (3.8 mg, 0.02 mmol, 10 mol%) and **L3** (2 µL, 0.02 mmol, 10 mol%) were dissolved in DMSO (1.5 mL, 0.13 M). The reaction mixture was stirred at 50 °C for 24 h. After aqueous work up, the crude product was purified by flash column chromatography on silica gel (70% EtOAc in Petrol) to give the product as a yellow crystalline solid (37 mg, 80%).

^{1}H NMR (400 MHz, CDCl3) δ 8.94 (dd, *J* = 4.2, 1.6 Hz, 2H), 7.78 (dd, *J* = 4.2, 1.6 Hz, 2H), 3.66 (s, 3H), 3.47 (t, *J* = 7.3 Hz, 1H), 2.79 (t, *J* = 7.5 Hz, 2H).; **^{13}C NMR** (101 MHz, $CDCl_3$) δ 170.2, 151.6, 146.7, 121.3, 52.6, 51.2, 27.4; **HRMS** (ESI) *m/z*: $[M+Na]^+$ calcd for $C_9H_{11}NO_4SNa^+$ 252.0301, found 252.0304; **R_f** = 0.31 (75% EtOAc in Petrol). Data is consistent with literature.[5]

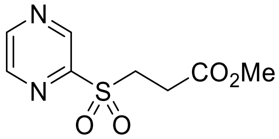

Methyl 3-(pyrazin-2-ylsulfonyl)propanoate (5j)

Prepared according to **general procedure A**: iodopyrazine (20 µL, 0.20 mmol, 1.0 equiv.), SMOPS (41.8 mg, 0.24 mmol, 1.2 equiv.), CuI (3.8 mg, 0.02 mmol, 10 mol%) and **L3** (2 µL, 0.02 mmol, 10 mol%) were dissolved in DMSO (1.5 mL, 0.13 M). The reaction mixture was stirred at 50 °C for 24 h. After aqueous work up, the crude product was purified by flash column chromatography on silica gel (50% EtOAc in Petrol) to give the product as an off-white solid (38.2 mg, 83%).

^{1}H NMR (400 MHz, $CDCl_3$) δ 9.28 (dd, *J* = 1.5, 0.4 Hz, 1H), 8.89 (dd, *J* = 2.3, 0.4 Hz, 1H), 8.73 (dd, *J* = 2.3, 1.5 Hz, 1H), 3.74 (app. dd, *J* = 8.0, 7.1 Hz, 2H), 3.68 (s, 3H), 2.88 (app. dd, *J* = 8.0, 7.1 Hz, 2H); **^{13}C NMR** (101 MHz, $CDCl_3$) δ 170.4, 152.9, 148.8, 144.6, 143.4, 52.6, 48.2, 27.3; **HRMS** (ESI) *m/z*: $[M+Na]^+$ calcd for $C_8H_{10}N_2O_4SNa^+$ 253.0254, found 253.0255; **IR** $_{vmax}$ (neat)/cm^{-1} 1743, 1398, 1327, 1255, 1181, 1017, 914, 778, 738; **mp** 74 – 78 °C; **R_f** = 0.26 (50% EtOAc in Petrol).

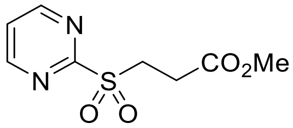

Methyl 3-(pyrimidin-2-ylsulfonyl)propanoate (5k)

Prepared according to **general procedure A**: 2-iodopyrimidine (41.2 mg, 0.20 mmol, 1.0 equiv.), SMOPS (41.8 mg, 0.24 mmol, 1.2 equiv.), CuI (3.8 mg, 0.02 mmol, 10 mol%) and **L3** (2 µL, 0.02 mmol, 10 mol%) were dissolved in DMSO (1.5 mL, 0.13 M). The reaction mixture was stirred at 50 °C for 24 h. After aqueous work up, the crude product was purified by flash column chromatography on silica gel (50% EtOAc in Petrol) to give the product as a colourless oil (35.7 mg, 78%).

^{1}H NMR (400 MHz, $CDCl_3$) δ 8.96 (d, *J* = 4.9 Hz, 2H), 7.59 (t, *J* = 4.9 Hz, 1H), 3.90 – 3.85 (m, 2H), 3.70 (s, 3H), 2.99 – 2.94 (m, 2H); **^{13}C NMR** (101 MHz, $CDCl_3$) δ 170.7, 165.7, 158.9, 124.1, 52.5, 47.1, 27.6; **HRMS** (ESI) *m/z*: $[M+Na]^+$ calcd for $C_8H_{10}N_2O_4SNa^+$ 253.0254, found 253.0261; **R_f** = 0.34 (50% EtOAc in Petrol). Data is consistent with literature.[6]

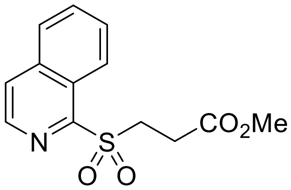

Methyl 3-(isoquinolin-1-ylsulfonyl)propanoate (5l)

Prepared according to **general procedure A**: 1-iodoisoquinoline (51.0 mg, 0.20 mmol, 1.0 equiv.), SMOPS (41.8 mg, 0.24 mmol, 1.2 equiv.), CuI (3.8 mg, 0.02 mmol, 10 mol%) and **L3** (2 µL, 0.02 mmol, 10 mol%) were dissolved in DMSO (1.5 mL, 0.13 M). The reaction mixture was stirred at 50 °C for 24 h. After aqueous work up, the crude product was purified by flash column chromatography on silica gel (30% EtOAc in Petrol) to give the product as an off-white solid (49.1 mg, 88%).

^{1}H NMR (400 MHz, $CDCl_3$) δ 8.98 – 8.91 (m, 1H), 8.47 (d, *J* = 5.5 Hz, 1H), 7.97 – 7.93 (m, 1H), 7.87 (dd, *J* = 5.5, 1.0 Hz, 1H), 7.82 (ddd, *J* = 8.2, 6.9, 1.4 Hz, 1H), 7.76 (ddd, *J* = 8.2, 6.9, 1.4 Hz, 1H), 4.11 – 4.05 (m, 2H), 3.74 (s, 3H), 3.10 – 3.01 (m, 2H); **^{13}C NMR** (101 MHz, $CDCl_3$) δ 171.1, 156.1, 140.1, 137.9, 131.6, 129.6, 127.7, 125.4, 125.1, 123.8, 52.5, 48.0, 28.2; **HRMS** (ESI) *m/z*: $[M+Na]^+$ calcd for $C_{13}H_{13}NO_4SNa^+$ 302.0458, found 302.0454; **IR** $_{vmax}$ (neat)/cm^{-1} 1741, 1583, 1439, 1367, 1308, 1251, 1126, 980, 914, 845, 787, 752; **mp** 70 – 72 °C; **R$_f$** = 0.35 (30% EtOAc in Petrol).

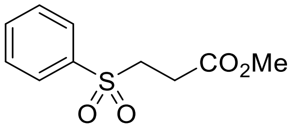

Methyl 3-(phenylsulfonyl)propanoate (5m)

Prepared according to **general procedure A**: iodobenzene (56 µL, 0.50 mmol, 1.0 equiv.), SMOPS (104 mg, 0.60 mmol, 1.2 equiv.), CuI (9.5 mg, 0.05 mmol, 10 mol%) and **L3** (5 µL, 0.05 mmol, 10 mol%) were dissolved in DMSO (2.6 mL, 0.13 M). The reaction mixture was stirred at 50 °C for 24 h. After aqueous work up, the crude product was purified by flash column chromatography on silica gel (30% EtOAc in Petrol) to give the product as a colourless oil (74.2 mg, 65%).

^{1}H NMR (400 MHz, $CDCl_3$) δ 7.94 – 7.86 (m, 2H), 7.71 – 7.62 (m, 1H), 7.58 (d, *J* = 7.8 Hz, 1H), 3.62 (s, 3H), 3.42 (t, *J* = 7.6 Hz, 1H), 2.74 (t, *J* = 7.6, 2H); **^{13}C NMR** (101 MHz, $CDCl_3$) δ 170.5, 138.6, 134.2, 129.6, 128.3, 52.4, 51.6, 27.8; **HRMS** (ESI) *m/z*: $[M+Na]^+$ calcd for $C_{10}H_{12}O_4SNa^+$ 251.0349, found 251.0353; **R$_f$** = 0.32 (30% EtOAc in Petrol). Data is consistent with literature.[7]

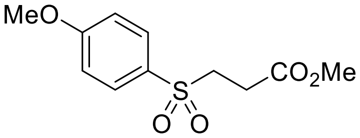

Methyl 3-((4-methoxyphenyl)sulfonyl)propanoate (5n)

Prepared according to **general procedure A**: 4-iodoanisole (46.8 mg, 0.20 mmol, 1.0 equiv.), SMOPS (41.8 mg, 0.24 mmol, 1.2 equiv.), CuI (3.8 mg, 0.02 mmol, 10 mol%) and **L3** (2 µL, 0.02 mmol, 10 mol%) were dissolved in DMSO (1.5 mL, 0.13 M). The reaction mixture was stirred at 50 °C for 24 h. After aqueous work up, the crude product was purified by flash column chromatography on silica gel (50% EtOAc in Petrol) to give the product as a white solid (26.3 mg, 51%).

^{1}H NMR (400 MHz, $CDCl_3$) δ 7.83 (d, *J* = 8.9 Hz, 2H), 7.03 (d, *J* = 8.9 Hz, 2H), 3.89 (s, 3H), 3.65 (s, 3H), 3.40 (t, *J* = 7.1 Hz, 2H), 2.75 (t, *J* = 7.1 Hz, 2H); **^{13}C NMR** (101 MHz, $CDCl_3$) δ 170.7, 164.1, 130.5, 130.1, 114.7, 55.9, 52.5, 51.9, 27.9; **HRMS** (ESI) *m/z*: $[M+Na]^+$ calcd for $C_{11}H_{14}O_5SNa^+$ 281.0454, found 281.0464; **R_f** = 0.14 (30% EtOAc in Petrol). Data is consistent with literature.[8]

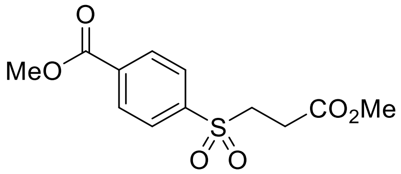

Methyl 4-((3-methoxy-3-oxopropyl)sulfonyl)benzoate (5o)

Prepared according to **general procedure A**: methyl 4-iodobenzoate (52.4 mg, 0.20 mmol, 1.0 equiv.), SMOPS (41.8 mg, 0.24 mmol, 1.2 equiv.), CuI (3.8 mg, 0.02 mmol, 10 mol%) and **L3** (2 µL, 0.02 mmol, 10 mol%) were dissolved in DMSO (1.5 mL, 0.13 M). The reaction mixture was stirred at 50 °C for 24 h. After aqueous work up, the crude product was purified by flash column chromatography on silica gel (50% EtOAc in Petrol) to give the product as a white solid (40.6 mg, 71%).

^{1}H NMR (400 MHz, $CDCl_3$) δ 8.24 (d, *J* = 8.7 Hz, 2H), 8.00 (d, *J* = 8.7 Hz, 2H), 3.98 (s, 3H), 3.65 (s, 3H), 3.46 (app. dd, *J* = 8.1, 7.1 Hz, 2H), 2.78 (app. dd, *J* = 8.1, 7.1 Hz, 2H); **^{13}C NMR** (101 MHz, $CDCl_3$) δ 170.4, 165.5, 142.4, 135.3, 130.7, 128.5, 53.0, 52.6, 51.5, 27.6; **HRMS** (ESI) *m/z*: $[M+Na]^+$ calcd for $C_{12}H_{14}O_6SNa^+$ 309.0403, found 309.0411; **IR** $_{vmax}$ (neat)/cm^{-1} 1734, 1438, 1289, 1217, 1148, 1116, 1017, 750, 711; **mp** 120 – 122 °C; **R_f** = 0.46 (50% EtOAc in Petrol).

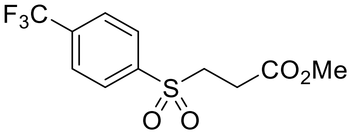

Methyl 3-((4-(trifluoromethyl)phenyl)sulfonyl)propanoate (5p)

Prepared according to **general procedure A**: 1-iodo-4-(trifluoromethyl)benzene (29 µL, 0.20 mmol, 1.0 equiv.), SMOPS (41.8 mg, 0.24 mmol, 1.2 equiv.), CuI (3.8 mg, 0.02 mmol, 10 mol%) and **L3** (2 µL, 0.02 mmol, 10 mol%) were dissolved in DMSO (1.5 mL, 0.13 M). The reaction mixture was stirred at 50 °C for 24 h. After aqueous work up, the crude product was purified by flash column chromatography on silica gel (40% EtOAc in Petrol) to give the product as a white solid (41.5 mg, 70%).

^{1}H NMR (400 MHz, CDCl3) δ 8.07 (d, *J* = 8.2 Hz, 2H), 7.86 (d, *J* = 8.2 Hz, 2H), 3.64 (s, 3H), 3.47 (app. dd, *J* = 8.1, 7.1 Hz, 2H), 2.78 (app. dd, *J* = 8.0, 7.1 Hz, 2H); **^{13}C NMR** (151 MHz, $CDCl_3$) δ 170.3, 142.2, 135.9 (q, $^2J_{C\text{-}F}$ = 33.3 Hz), 129.1, 126.7 (q, $^3J_{C\text{-}F}$ = 3.6 Hz), 123.2 (q, $^1J_{C\text{-}F}$ = 273.0 Hz), 52.6, 51.6, 27.6; **^{19}F NMR** (377 MHz, $CDCl_3$) δ -63.2; **HRMS** (ESI) *m/z*: $[M+Na]^+$ calcd for $C_{11}H_{11}F_3O_4SNa^+$ 319.0222, found 319.0237; **R_f** = 0.31 (40% EtOAc in Petrol). Data is consistent with literature.[9]

General procedure B: Cu-catalysed sulfone formation from bromides

To an oven-dried microwave vial (10 mL) aryl bromide (if solid) (0.20 mmol, 1.0 equiv.), STOPS (51.9 mg, 0.24 mmol, 1.2 equiv.) and CuI (3.8 mg, 0.02 mmol, 10 mol%) and were added. The vial was capped and vacuum/N_2 purged (× 3), before adding anhydrous, degassed DMSO (1.5 mL, 0.13 M), aryl bromide (if liquid) (0.20 mmol, 1.0 equiv.) and **L4** (3 µL, 0.02 mmol, 10 mol%). The vial was further sealed with parafilm, then stirred at 70 °C in an aluminium heating block for 24 h. The reaction mixture was then

cooled to r.t., 10 mL of water was added, and the product was extracted from the aqueous with EtOAc (3 × 15 mL). The combined organic extracts were washed with brine, dried over Na_2SO_4 and concentrated *in vacuo*. The crude product was purified by flash column chromatography (EtOAc in Petrol) to afford the desired aryl sulfone product.

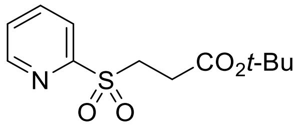

tert-Butyl 3-(pyridin-2-ylsulfonyl)propanoate (7a)

Prepared according to **general procedure B**: 2-bromopyridine (19 µL, 0.20 mmol, 1.0 equiv.), STOPS (51.9 mg, 0.24 mmol, 1.2 equiv.), CuI (3.8 mg, 0.02 mmol, 10 mol%) and **L4** (3 µL, 0.02 mmol, 10 mol%) were dissolved in DMSO (1.5 mL, 0.13 M). The reaction mixture was stirred at 70 °C for 24 h. After aqueous work up, the crude product was purified by flash column chromatography on silica gel (30-40% EtOAc in Petrol) to give the product as a pale-yellow oil (37.8 mg, 70%).

Also prepared from 2-iodopyridine: 2-iodopyridine (21 µL, 0.20 mmol, 1.0 equiv.), STOPS (51.9 mg, 0.24 mmol, 1.2 equiv.), CuI (3.8 mg, 0.02 mmol, 10 mol%) and **L3** (2 µL, 0.02 mmol, 10 mol%) were dissolved in DMSO (1.5 mL, 0.13 M). The reaction mixture was stirred at 50 °C for 24 h. After aqueous work up, the crude product was purified by flash column chromatography on silica gel (50% EtOAc in Petrol) to give the product as a pale-yellow oil (46.9 mg, 86%).

^{1}H NMR (400 MHz, $CDCl_3$) δ 8.76 (ddd, *J* = 4.8, 1.7, 0.9 Hz, 1H), 8.09 (dt, *J* = 7.9, 1.1 Hz, 1H), 7.97 (td, *J* = 7.7, 1.7 Hz, 1H), 7.57 (ddd, *J* = 7.7, 4.8, 1.1 Hz, 1H), 3.72 – 3.64 (m, 2H), 2.78 – 2.69 (m, 2H), 1.43 (s, 9H); **^{13}C NMR** (101 MHz, $CDCl_3$) δ 169.3, 157.2, 150.5, 138.4, 127.7, 122.3, 82.0, 47.9, 28.8, 28.1; **HRMS** (ESI) *m/z*: $[M+Na]^+$ calcd for $C_{12}H_{17}NO_4SNa^+$ 294.0771, found 294.0777; **IR** $_{vmax}$ (neat)/cm^{-1} 1730, 1579, 1428, 1369, 1156, 1112, 992, 844, 781, 747, 699; **R$_f$** = 0.32 (50% EtOAc in Petrol).

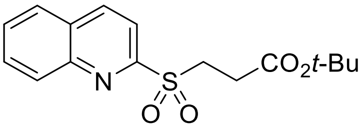

tert-Butyl 3-(quinolin-2-ylsulfonyl)propanoate (7b)

Prepared according to **general procedure B**: 2-bromoquinoline (41.6 mg, 0.20 mmol, 1.0 equiv.), STOPS (51.9 mg, 0.24 mmol, 1.2 equiv.), CuI (3.8 mg, 0.02 mmol, 10 mol%) and **L4** (3 µL, 0.02 mmol, 10 mol%) were dissolved in DMSO (1.5 mL, 0.13 M). The reaction mixture was stirred at 70 °C for 24 h. After aqueous work up, the crude product was purified by flash column chromatography on silica gel (20% EtOAc in Petrol) to give the product as an off-white solid (53.8 mg, 84%).

1H NMR (400 MHz, $CDCl_3$) δ 8.44 (dd, *J* = 8.5, 0.9 Hz, 1H), 8.23 (dq, *J* = 8.5, 0.9 Hz, 1H), 8.12 (d, *J* = 8.6 Hz, 1H), 7.94 (dd, *J* = 8.2, 1.4 Hz, 1H), 7.86 (ddd, *J* = 8.5, 6.9, 1.5 Hz, 1H), 7.73 (ddd, *J* = 8.2, 6.9, 1.2 Hz, 1H), 3.84 (t, *J* = 7.9 Hz, 2H), 2.81 (t, *J* = 7.9 Hz, 2H), 1.41 (s, 9H); **^{13}C NMR** (101 MHz, $CDCl_3$) δ 169.4, 156.7, 147.3, 139.1, 131.4, 130.4, 129.5, 129.4, 128.0, 117.3, 82.0, 47.8, 28.8, 28.1; **HRMS** (ESI) *m/z*: $[M+Na]^+$ calcd for $C_{16}H_{19}NO_4SNa^+$ 344.0927, found 344.0935; **IR** $_{vmax}$ (neat)/cm^{-1} 1730, 1581, 1499, 1425, 1368, 1316, 1252, 1163, 1124, 1096, 834, 756; **mp** 70 – 74 °C; **R$_f$** = 0.41 (30% EtOAc in Petrol).

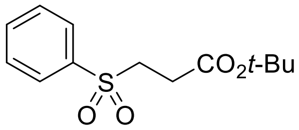

tert-Butyl 3-(phenylsulfonyl)propanoate (7c)

Prepared according to **general procedure B**: bromobenzene (21 µL, 0.20 mmol, 1.0 equiv.), STOPS (51.9 mg, 0.24 mmol, 1.2 equiv.), CuI (3.8 mg, 0.02 mmol, 10 mol%) and **L4** (3 µL, 0.02 mmol, 10 mol%) were dissolved in DMSO (1.5 mL, 0.13 M). The reaction mixture was stirred at 70 °C for 24 h. After aqueous work up, the crude product was purified by flash column chromatography on silica gel (10-20% EtOAc in Petrol) to give the product as a pale-yellow oil (32.4 mg, 60%).

^{1}H NMR (400 MHz, $CDCl_3$) δ 7.94 – 7.87 (m, 2H), 7.70 – 7.61 (m, 1H), 7.61 – 7.52 (m, 2H), 3.38 (t, *J* = 8.0 Hz, 2H), 2.65 (t, *J* = 8.0 Hz, 1H), 1.39 (s, 9H); **^{13}C NMR** (101 MHz, CDCl3) δ 169.2, 138.8, 134.0, 129.5, 129.5, 128.3, 82.0, 51.7, 29.0, 28.1; **HRMS** (ESI) *m/z*: $[M+Na]^+$ calcd for $C_{13}H_{18}O_4SNa^+$ 293.0818, found 293.0827; **R_f** = 0.23 (10% EtOAc in Petrol). Data is consistent with literature.[10]

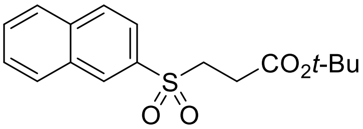

tert-Butyl 3-(naphthalen-2-ylsulfonyl)propanoate (7d)

Prepared according to **general procedure B**: 2-bromonaphthalene (41.1 mg, 0.20 mmol, 1.0 equiv.), STOPS (51.9 mg, 0.24 mmol, 1.2 equiv.), CuI (3.8 mg, 0.02 mmol, 10 mol%) and **L4** (3 µL, 0.02 mmol, 10 mol%) were dissolved in DMSO (1.5 mL, 0.13 M). The reaction mixture was stirred at 70 °C for 24 h. After aqueous work up, the crude product was purified by flash column chromatography on silica gel (20% EtOAc in Petrol) to give the product as an off-white solid (37.8 mg, 59%).

^{1}H NMR (400 MHz, $CDCl_3$) δ 8.49 – 8.48 (m, 1H), 8.04 – 7.99 (m, 2H), 7.96 – 7.93 (m, 1H), 7.87 (dd, *J* = 8.7, 1.9 Hz, 1H), 7.69 (ddd, *J* = 8.2, 6.9, 1.5 Hz, 1H), 7.64 (ddd, *J* = 8.2, 6.8, 1.5 Hz, 1H), 3.49 – 3.44 (m, 2H), 2.71 – 2.67 (m, 2H), 1.35 (s, 9H); **^{13}C NMR** (101 MHz, $CDCl_3$) δ 169.2, 135.6, 135.6, 132.3, 130.2, 129.9, 129.6, 129.6, 128.1, 127.9, 122.9, 82.0, 51.8, 29.2, 28.0; **HRMS** (ESI) *m/z*: $[M+Na]^+$ calcd for $C_{17}H_{20}O_4SNa^+$ 343.0975, found 343.0975; **IR** 1733, 1376, 1317, 1254, 1147, 1120, 1071, 823, 755, 695; **mp** 52 – 54 °C; **R_f** = 0.43 (20% EtOAc in Petrol).

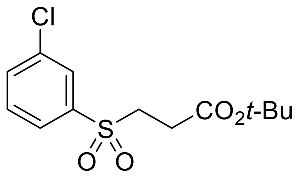

tert-Butyl 3-((3-chlorophenyl)sulfonyl)propanoate (7e)

Prepared according to **general procedure B**: 1-bromo-3-chlorobenzene (24 µL, 0.20 mmol, 1.0 equiv.), STOPS (51.9 mg, 0.24 mmol, 1.2 equiv.), CuI (3.8 mg, 0.02 mmol, 10 mol%) and **L4** (3 µL, 0.02 mmol, 10 mol%) were dissolved in DMSO (1.5 mL, 0.13 M). The reaction mixture was stirred at 70 °C for 24 h. After aqueous work up, the crude product was purified by flash column chromatography on silica gel (5% EtOAc in Petrol) to give the product as a colourless oil (38.0 mg, 62%).

^{1}H NMR (400 MHz, $CDCl_3$) δ 7.90 (t, *J* = 1.8 Hz, 1H), 7.80 (ddd, *J* = 7.9, 1.8, 1.1 Hz, 1H), 7.64 (ddd, *J* = 7.9, 2.1, 1.1 Hz, 1H), 7.53 (t, *J* = 7.9 Hz, 1H), 3.43 – 3.37 (m, 2H), 2.69 – 2.64 (m, 2H), 1.41 (s, 9H); **^{13}C NMR** (101 MHz, $CDCl_3$) δ 169.0, 140.6, 135.8, 134.3, 130.9, 128.4, 126.5, 82.2, 51.8, 28.9, 28.1; **HRMS** (ESI) *m/z*: $[M+Na]^+$ calcd for $C_{13}H_{17}ClO_4SNa^+$ 327.0428, found 327.0434; **IR** $_{vmax}$ (neat)/cm^{-1} 1735, 1582, 1423, 1326, 1297, 1207, 991, 848, 762, 612; **R$_f$** = 0.31 (10% EtOAc in Petrol).

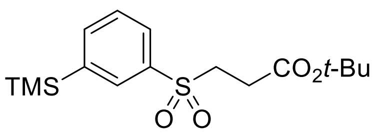

tert-Butyl 3-((3-(trimethylsilyl)phenyl)sulfonyl)propanoate (7f)

Prepared according to **general procedure B**: 1-bromo-3-(trimethylsilyl)benzene (37 µL, 0.20 mmol, 1.0 equiv.), STOPS (51.9 mg, 0.24 mmol, 1.2 equiv.), CuI (3.8 mg, 0.02 mmol, 10 mol%) and **L4** (3 µL, 0.02 mmol, 10 mol%) were dissolved in DMSO (1.5 mL, 0.13 M). The reaction mixture was stirred at 70 °C for 24 h. After aqueous work up, the crude product was purified by flash column chromatography on silica gel (5% EtOAc in Petrol) to give the product as a colourless oil (39.0 mg, 57%).

^{1}H NMR (400 MHz, $CDCl_3$) δ 8.03 – 7.99 (m, 1H), 7.87 (ddd, *J* = 7.8, 2.0, 1.2 Hz, 1H), 7.79 (dt, *J* = 7.3, 1.2 Hz, 1H), 7.54 (td, *J* = 7.6, 0.6 Hz, 1H), 3.38 (t, *J* = 7.7 Hz, 2H), 2.68 (t, *J* = 7.7 Hz, 2H), 1.40 (s, 9H), 0.31 (s, 9H); **^{13}C NMR** (101 MHz, $CDCl_3$) δ 169.3, 143.3, 138.9, 138.2, 132.5, 128.7, 128.5, 82.0, 51.8, 28.9, 28.1, -1.2; **HRMS** (ESI) *m/z*: $[M+Na]^+$ calcd for $C_{16}H_{26}O_4SSiNa^+$ 365.1213, found 365.1228; **IR** $_{vmax}$ (neat)/cm^{-1} 1732, 1369, 1322, 1252, 1157, 1121, 843, 755; **R$_f$** = 0.46 (10% EtOAc in Petrol).

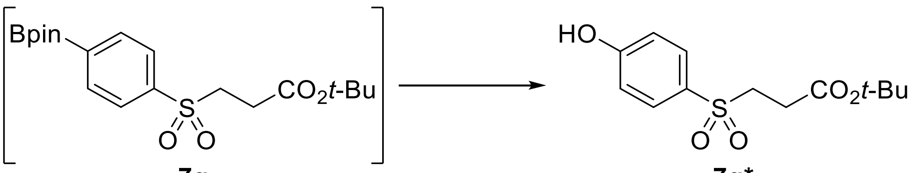

7g **7g***

tert-Butyl 3-((4-hydroxyphenyl)sulfonyl)propanoate (7g*)

Prepared according to **modified general procedure B**: 2-(4-Bromophenyl)-4,4,5,5-tetramethyl-1,3,2-dioxaborolane (56.6 mg, 0.20 mmol, 1.0 equiv.), STOPS (51.9 mg, 0.24 mmol, 1.2 equiv.), CuI (7.6 mg, 0.04 mmol, 20 mol%) and **L4** (6 µL, 0.04 mmol, 20 mol%) were dissolved in DMSO (1.5 mL, 0.13 M). The reaction mixture was stirred at 70 °C for 24 h. After aqueous work up, the crude product (**7g**) was used directly in the next step.

The crude mixture was dissolved in THF (1 mL), then H_2O (1 mL) and $NaBO_3·4H_2O$ (461.6 mg, 3.00 mmol, 15 equiv.) were added. The reaction mixture was stirred at r.t. for 1 h then quenched with aqueous sat. NH_4Cl. The product was extracted from the aqueous with EtOAc (3 × 15 mL). The combined organic extracts were washed with brine, dried over Na_2SO_4 and concentrated *in vacuo*. The crude product was purified by flash column chromatography on silica gel (50% EtOAc in Petrol) to give the product as a white solid (32.9 mg, 57%).

^{1}H NMR (400 MHz, $CDCl_3$) δ 7.78 (d, *J* = 8.9 Hz, 2H), 6.96 (d, *J* = 8.9 Hz, 2H), 5.98 (br s, 1H) 3.36 (t, *J* = 8.1 Hz, 2H), 2.65 (t, *J* = 8.1 Hz, 2H), 1.41 (s, 9H); **^{13}C NMR** (151 MHz, $CDCl_3$) δ 169.5, 160.7, 130.8, 130.4, 116.3, 82.1, 52.0, 29.3, 28.1; **HRMS** (ESI) *m/z*: $[M+Na]^+$ calcd for $C_{13}H_{18}O_5SNa^+$ 309.0767, found

309.0763; **IR** $_{vmax}$ (neat)/cm^{-1} 3264, 1691, 1584, 1441, 1386, 1312, 1209, 1088, 842, 777, 691; **mp** 150 – 154 °C; $\mathbf{R_f}$ = 0.39 (50% EtOAc in Petrol).

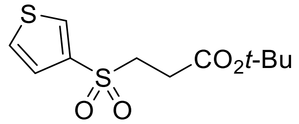

***tert*-Butyl 3-(thiophen-3-ylsulfonyl)propanoate (7h)**

Prepared according to **general procedure B**: 3-bromothiophene (19 µL, 0.20 mmol, 1.0 equiv.), STOPS (51.9 mg, 0.24 mmol, 1.2 equiv.), CuI (3.8 mg, 0.02 mmol, 10 mol%) and **L4** (3 µL, 0.02 mmol, 10 mol%) were dissolved in DMSO (1.5 mL, 0.13 M). The reaction mixture was stirred at 70 °C for 24 h. After aqueous work up, the crude product was purified by flash column chromatography on silica gel (10-20% EtOAc in Petrol) to give the product as an off-white solid (40.4 mg, 73%).

^{1}H NMR (400 MHz, $CDCl_3$) δ 8.09 (dd, *J* = 3.1, 1.3 Hz, 1H), 7.48 (dd, *J* = 5.1, 3.1 Hz, 1H), 7.40 (dd, *J* = 5.1, 1.3 Hz, 1H), 3.42 (t, *J* = 7.5 Hz, 1H), 2.69 (t, *J* = 7.5 Hz, 1H), 1.42 (s, 9H); **^{13}C NMR** (101 MHz, $CDCl_3$) δ 169.2, 139.4, 133.2, 128.7, 126.2, 82.1, 52.0, 29.1, 28.1; **HRMS** (ESI) *m/z*: $[M+Na]^+$ calcd for $C_{11}H_{16}O_4S_2Na^+$ 299.0382, found 299.0388; **IR** $_{vmax}$ (neat)/cm^{-1} 1732, 1370, 1319, 1257, 1153, 1100, 826, 738, 637; **mp** 60 – 62 °C; $\mathbf{R_f}$ = 0.22 (20% EtOAc in Petrol).

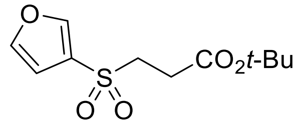

***tert*-Butyl 3-(furan-3-ylsulfonyl)propanoate (7i)**

Prepared according to **general procedure B**: 3-bromofuran (18 µL, 0.20 mmol, 1.0 equiv.), STOPS (51.9 mg, 0.24 mmol, 1.2 equiv.), CuI (3.8 mg, 0.02 mmol, 10 mol%) and **L4** (3 µL, 0.02 mmol, 10 mol%) were dissolved in DMSO (1.5 mL, 0.13 M). The reaction mixture was stirred at 70 °C for 24 h. After aqueous work up, the crude product was purified by flash column chromatography on silica gel (10-20% EtOAc in Petrol) to give the product as an off-white solid (52.1 mg, 71%).

^{1}H NMR (400 MHz, $CDCl_3$) δ 7.99 (dd, *J* = 1.8, 0.9 Hz, 1H), 7.53 (t, *J* = 1.8 Hz, 1H), 6.70 (dd, *J* = 1.8, 0.9 Hz, 1H), 3.47 – 3.36 (m, 2H), 2.79 – 2.62 (m, 2H), 1.42 (s, 9H); **^{13}C NMR** (101 MHz, $CDCl_3$) δ 169.1, 147.4, 145.2, 126.7, 109.0, 82.1, 52.1, 29.1, 28.1; **HRMS** (ESI) *m/z*: $[M+Na]^+$ calcd for $C_{11}H_{16}O_5SNa^+$ 283.0611, found 283.0621; **IR** $_{vmax}$ (neat)/cm^{-1} 1699, 1421, 1311, 1208, 1122, 1012, 874, 743, 631; **mp** 96 – 98 °C; $\mathbf{R_f}$ = 0.37 (20% EtOAc in Petrol).

5.3. Functionalisation of masked sulfinates

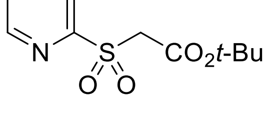

***tert*-Butyl 2-(pyridin-2-ylsulfonyl)acetate (8a)**

Methyl 3-(pyridin-2-ylsulfonyl)propanoate (**5a**) (45.9 mg, 0.2 mmol, 1.0 equiv.) *OR tert*-butyl 3-(pyridin-2-ylsulfonyl)propanoate (**7a**) (54.2 mg, 0.2 mmol, 1.0 equiv.) was dissolved in DMSO (1 mL, 0.2 M) and NaOMe (30% w/w in MeOH, 37 µL, 0.2 mmol, 1.0 equiv.) was added. The reaction mixture

was stirred at r.t. for 10 min, until entire consumption of the sulfone starting material was confirmed by TLC. *tert*-Butyl bromoacetate (59 µL, 0.4 mmol, 2.0 equiv.) was then added, and the resulting mixture was stirred for 1 h at r.t. until the reaction was deemed complete by TLC. 10 mL of water was added, and the product was extracted from the aqueous with EtOAc (3 × 15 mL). The combined organic extracts were washed with brine, dried over Na_2SO_4 and concentrated *in vacuo*. The crude product was purified by flash column chromatography (20-30% EtOAc in Petrol) to give the product as an off-white solid (37.0 mg, 73%) when methyl 3-(pyridin-2-ylsulfonyl)propanoate (**5a**) is used *OR* (39.5 mg, 78%) when *tert*-butyl 3-(pyridin-2-ylsulfonyl)propanoate (**7a**) is used.

^{1}H NMR (400 MHz, $CDCl_3$) δ 8.76 (ddd, *J* = 4.7, 1.7, 1.0 Hz, 1H), 8.11 (dt, *J* = 7.9, 1.0 Hz, 1H), 7.99 (td, *J* = 7.9, 1.7 Hz, 1H), 7.57 (ddd, *J* = 7.9, 4.7, 1.0 Hz, 1H), 4.41 (s, 2H), 1.31 (s, 9H); **^{13}C NMR** (101 MHz, $CDCl_3$) δ 161.4, 157.1, 150.3, 138.2, 127.6, 122.4, 83.7, 57.3, 27.8; **HRMS** (ESI) *m/z*: $[M+Na]^+$ calcd for $C_{11}H_{15}NO_4SNa^+$ 280.0614, found 280.0628; **R$_f$** = 0.30 (30% EtOAc in Petrol). Data is consistent with literature.[11]

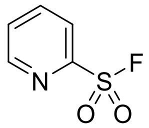

Pyridine-2-sulfonyl fluoride, PyFluor (8b)

Methyl 3-(pyridin-2-ylsulfonyl)propanoate (**5a**) (45.9 mg, 0.2 mmol, 1.0 equiv.) *OR tert*-butyl 3-(pyridin-2-ylsulfonyl)propanoate (**7a**) (54.2 mg, 0.2 mmol, 1.0 equiv.) was dissolved in DMSO (1 mL, 0.2 M) and NaOMe (30% w/w in MeOH, 37 µL, 0.2 mmol, 1.0 equiv.) was added. The reaction mixture was stirred at r.t. for 10 min, until entire consumption of the sulfone starting material was confirmed by TLC. NFSI (94.6 mg, 0.3 mmol, 1.5 equiv.) was then added, and the resulting mixture was stirred for 1 h at r.t. until the reaction was deemed complete by TLC. 10 mL of water was added, and the product was extracted from the aqueous with EtOAc (3 × 15 mL). The combined organic extracts were washed with brine, dried over Na_2SO_4 and concentrated *in vacuo*. The crude product was purified by flash column chromatography (20% EtOAc in Petrol) to give the product as an off-white solid (37.0 mg, 72%) when methyl 3-(pyridin-2-ylsulfonyl)propanoate (**5a**) is used *OR* (36.0 mg, 71%) when *tert*-butyl 3-(pyridin-2-ylsulfonyl)propanoate (**7a**) is used.

^{1}H NMR (400 MHz, $CDCl_3$) δ 8.86 (ddd, *J* = 4.7, 1.7, 0.9 Hz, 1H), 8.14 (dq, *J* = 7.9, 0.9 Hz, 1H), 8.06 (tt, *J* = 7.8, 1.5 Hz, 1H), 7.71 (ddd, *J* = 7.7, 4.7, 1.2 Hz, 1H); **^{13}C NMR** (151 MHz, $CDCl_3$) δ 151.5 (d, $^2J_{C\text{-}F}$ = 30.5 Hz), 151.2, 138.8, 129.3, 124.3 (d, $^3J_{C\text{-}F}$ = 2.2 Hz); **^{19}F NMR** (377 MHz, $CDCl_3$) δ 55.8; **HRMS** (ESI) *m/z*: $[M+Na]^+$ calcd for $C_5H_4FNO_2SNa^+$ 183.9839, found 183.9839; **R$_f$** = 0.53 (30% EtOAc in Petrol). Data is consistent with literature.[12]

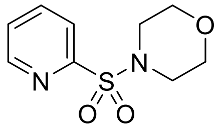

4-(Pyridin-2-ylsulfonyl)morpholine (8c)

Methyl 3-(pyridin-2-ylsulfonyl)propanoate (**5a**) (45.9 mg, 0.2 mmol, 1.0 equiv.) *OR tert*-butyl 3-(pyridin-2-ylsulfonyl)propanoate (**7a**) (54.2 mg, 0.2 mmol, 1.0 equiv.) was dissolved in DMSO (1 mL, 0.2 M) and NaOMe (30% w/w in MeOH, 37 µL, 0.2 mmol, 1.0 equiv.) was added. The reaction mixture

was stirred at r.t. for 10 min, until entire consumption of the sulfone starting material was confirmed by TLC. *N*-chlorosuccinimide (53.4 mg, 0.4 mmol, 2.0 equiv.) and morpholine (35 µL, 0.3 mmol, 1.5 equiv.) were then added, and the resulting mixture was stirred for 1 h at r.t. until the reaction was deemed complete by TLC. 10 mL of water was added, and the product was extracted from the aqueous with EtOAc (3 × 15 mL). The combined organic extracts were washed with brine, dried over Na_2SO_4 and concentrated *in vacuo*. The crude product was purified by flash column chromatography (50% EtOAc in Petrol) to give the product as a white solid (33.3 mg, 72%) when methyl 3-(pyridin-2-ylsulfonyl)propanoate (**5a**) is used *OR* (31.0 mg, 67%) when *tert*-butyl 3-(pyridin-2-ylsulfonyl)propanoate (**7a**) is used.

^{1}H NMR (400 MHz, $CDCl_3$) δ 8.73 (dt, *J* = 4.6, 1.4 Hz, 1H), 7.99 – 7.88 (m, 2H), 7.52 (ddd, *J* = 6.5, 4.6, 2.4 Hz, 1H), 3.78 – 3.71 (m, 4H), 3.37 – 3.30 (m, 4H); **^{13}C NMR** (101 MHz, $CDCl_3$) δ 156.2, 150.2, 138.1, 126.9, 123.3, 66.6, 46.7; **HRMS** (ESI) *m/z*: $[M+Na]^+$ calcd for $C_9H_{12}N_2O_3SNa^+$ 251.0461, found 251.0462; **R_f** = 0.15 (30% EtOAc in Petrol). Data is consistent with literature.[13]

6. NMR Spectra

Sodium 3-methoxy-3-oxopropane-1-sulfinate, SMOPS (2)

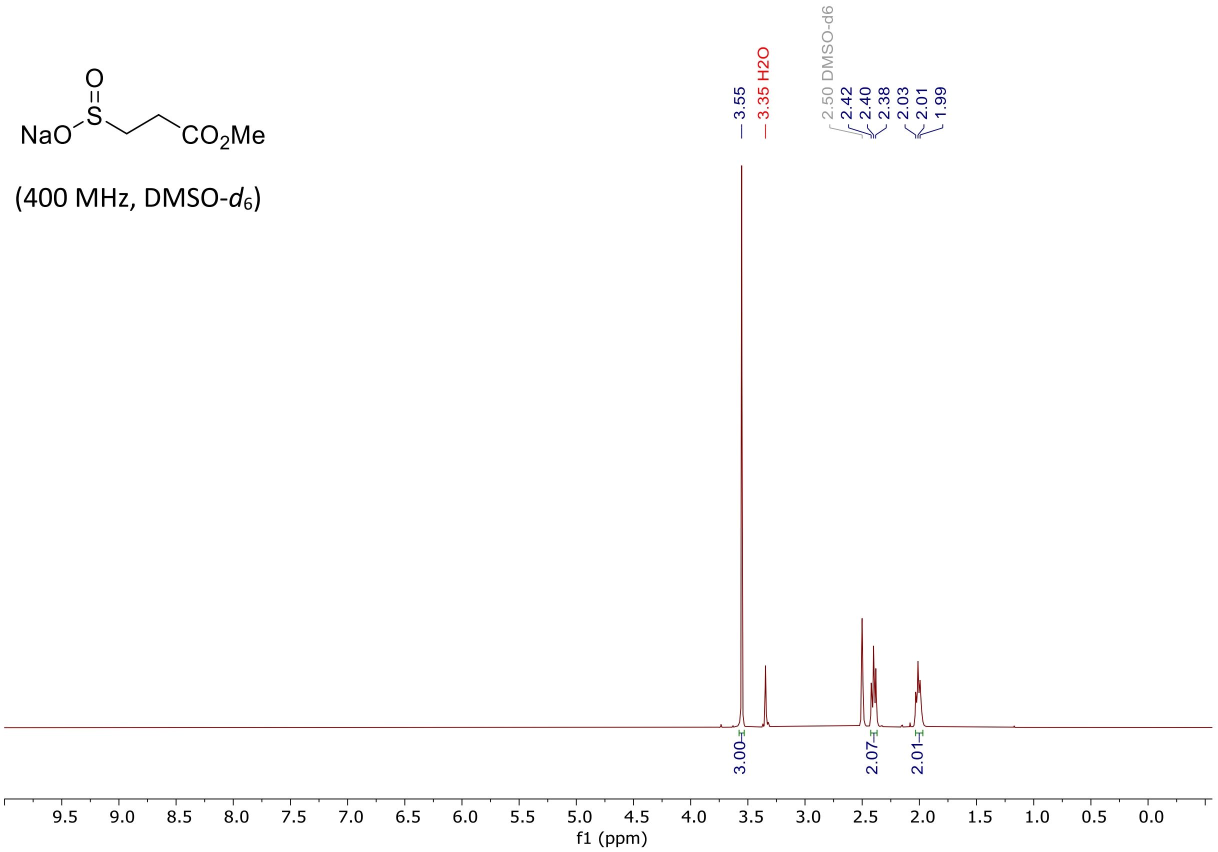

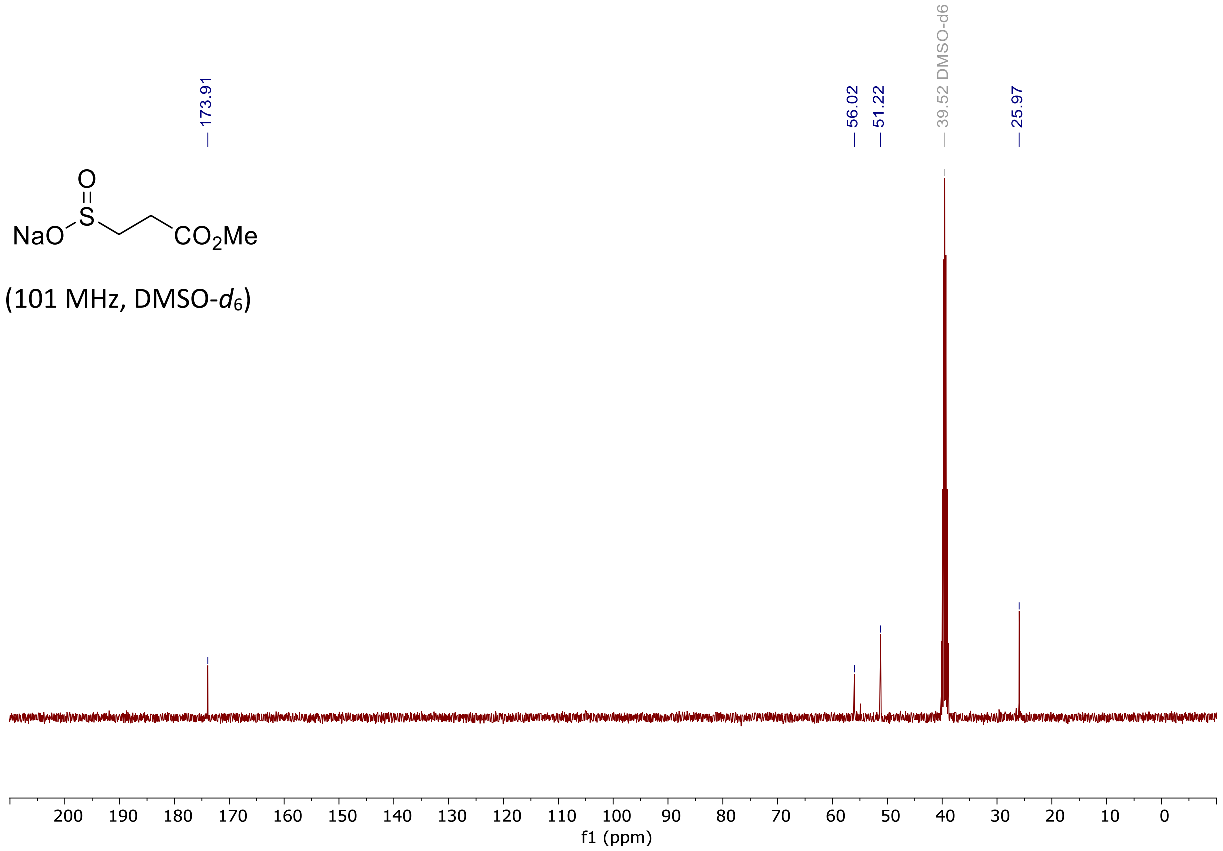

Sodium 3-(*tert*-butoxy)-3-oxopropane-1-sulfinate, STOPS (3)

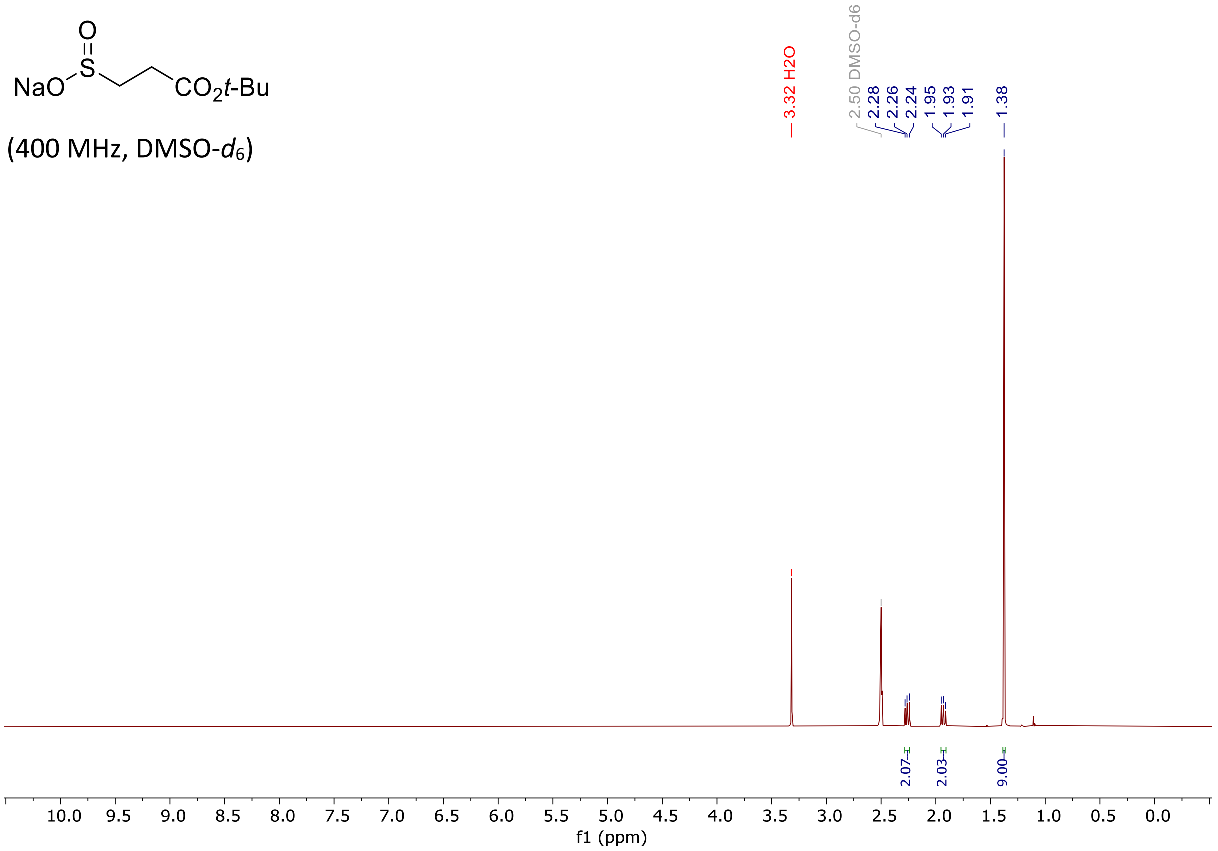

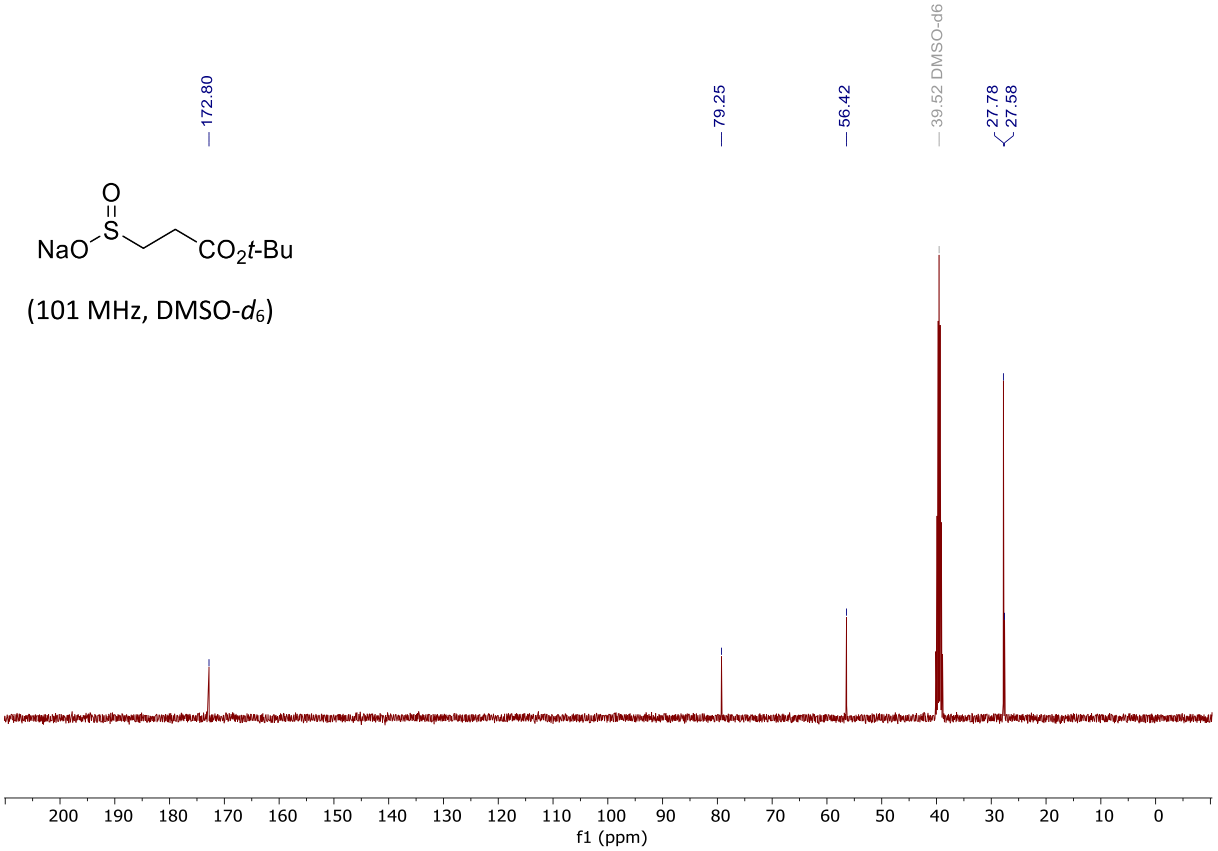

Methyl 3-(pyridin-2-ylsulfonyl)propanoate (5a)

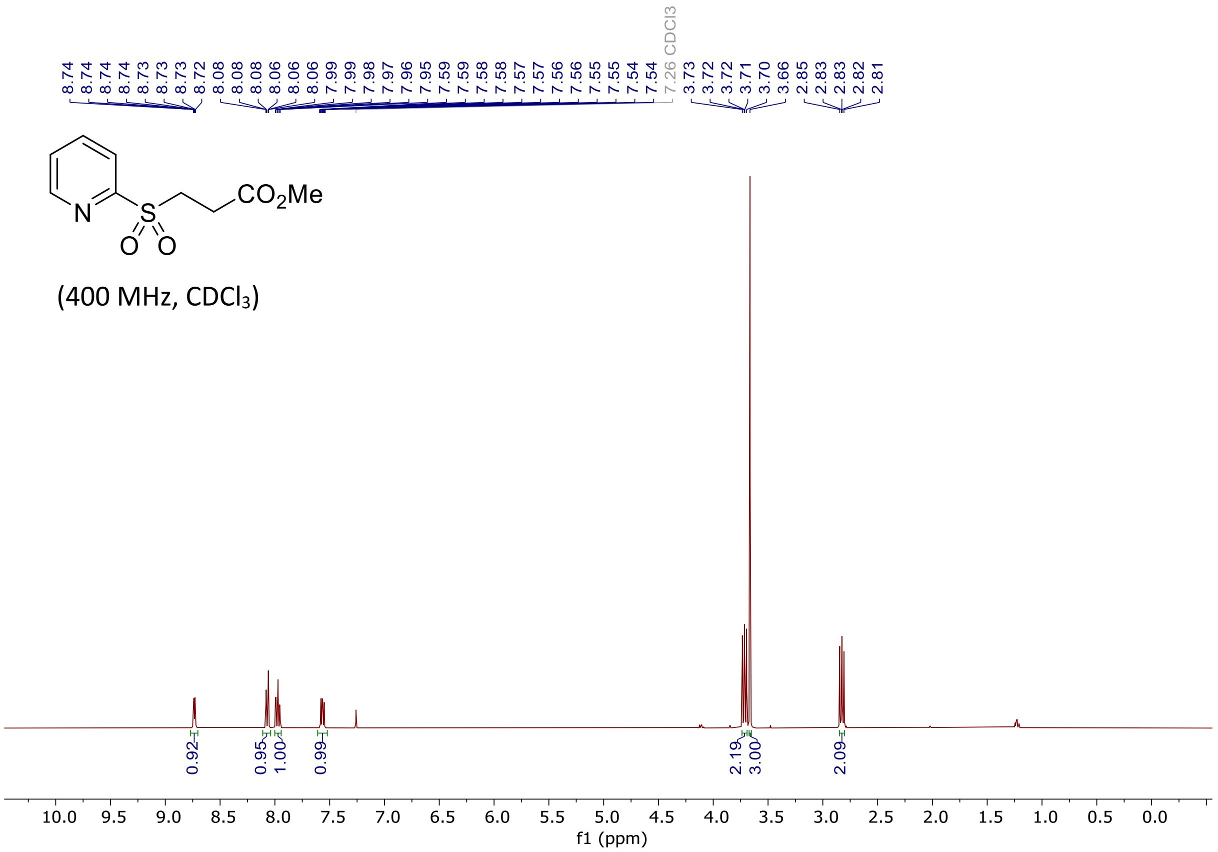

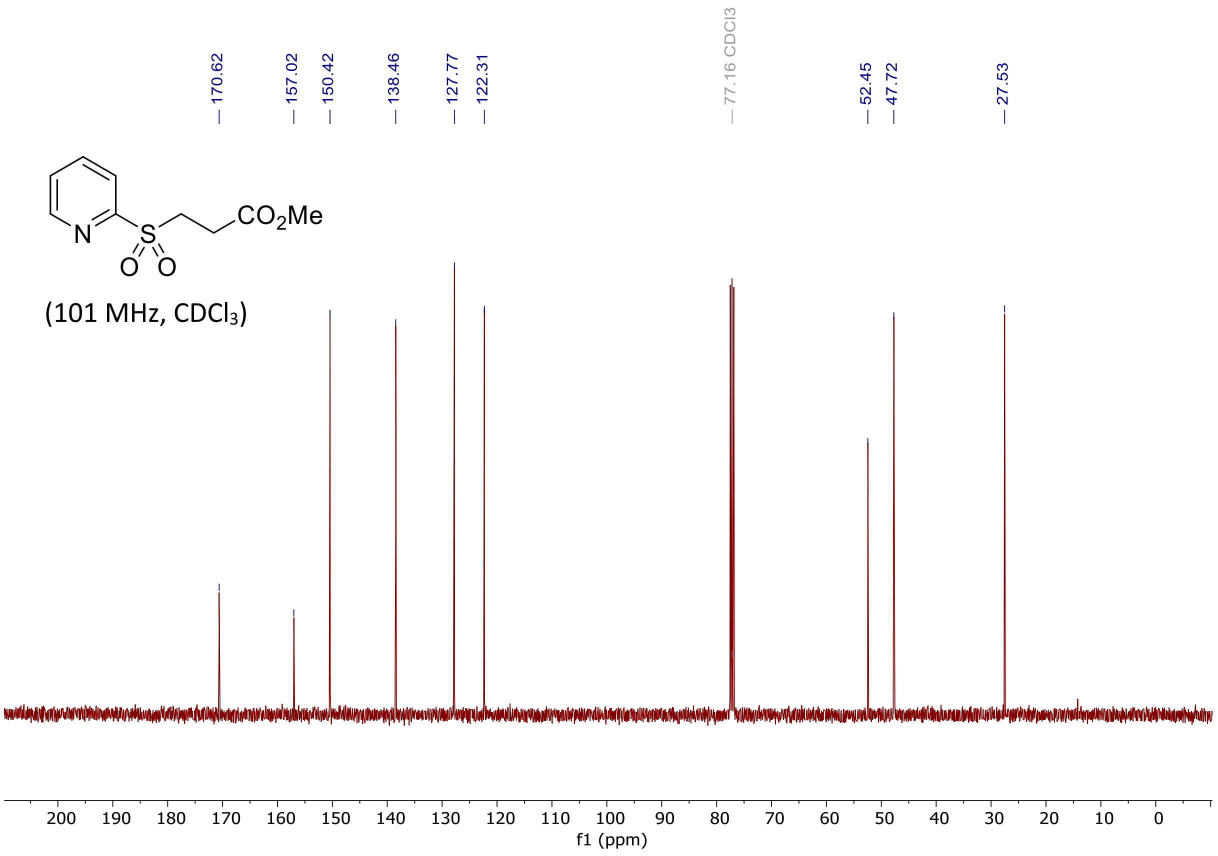

Methyl 3-((6-(trifluoromethyl)pyridin-2-yl)sulfonyl)propanoate (5b)

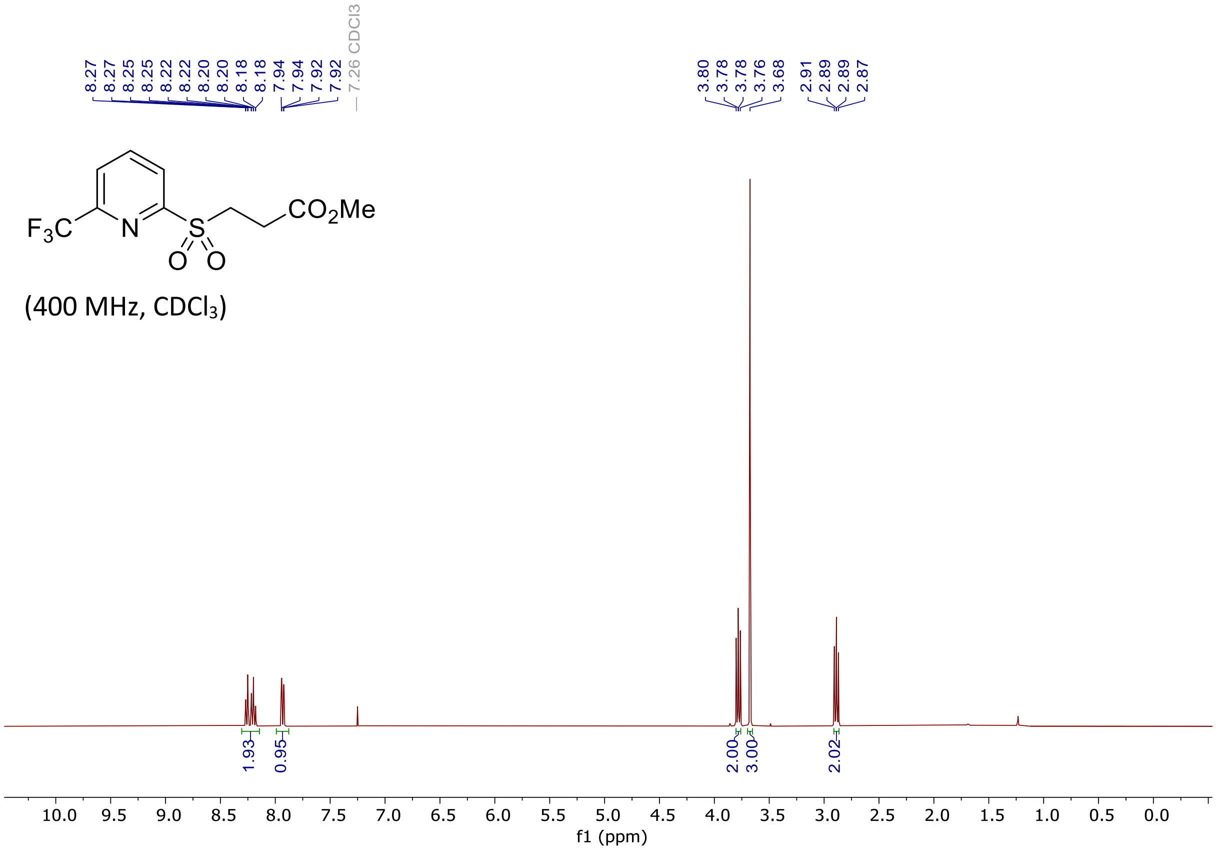

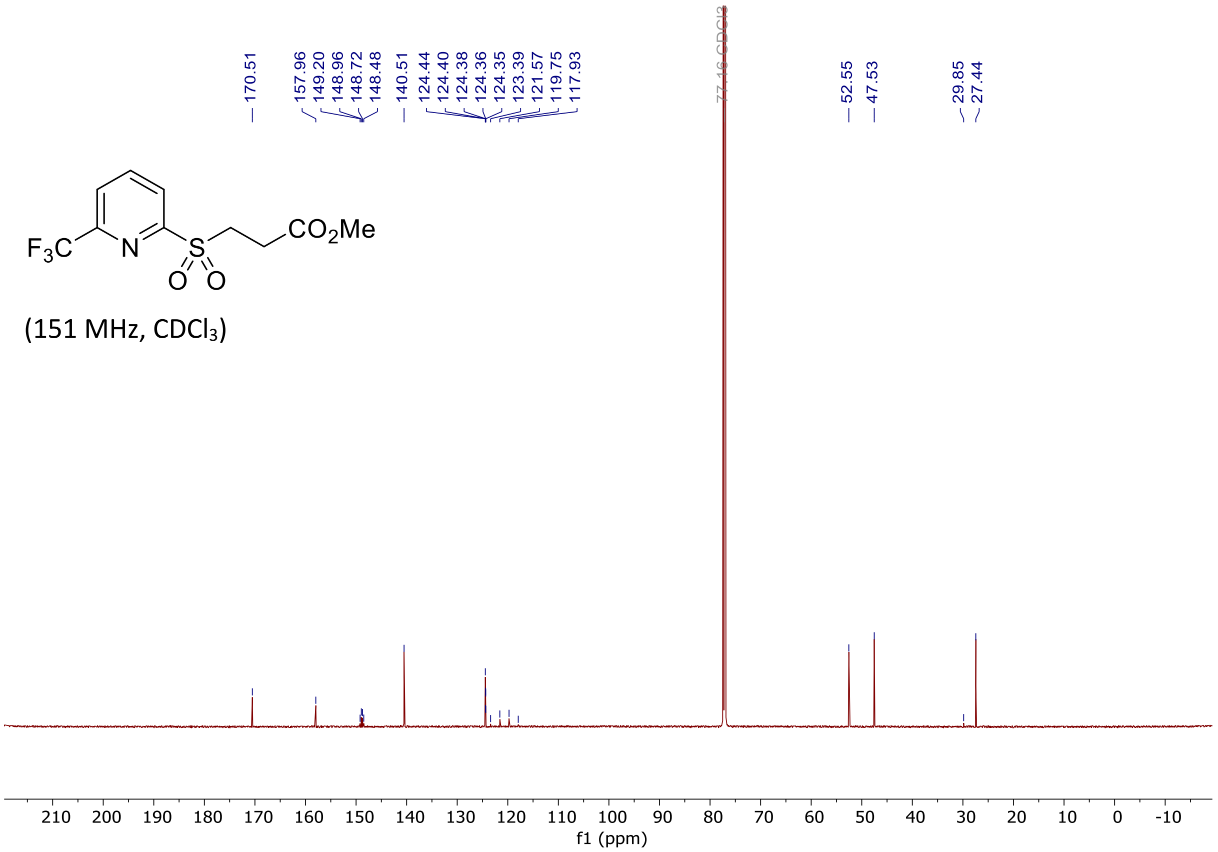

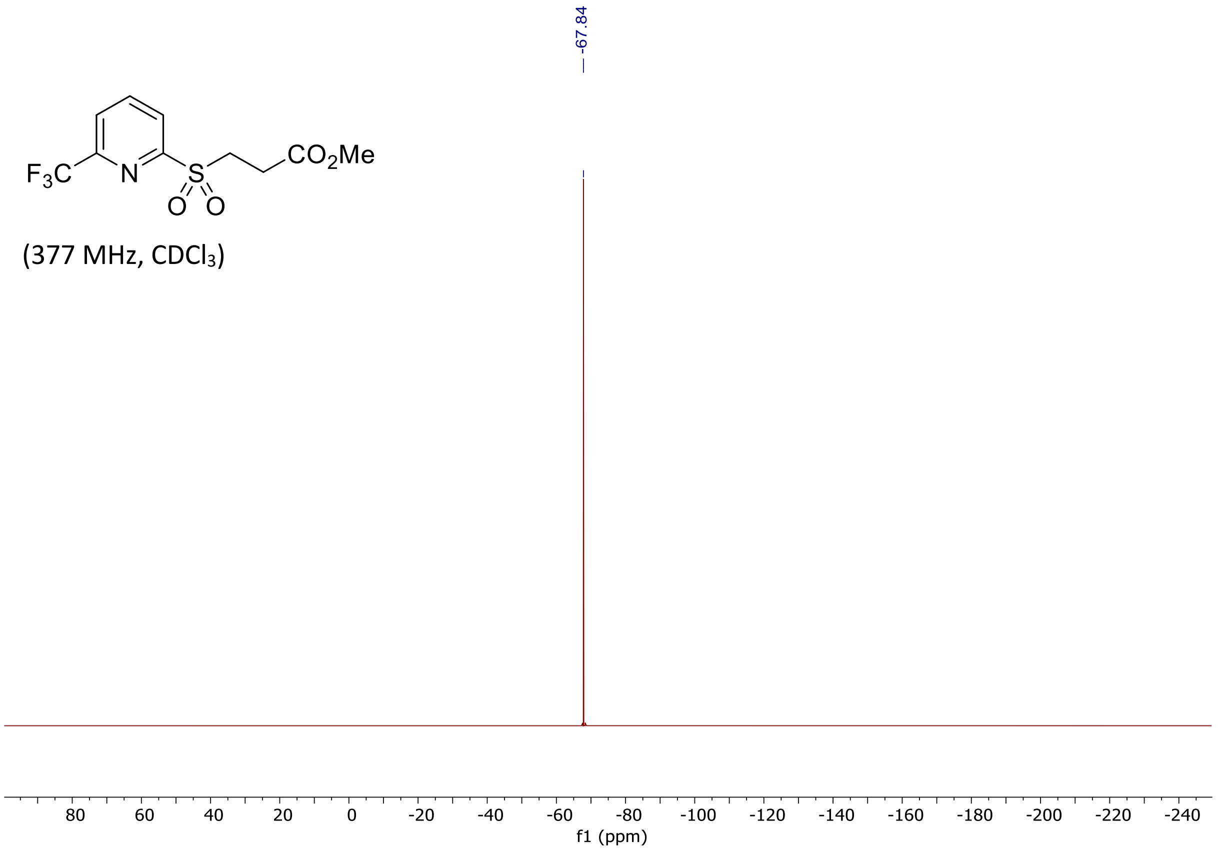
F3C
N
S
O O
CO2Me
(377 MHz, CDCl3)
-67.84
80
60
40
20
0
-20
-40
-60
-80
-100
-120
-140
-160
-180
-200
-220
-240
f1 (ppm)

Methyl 3-((3-methylpyridin-2-yl)sulfonyl)propanoate (5c)

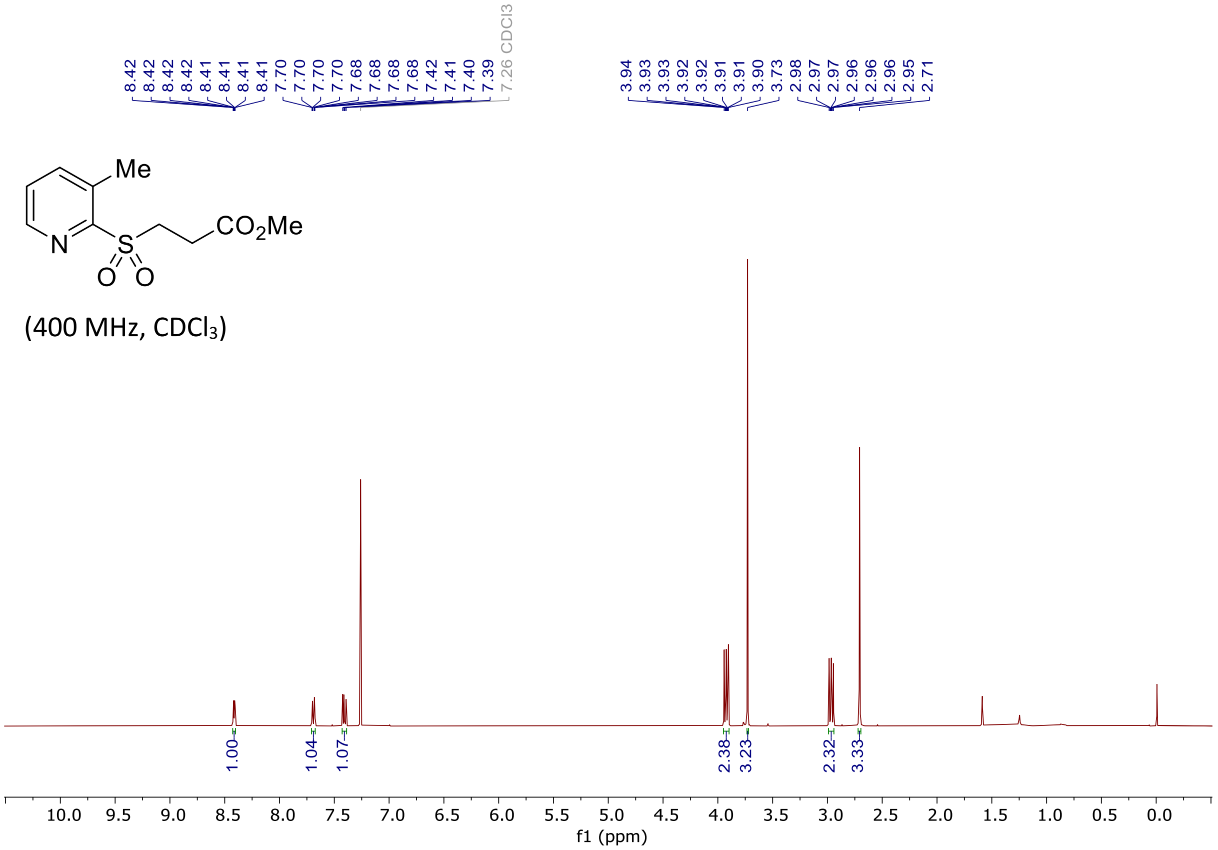

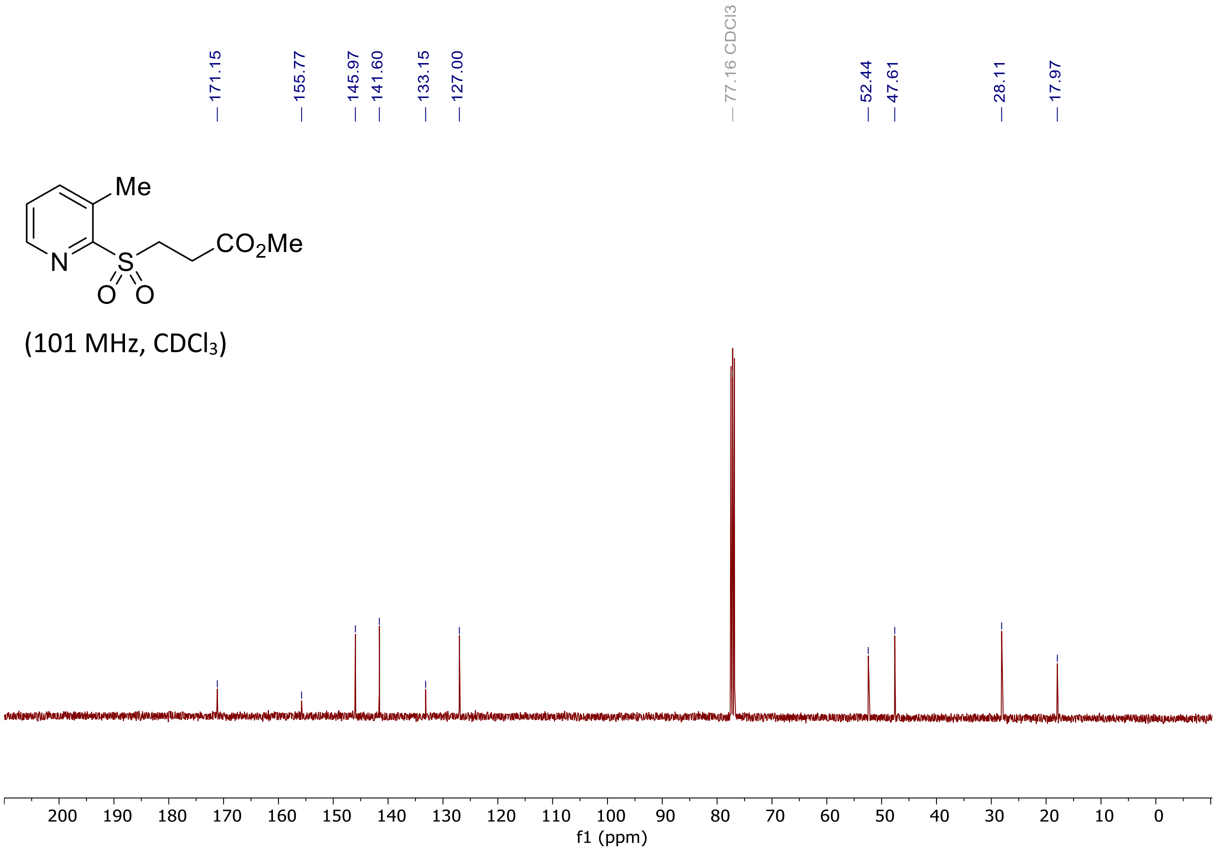

Methyl 3-((4-carbamoylpyridin-2-yl)sulfonyl)propanoate (5d)

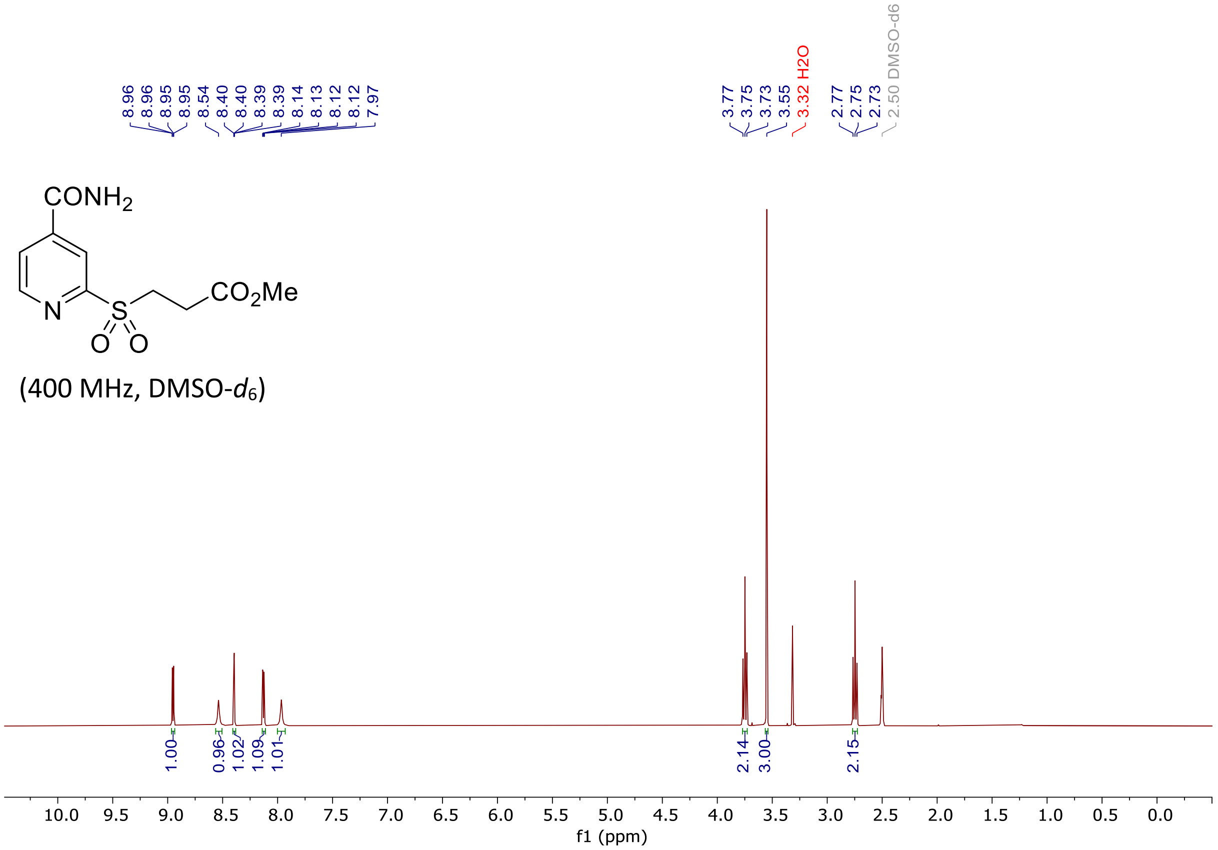

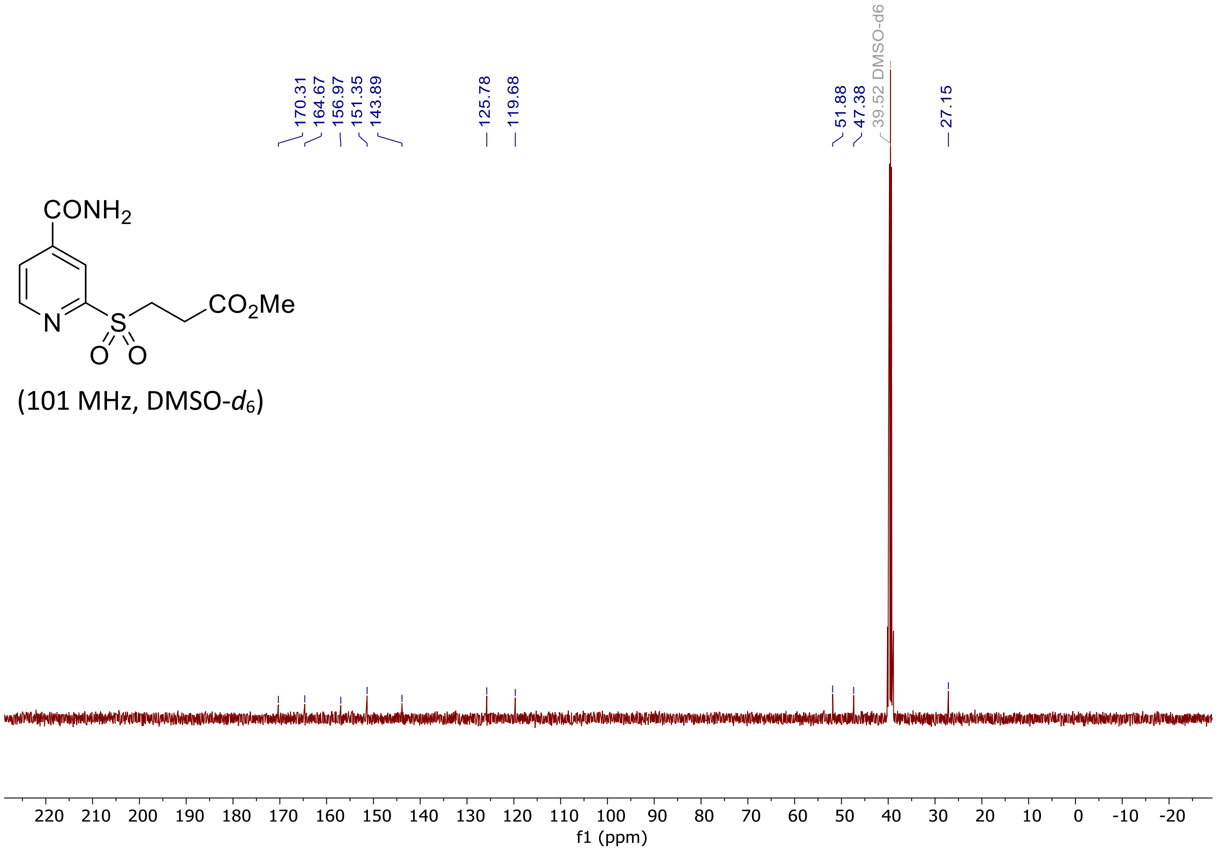

Methyl 3-((5-(trifluoromethyl)pyridin-2-yl)sulfonyl)propanoate (5e)

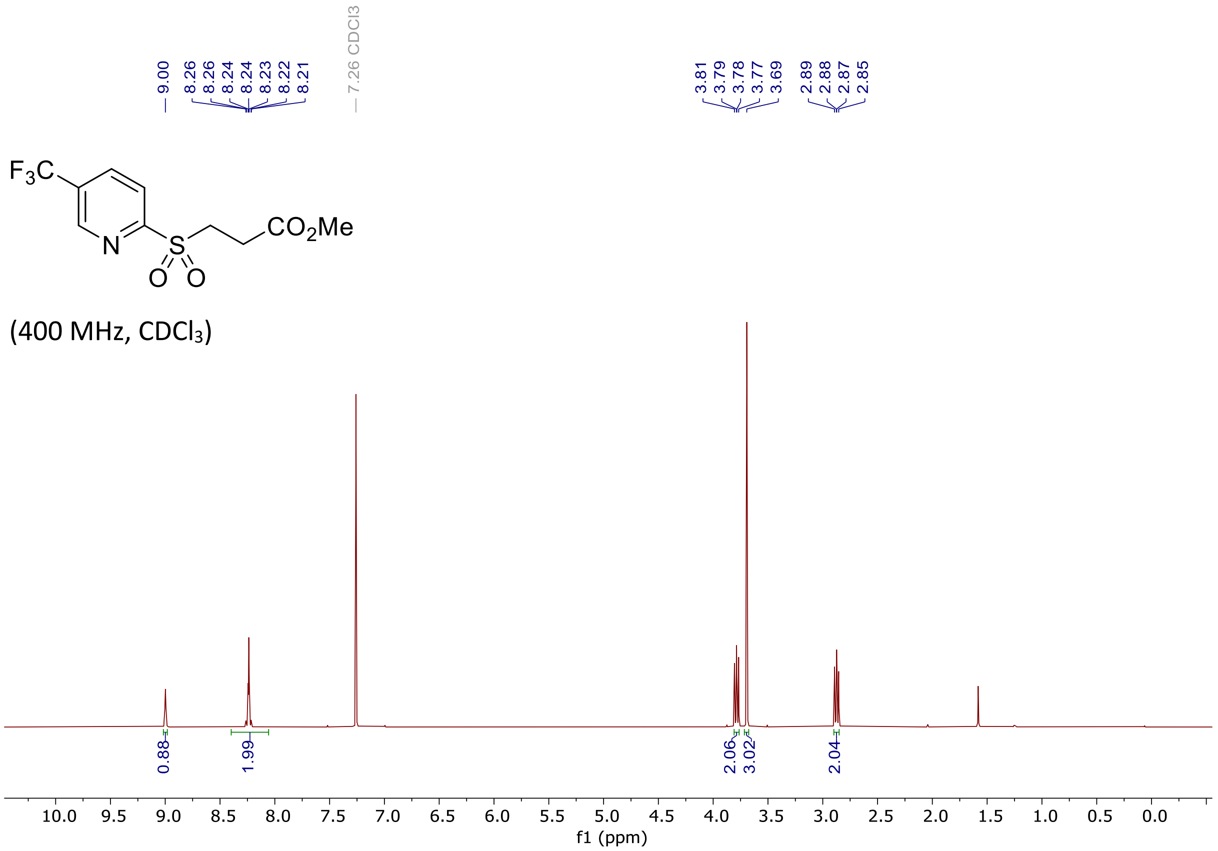

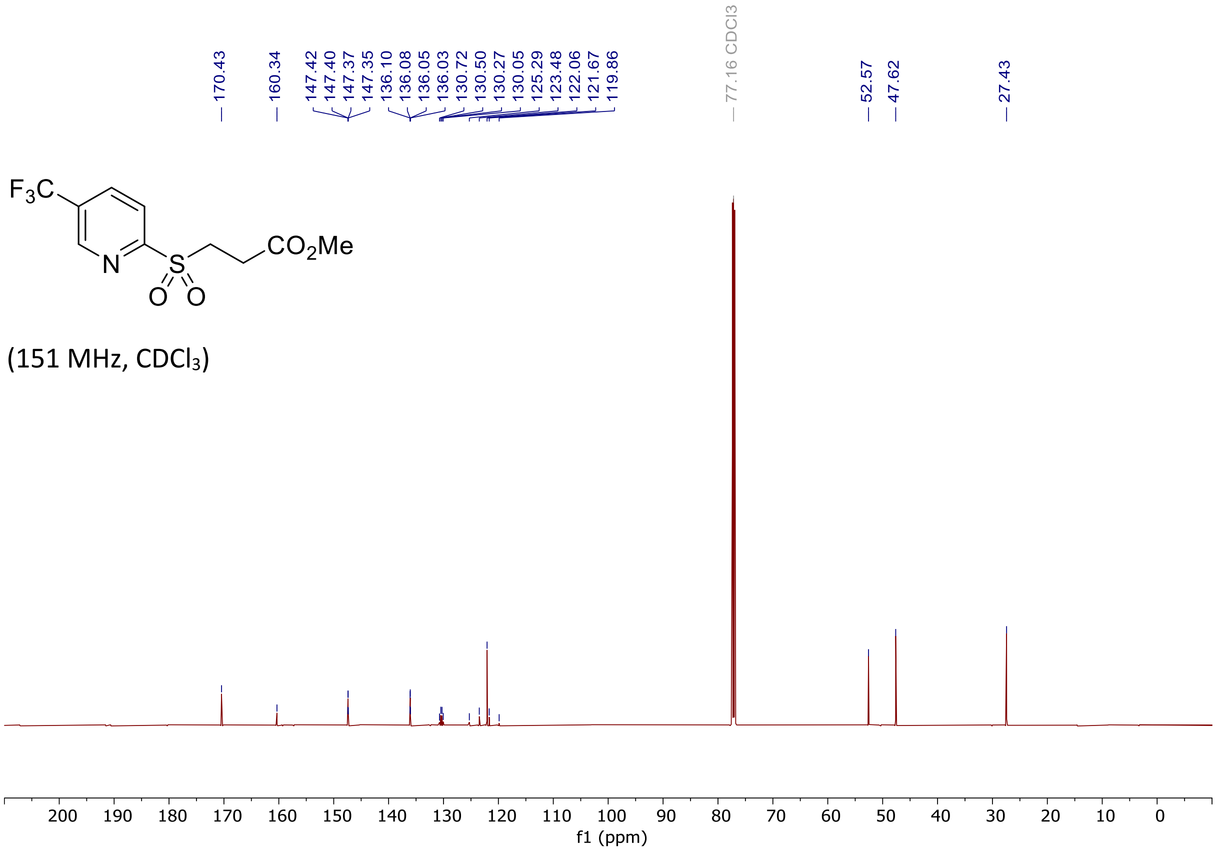

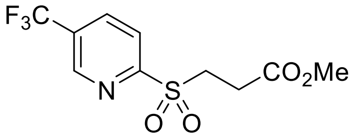

(377 MHz, $CDCl_3$)

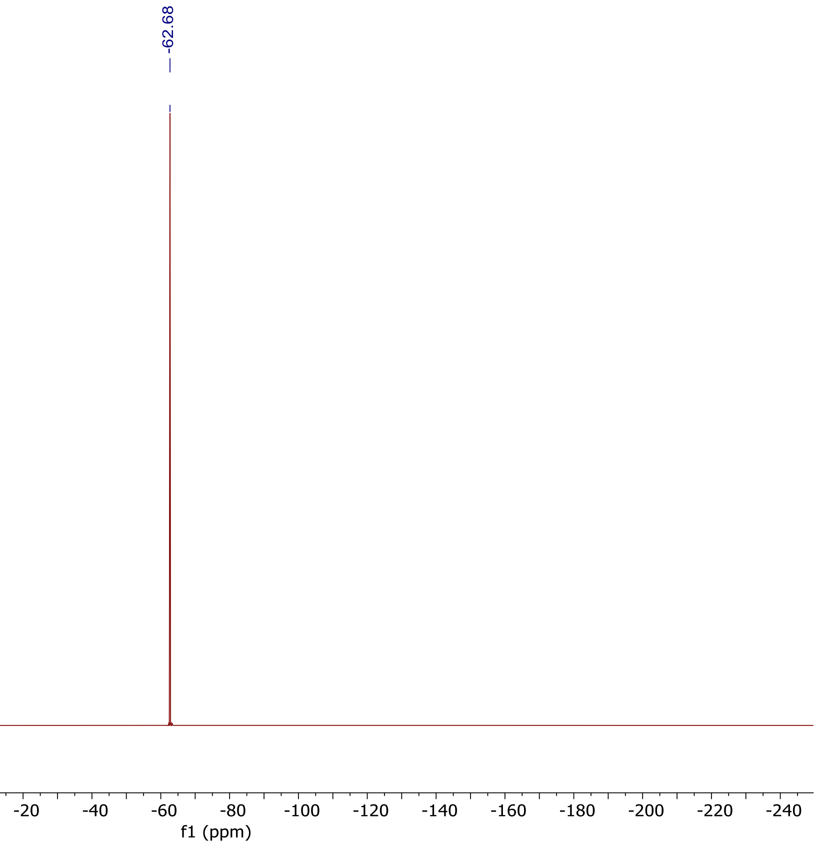

Methyl 3-((5-methoxypyridin-2-yl)sulfonyl)propanoate (5f)

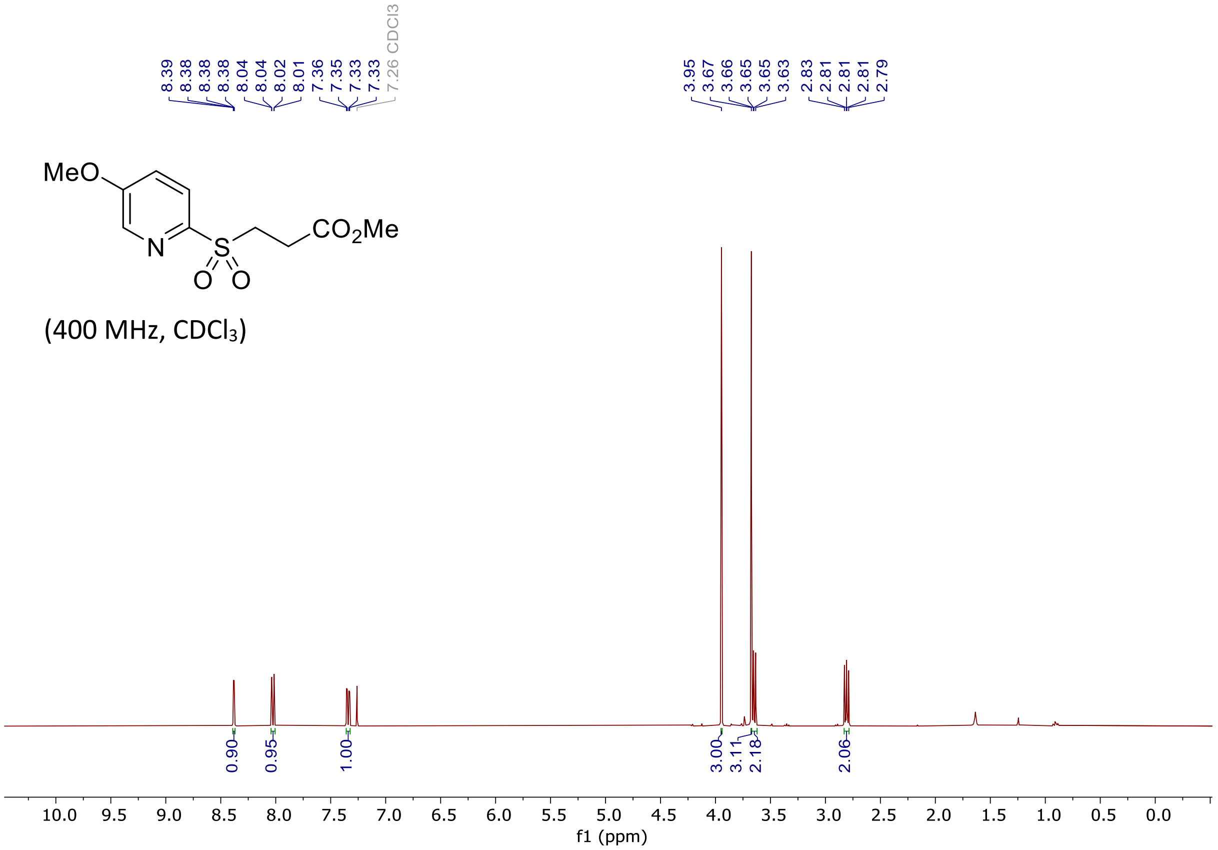

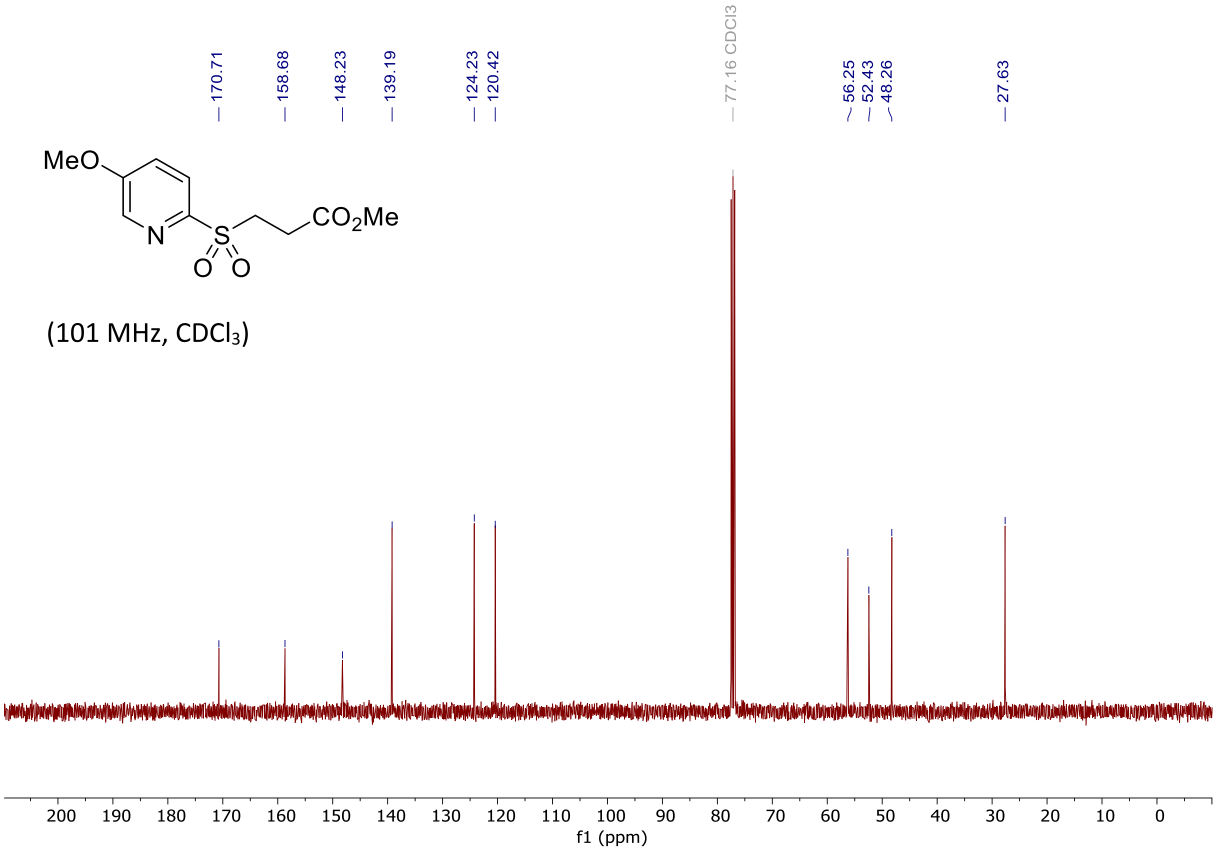

Methyl 3-(pyridin-3-ylsulfonyl)propanoate (5g)

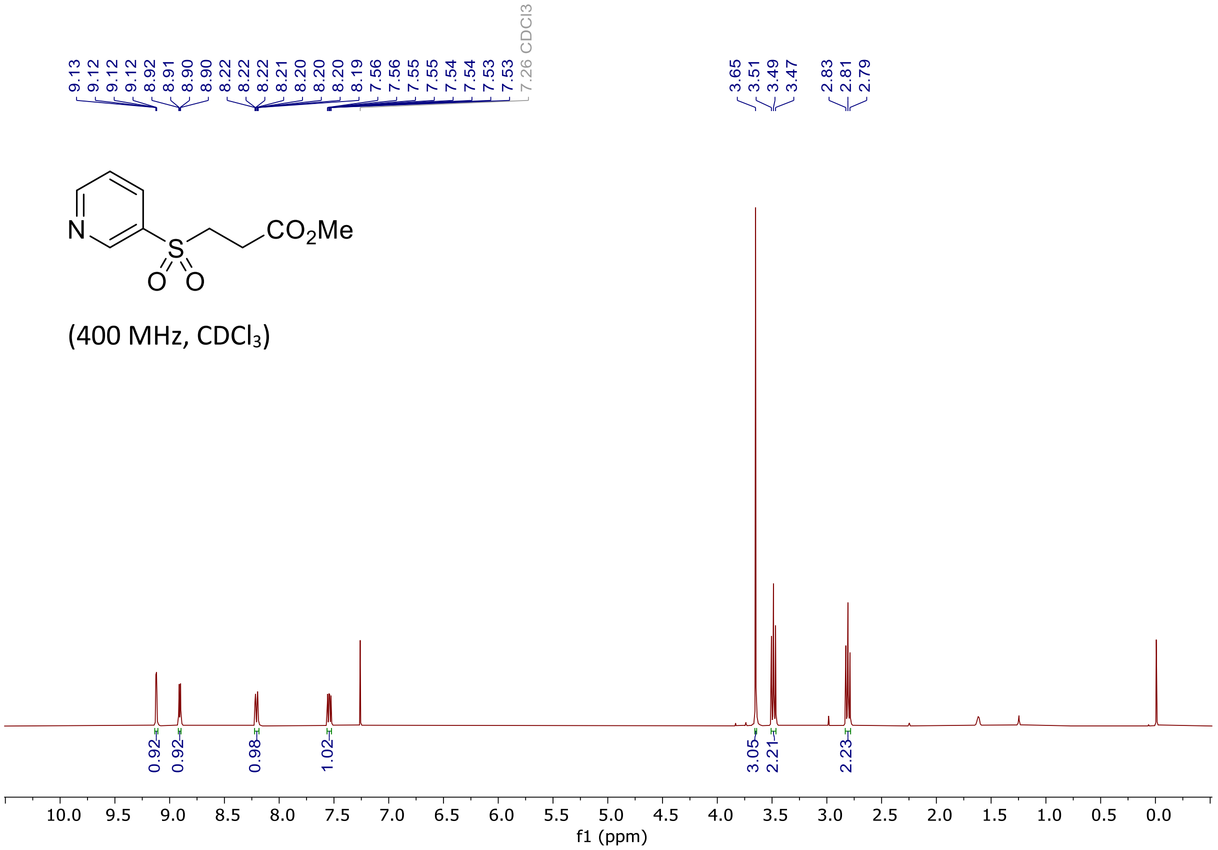

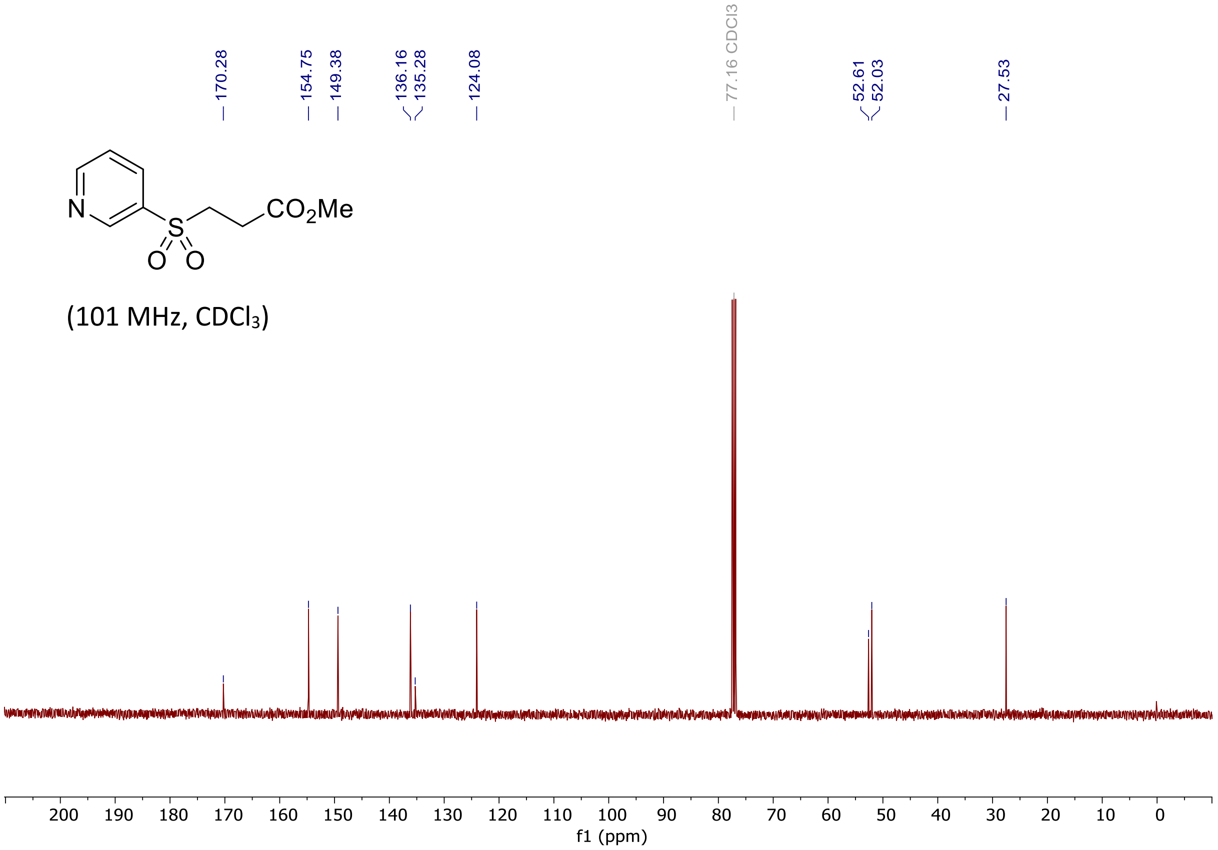

Methyl 3-((6-methoxypyridin-3-yl)sulfonyl)propanoate (5h)

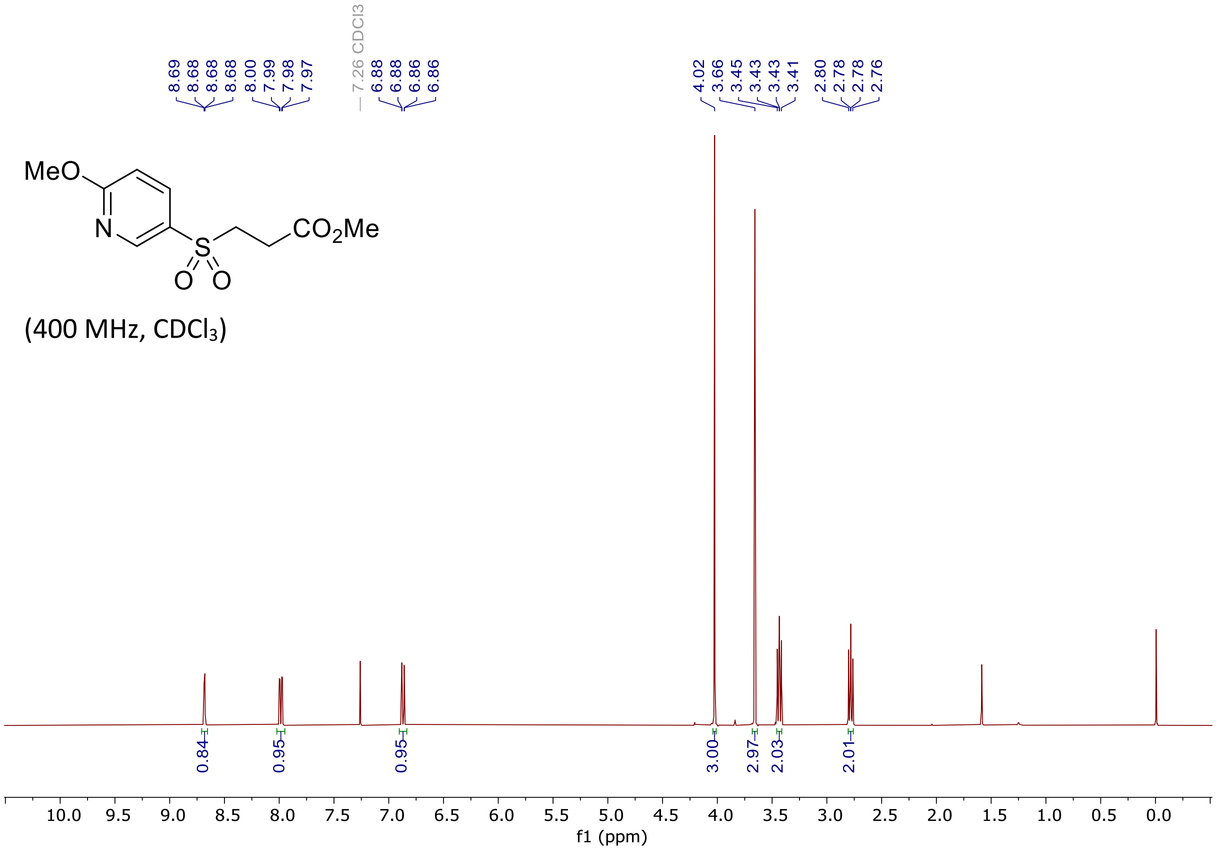

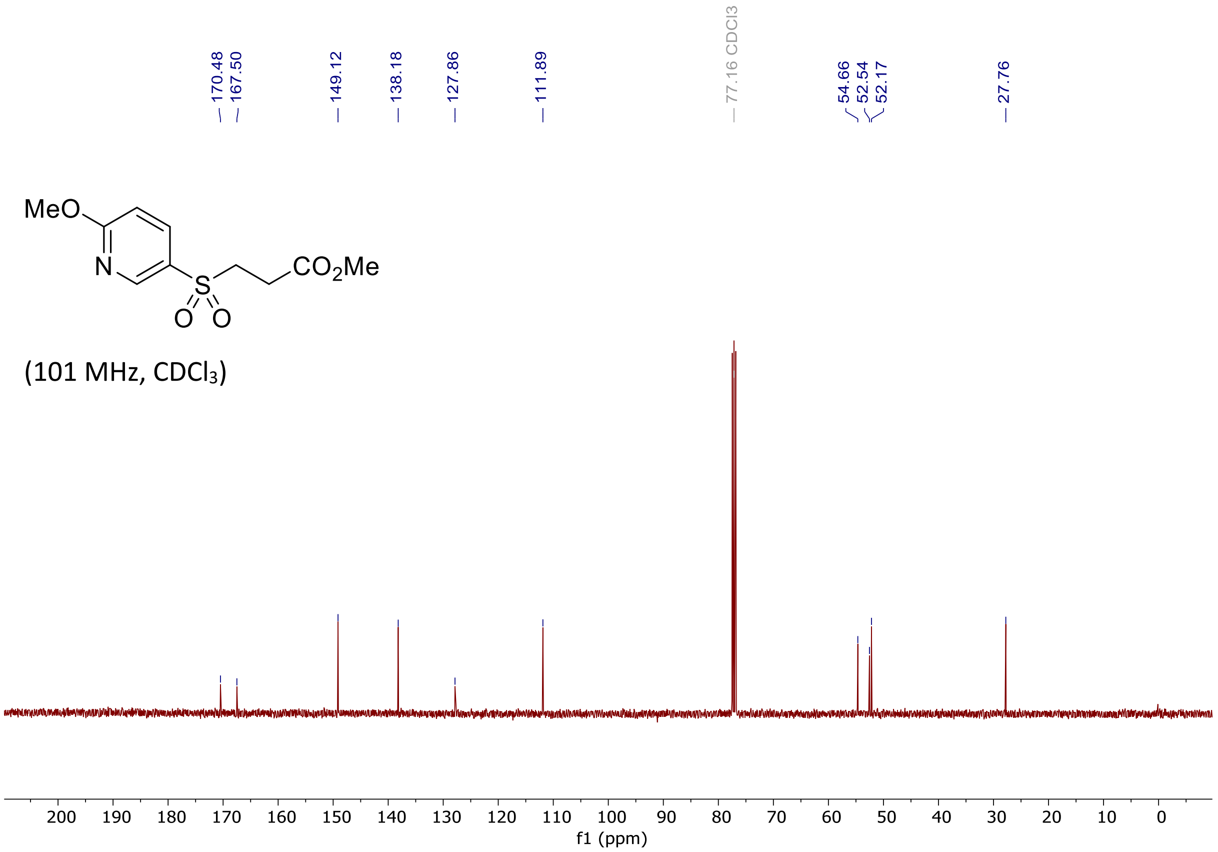

Methyl 3-(pyridin-4-ylsulfonyl)propanoate (5i)

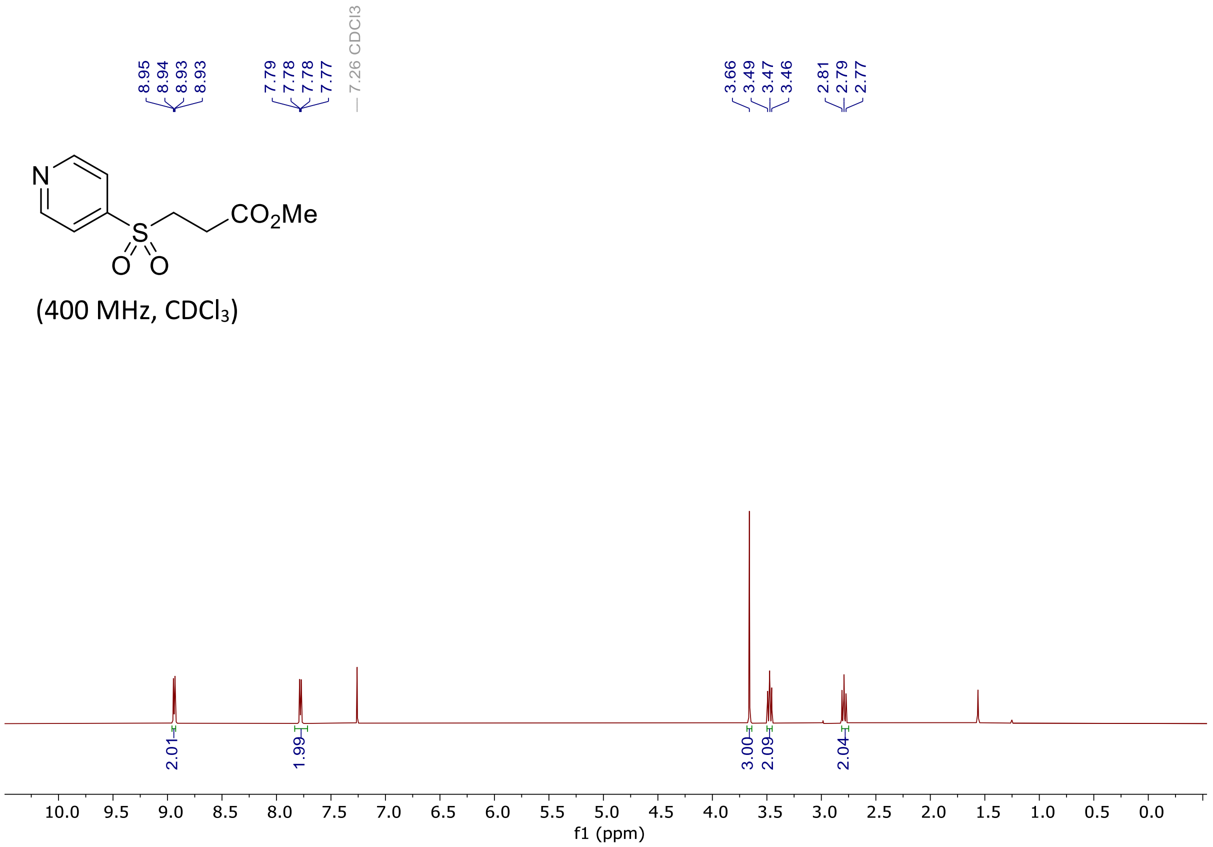

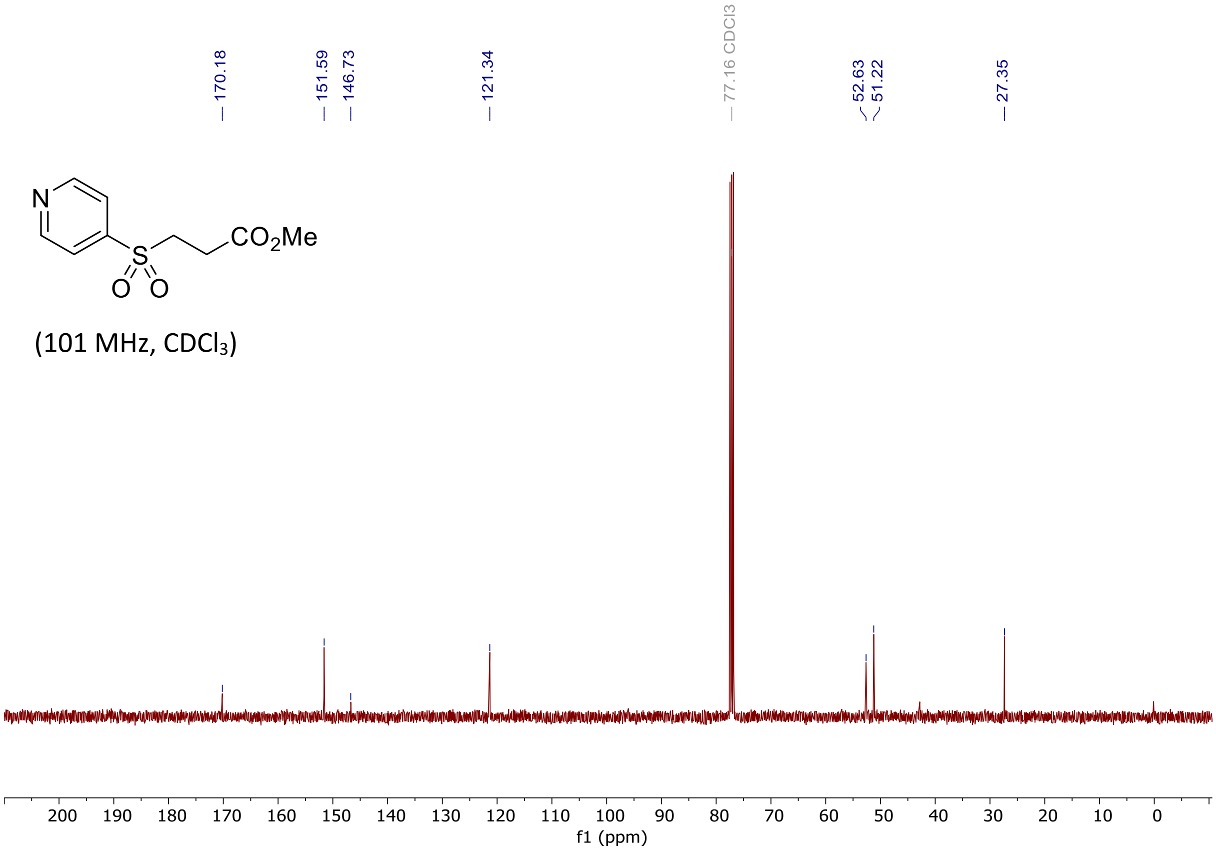

Methyl 3-(pyrazin-2-ylsulfonyl)propanoate (5j)

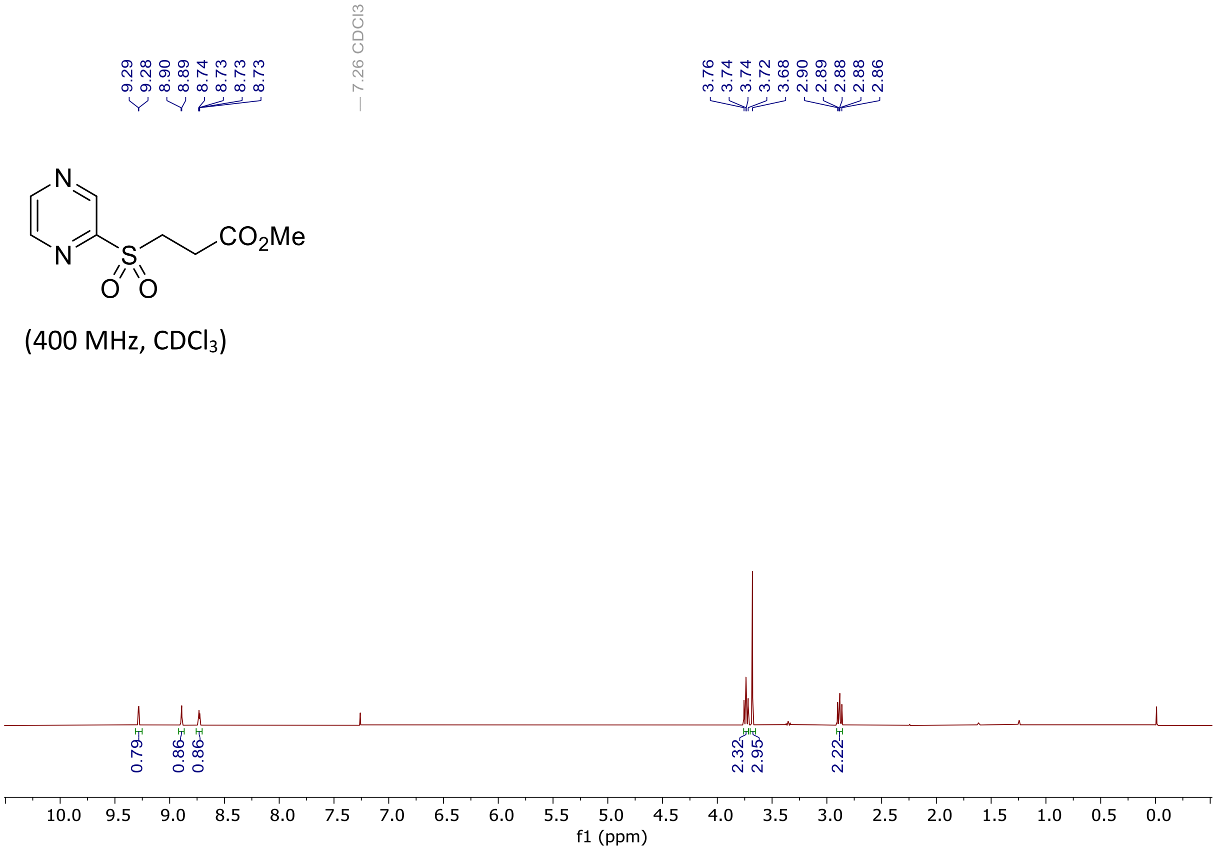

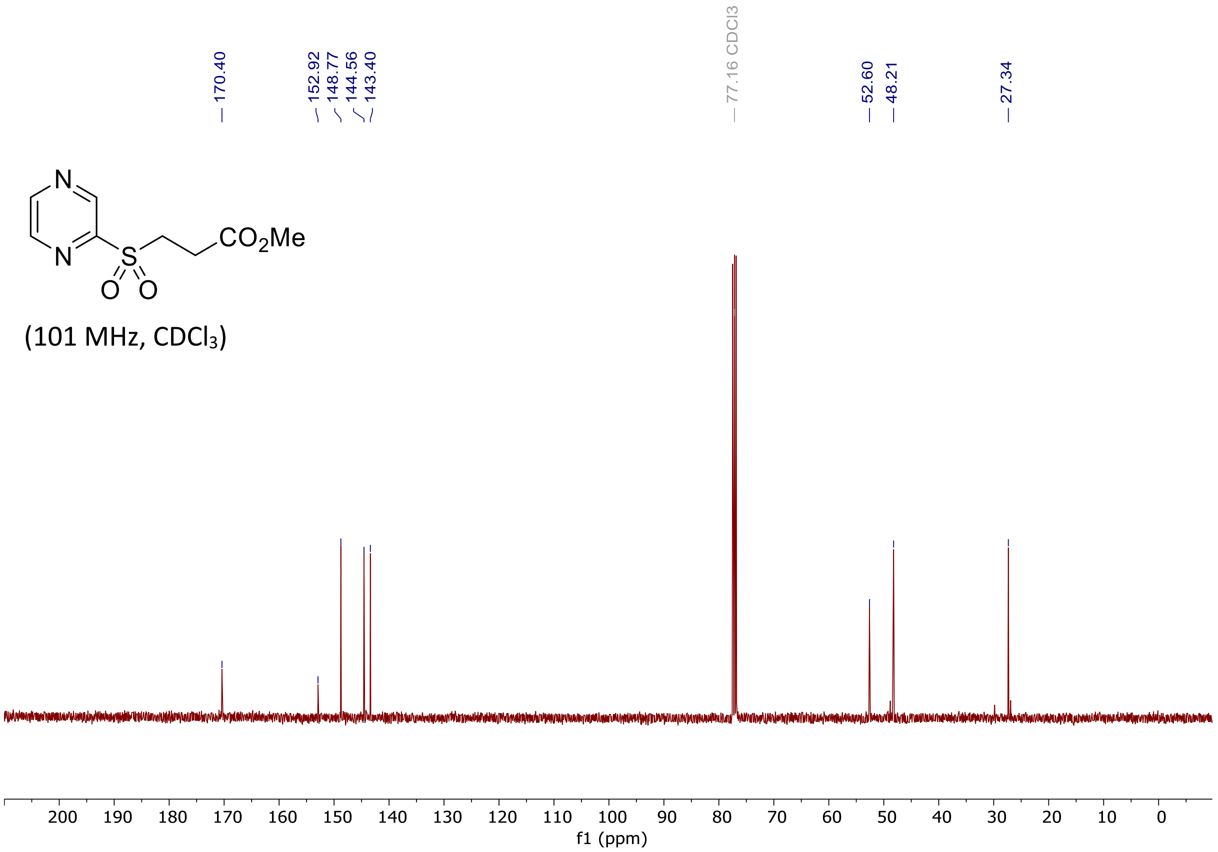

Methyl 3-(pyrimidin-2-ylsulfonyl)propanoate (5k)

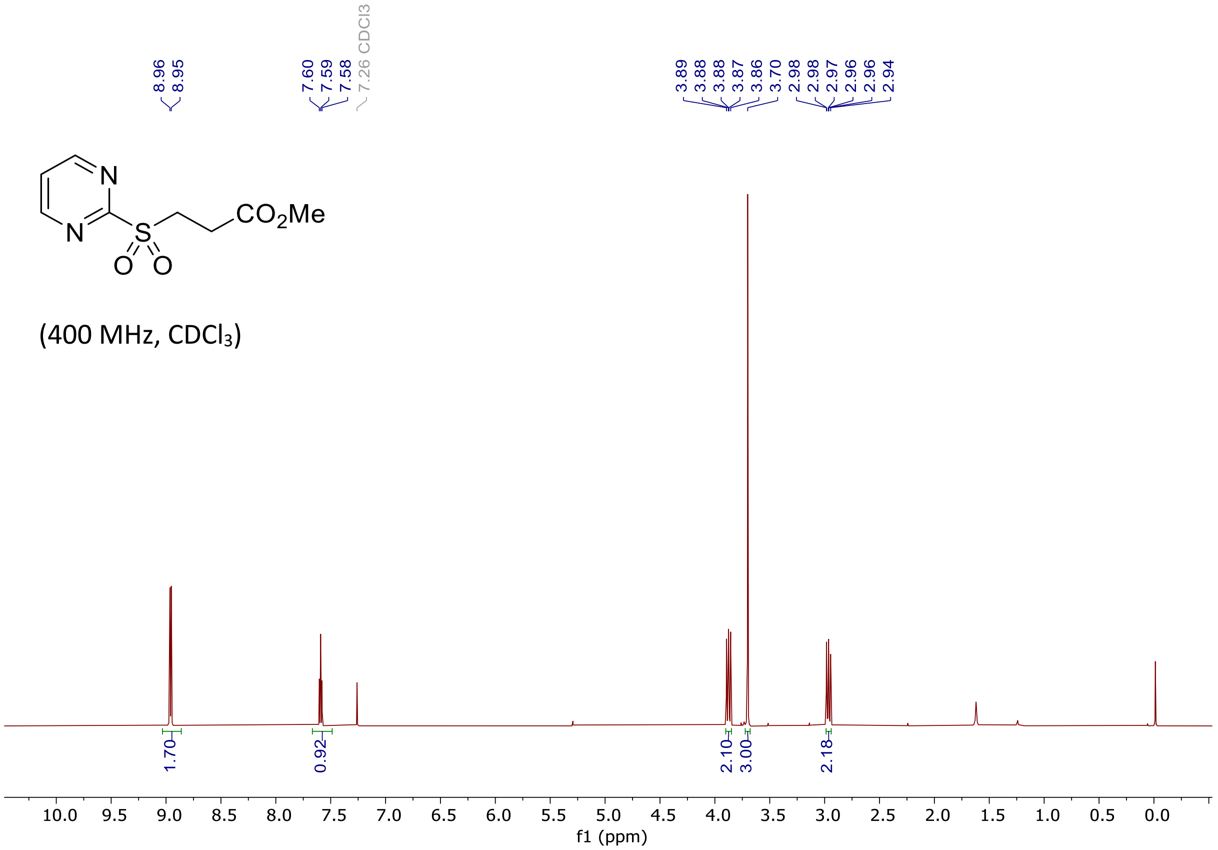

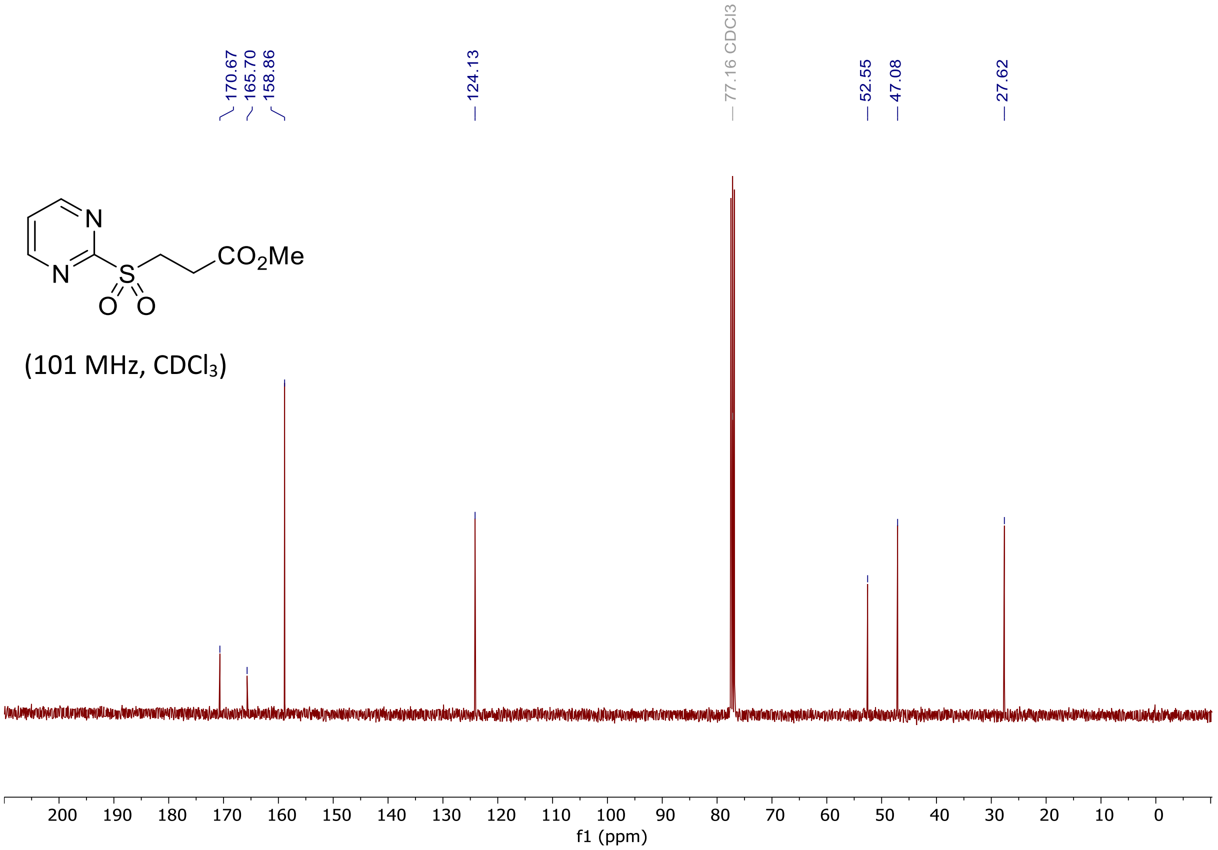

Methyl 3-(isoquinolin-1-ylsulfonyl)propanoate (5l)

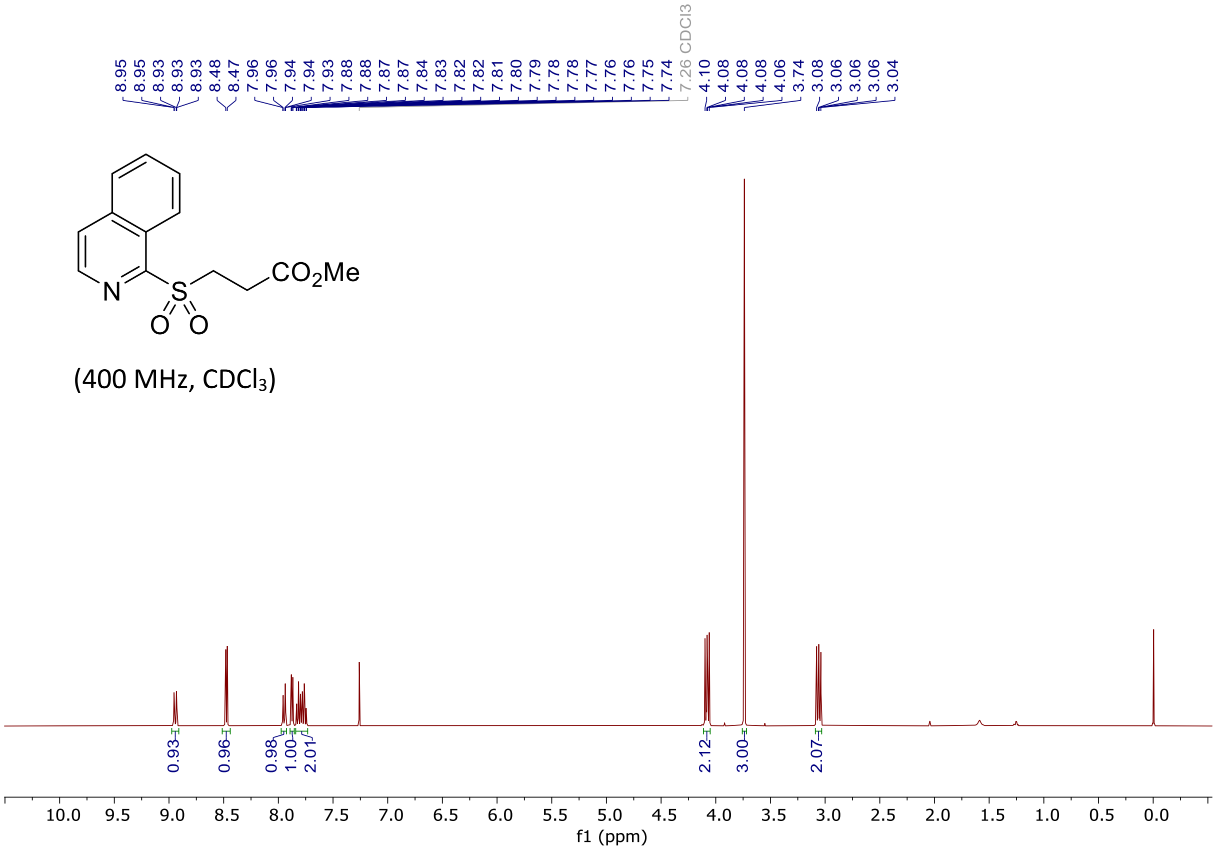

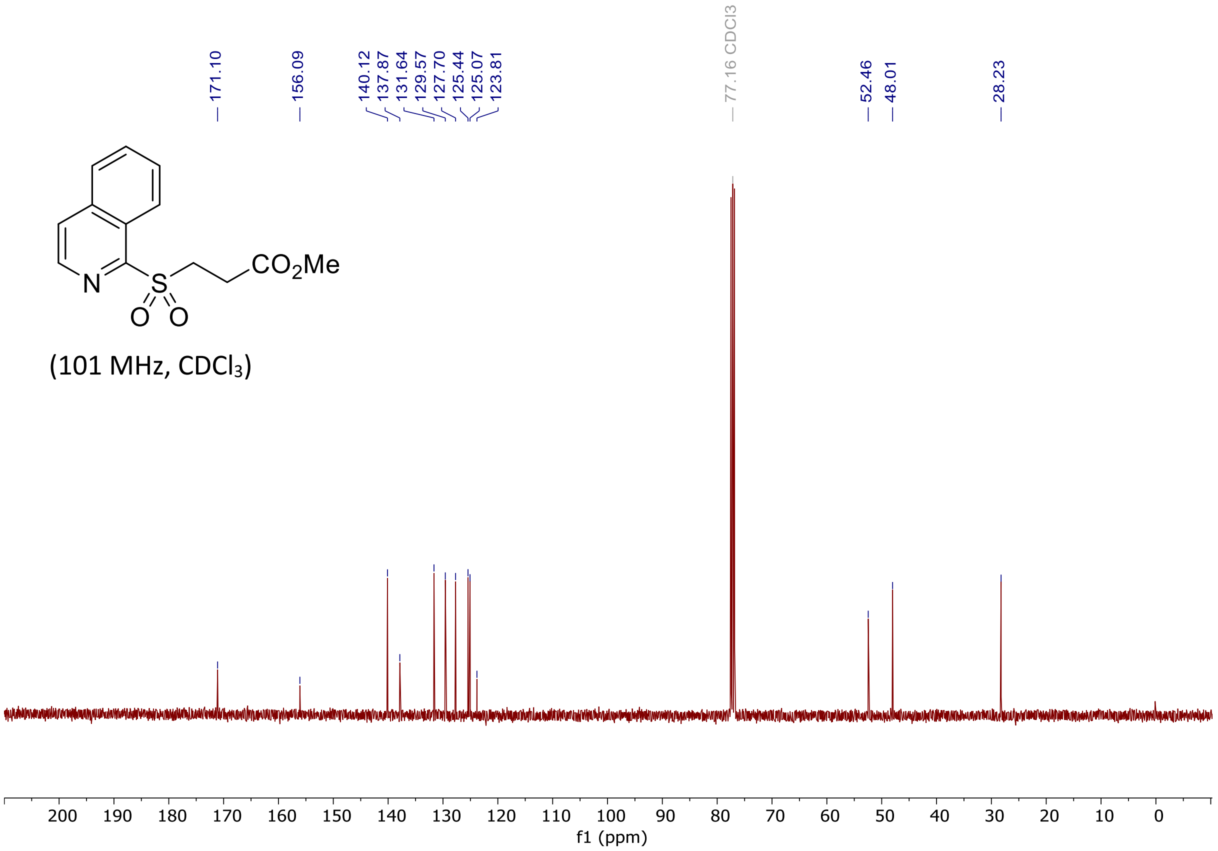

Methyl 3-(phenylsulfonyl)propanoate (5m)

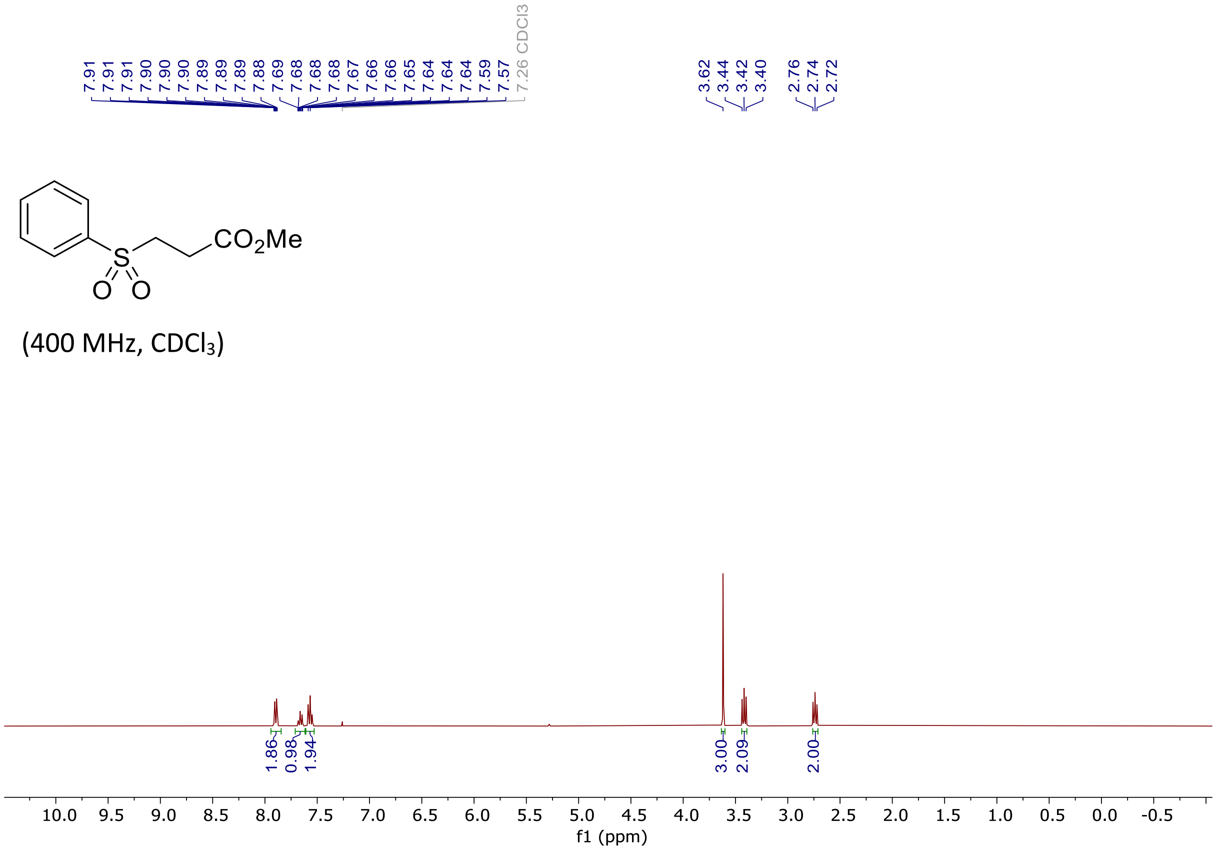

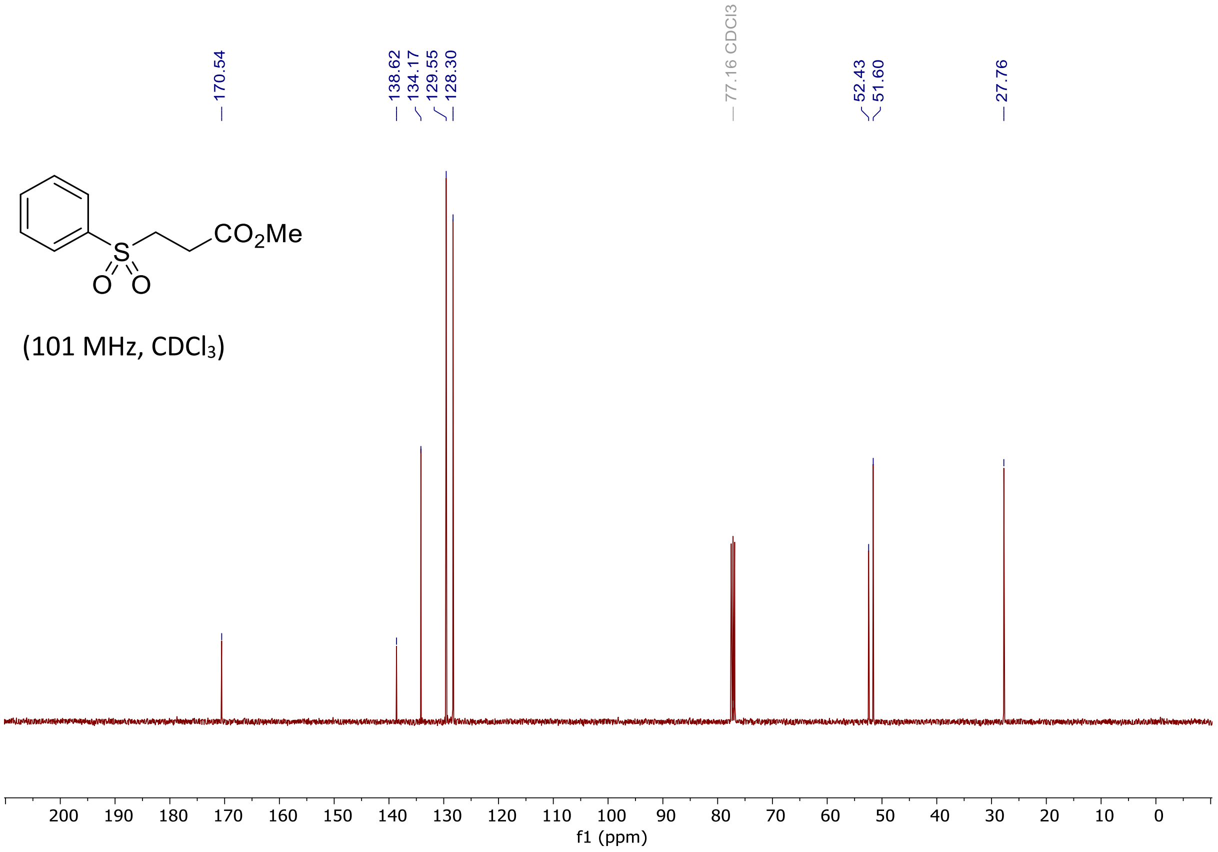

Methyl 3-((4-methoxyphenyl)sulfonyl)propanoate (5n)

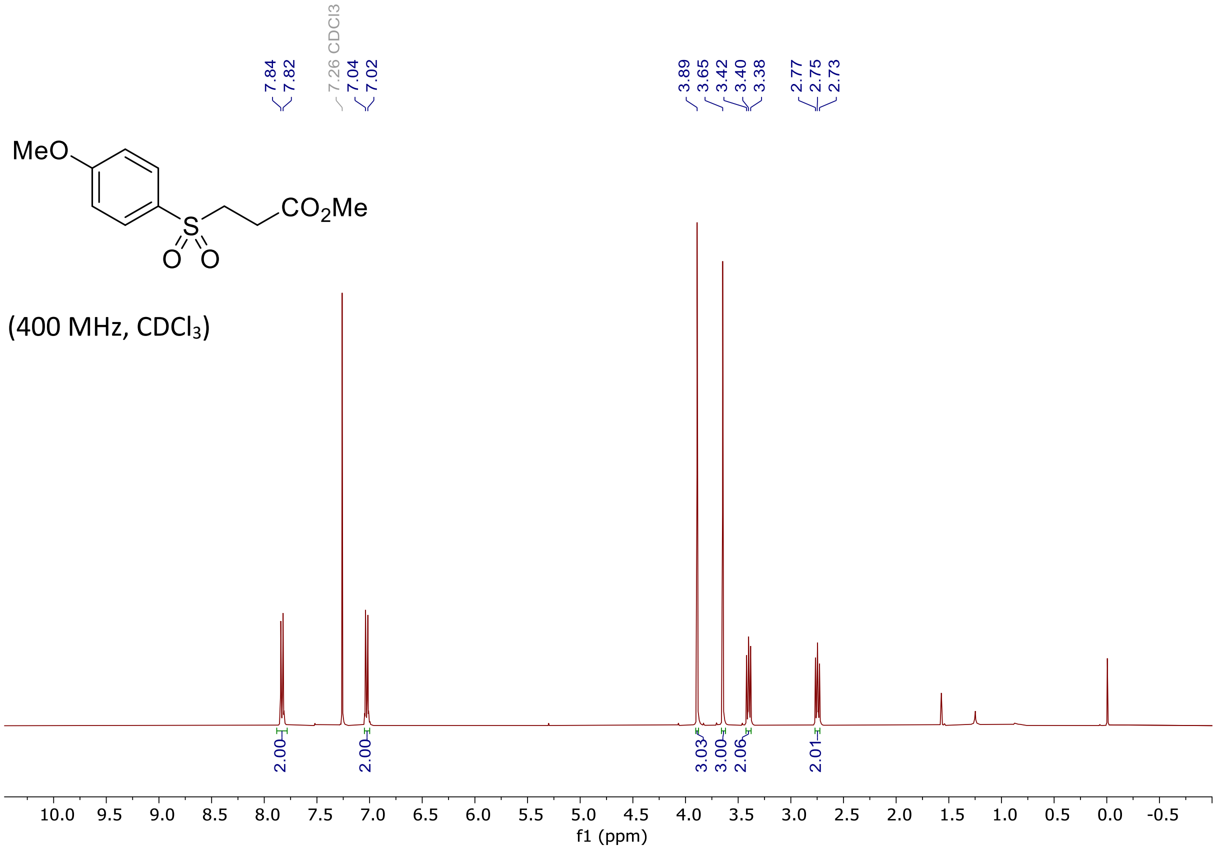

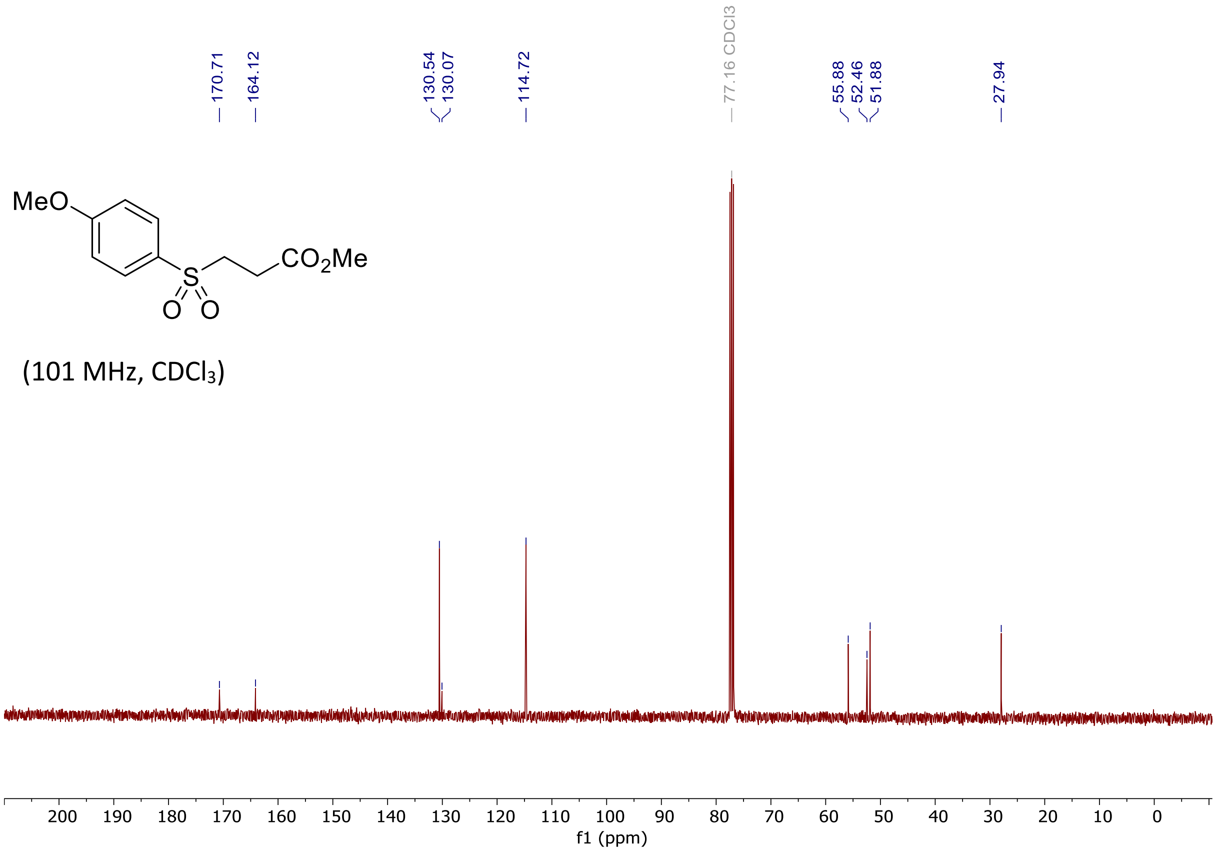

Methyl 4-((3-methoxy-3-oxopropyl)sulfonyl)benzoate (5o)

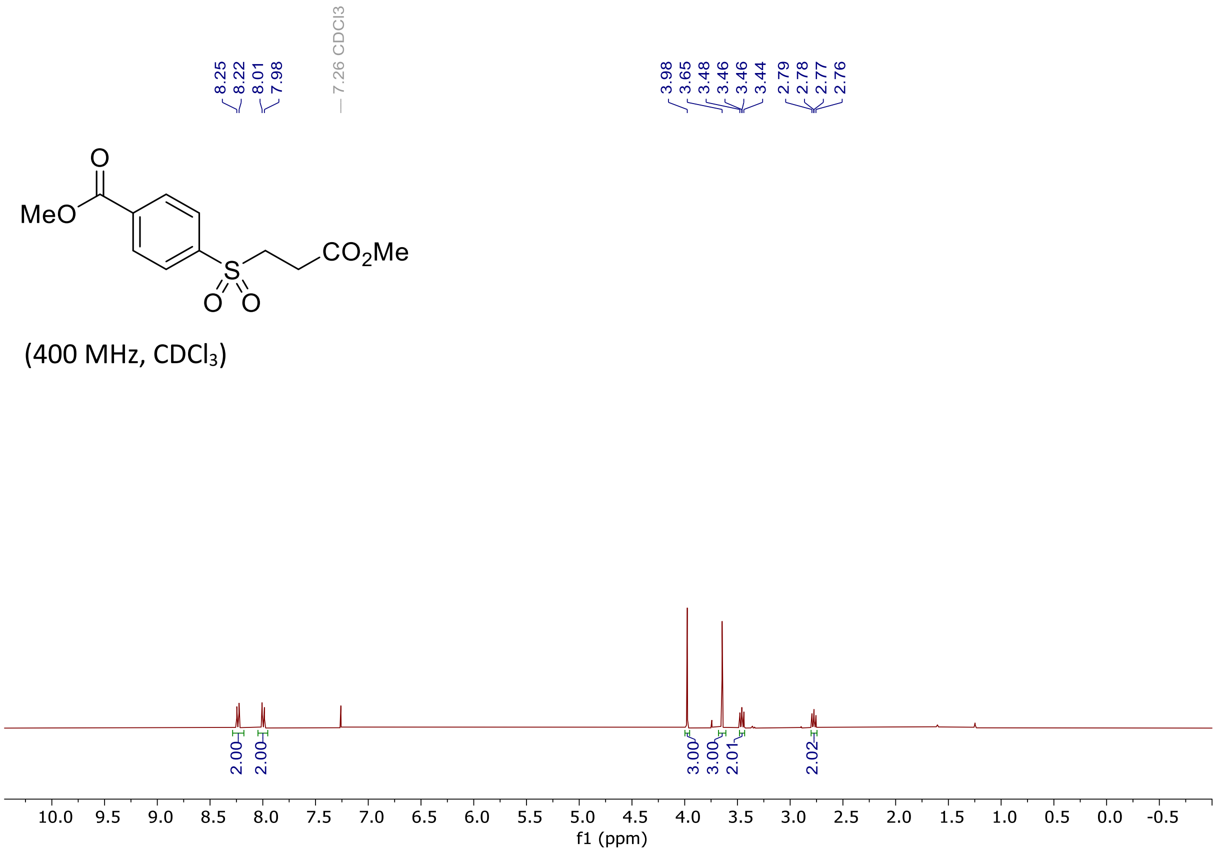

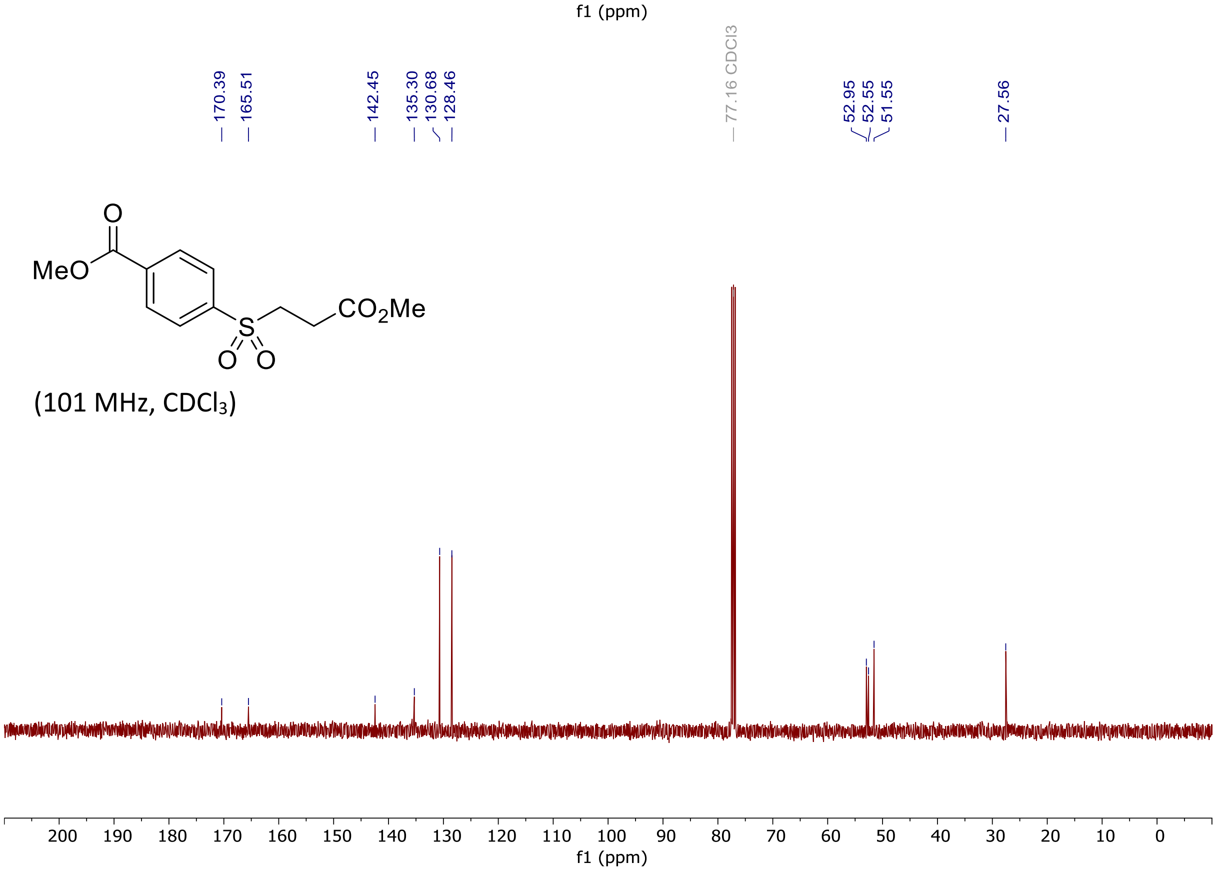

Methyl 3-((4-(trifluoromethyl)phenyl)sulfonyl)propanoate (5p)

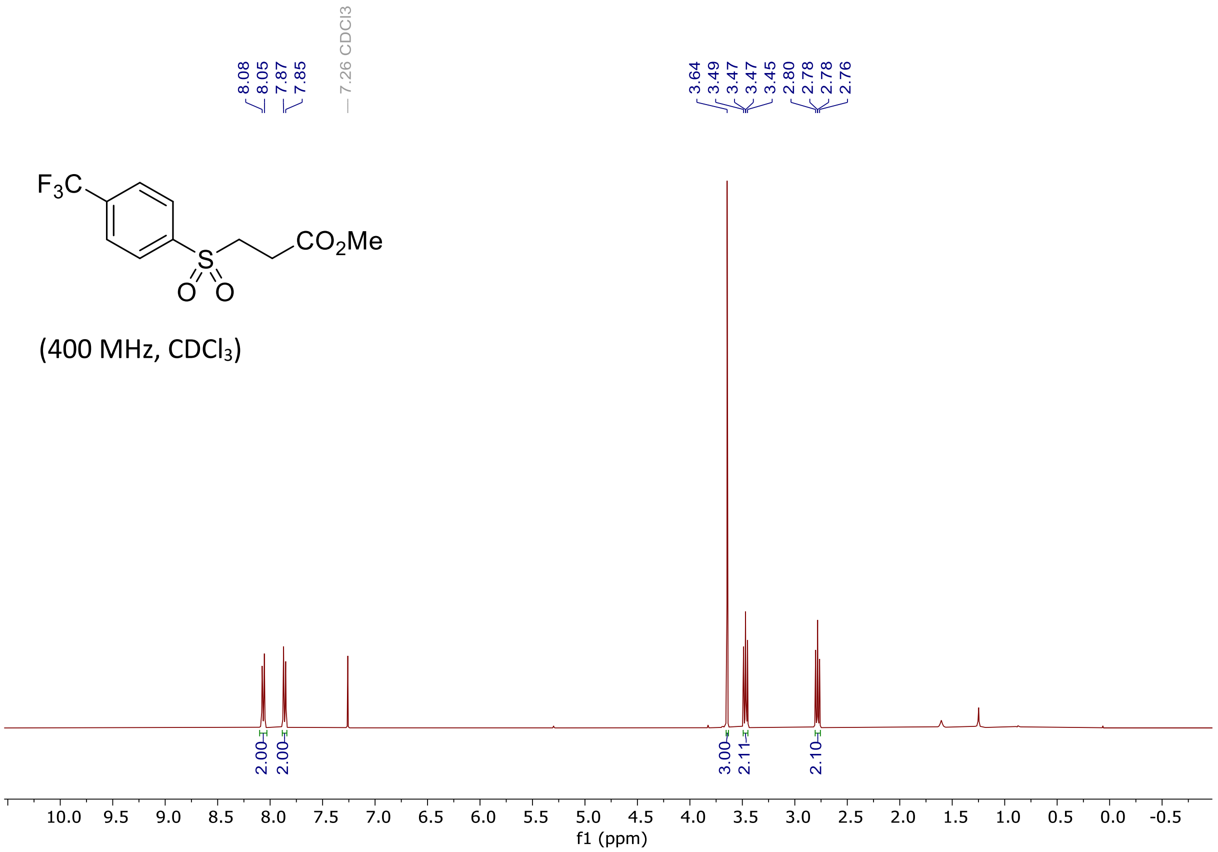

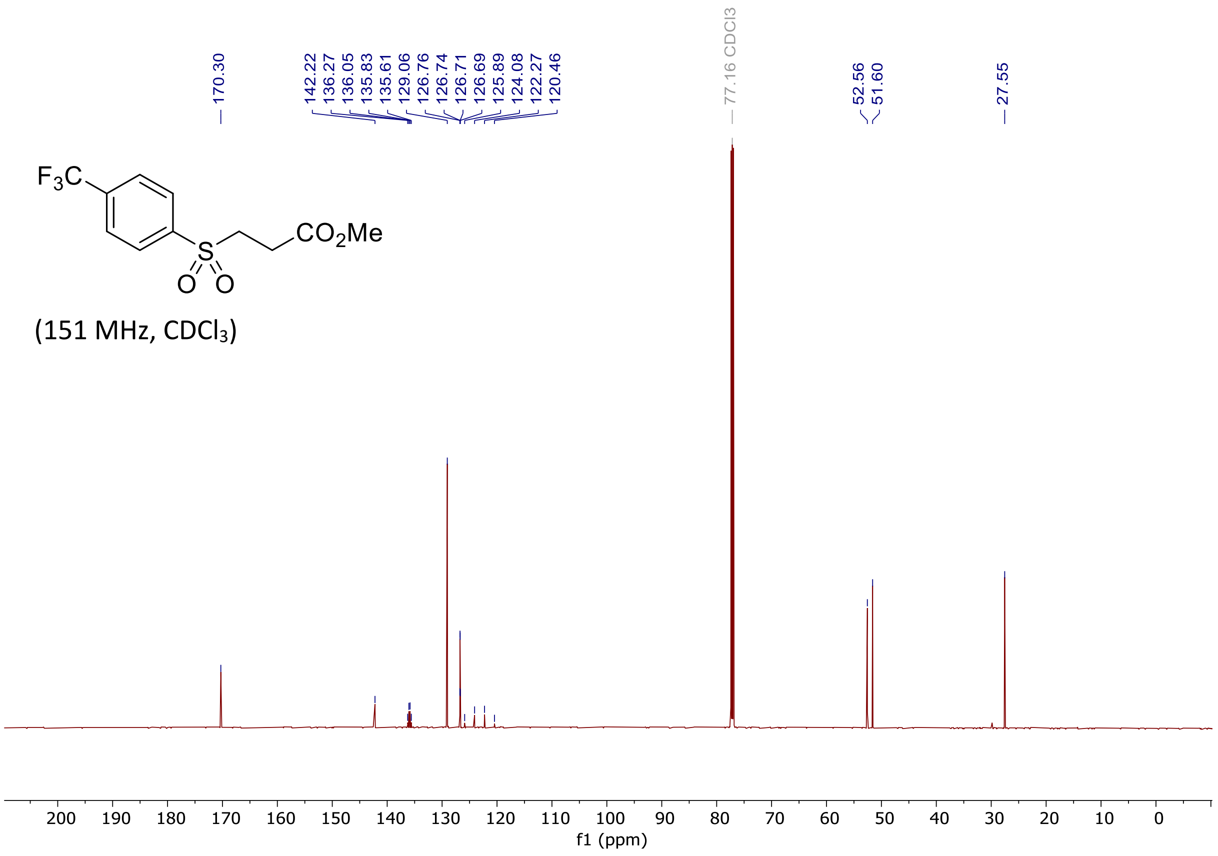

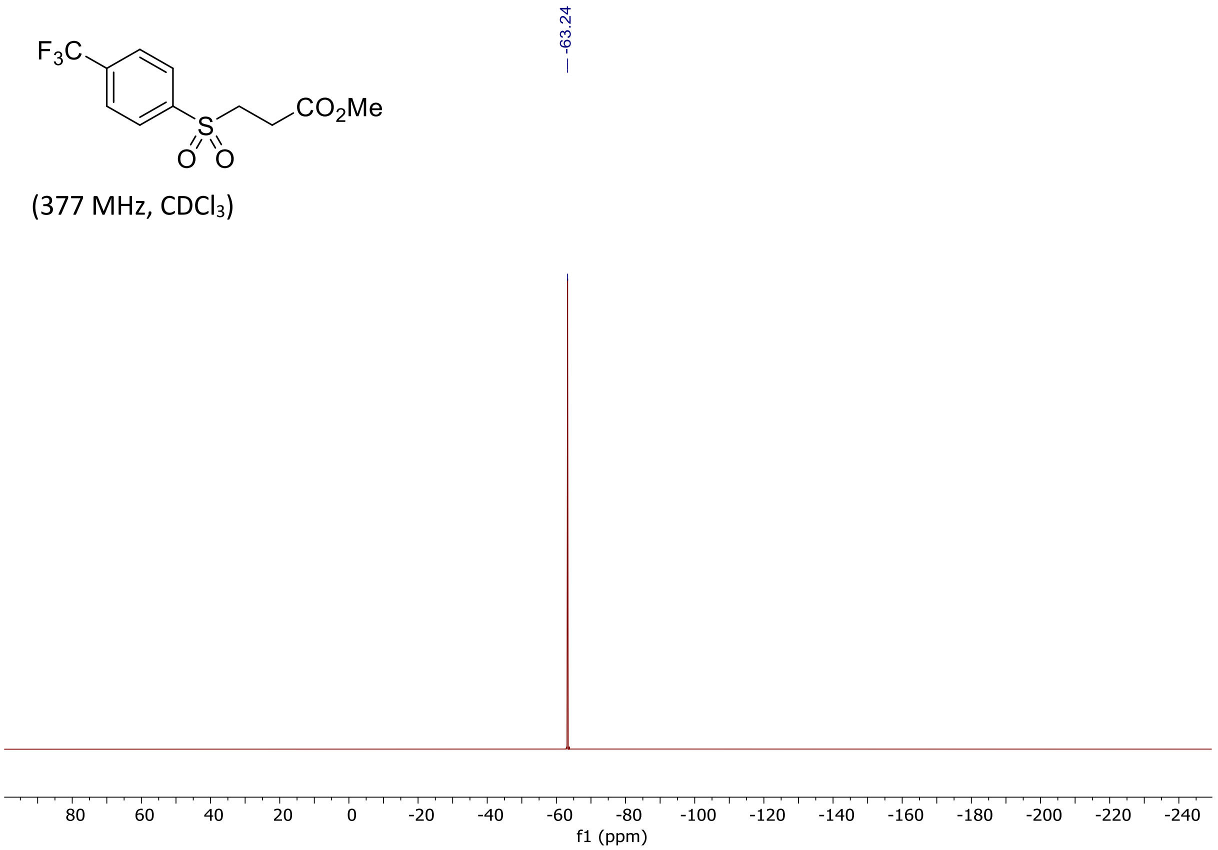
F_3C
CO_2Me
S
O O
(377 MHz, $CDCl_3$)
-63.24
80 60 40 20 0 -20 -40 -60 -80 -100 -120 -140 -160 -180 -200 -220 -240
f1 (ppm)

tert-Butyl 3-(pyridin-2-ylsulfonyl)propanoate (7a)

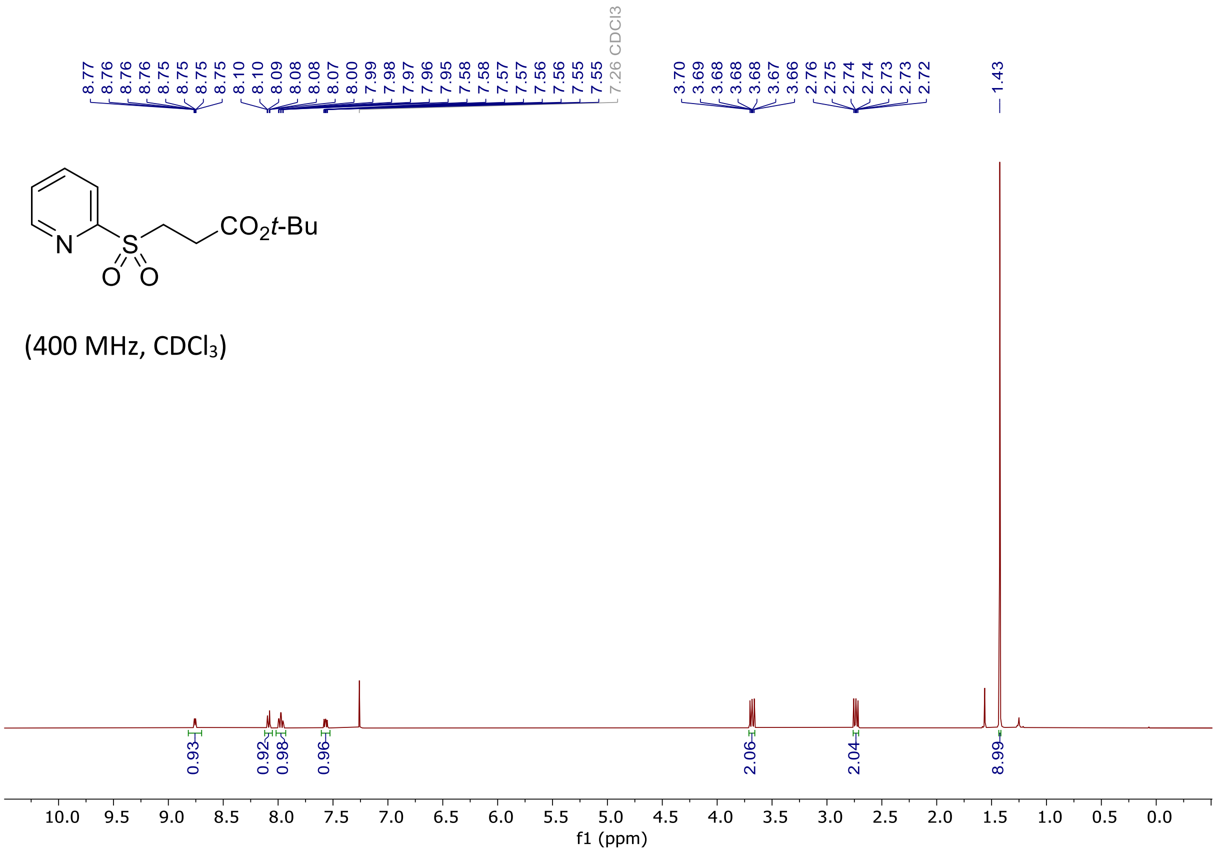

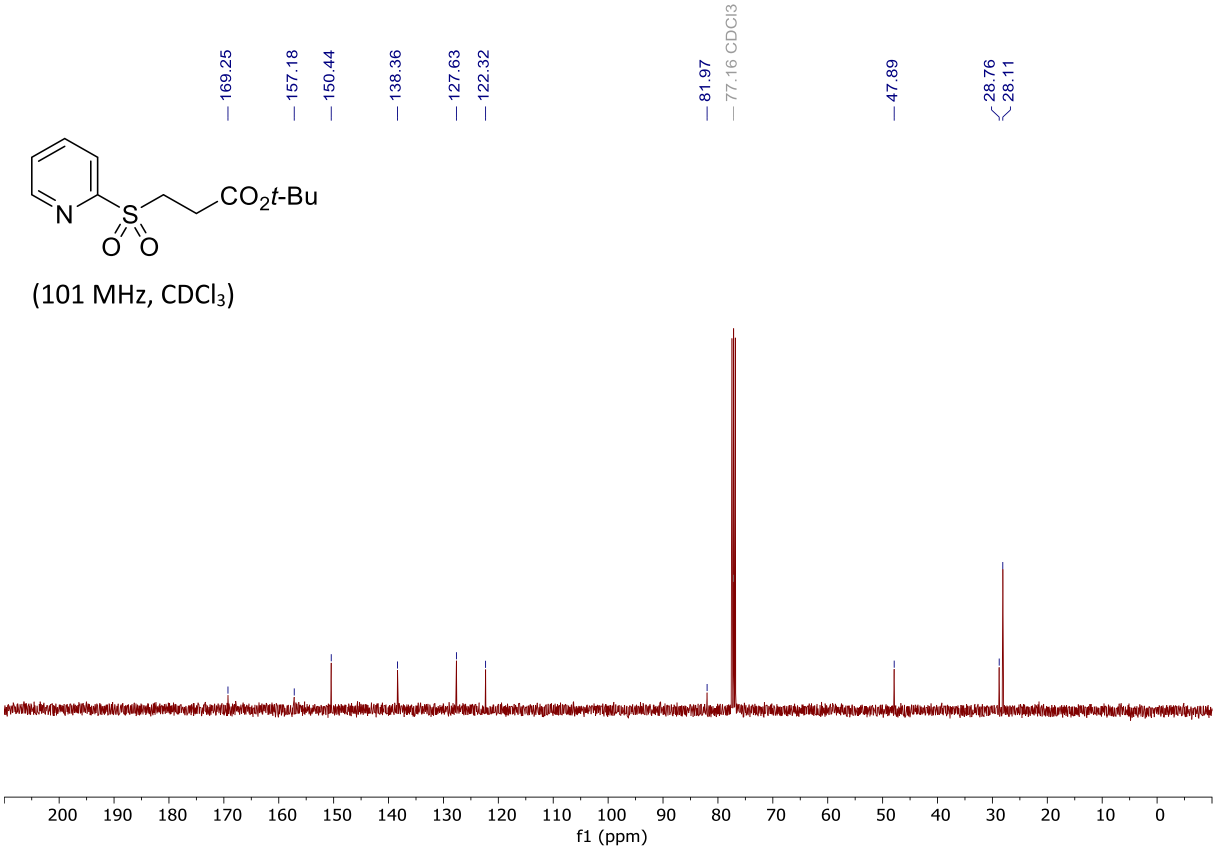

tert-Butyl 3-(quinolin-2-ylsulfonyl)propanoate (7b)

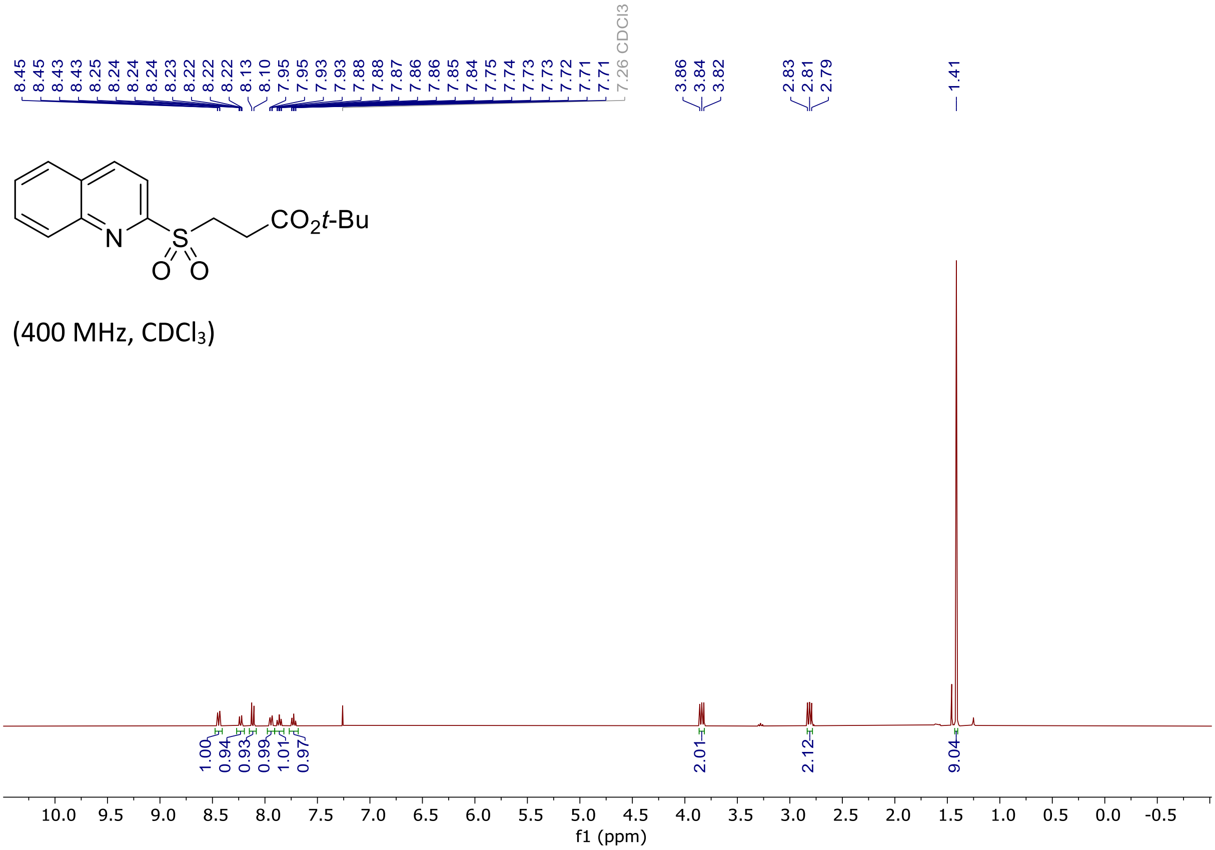

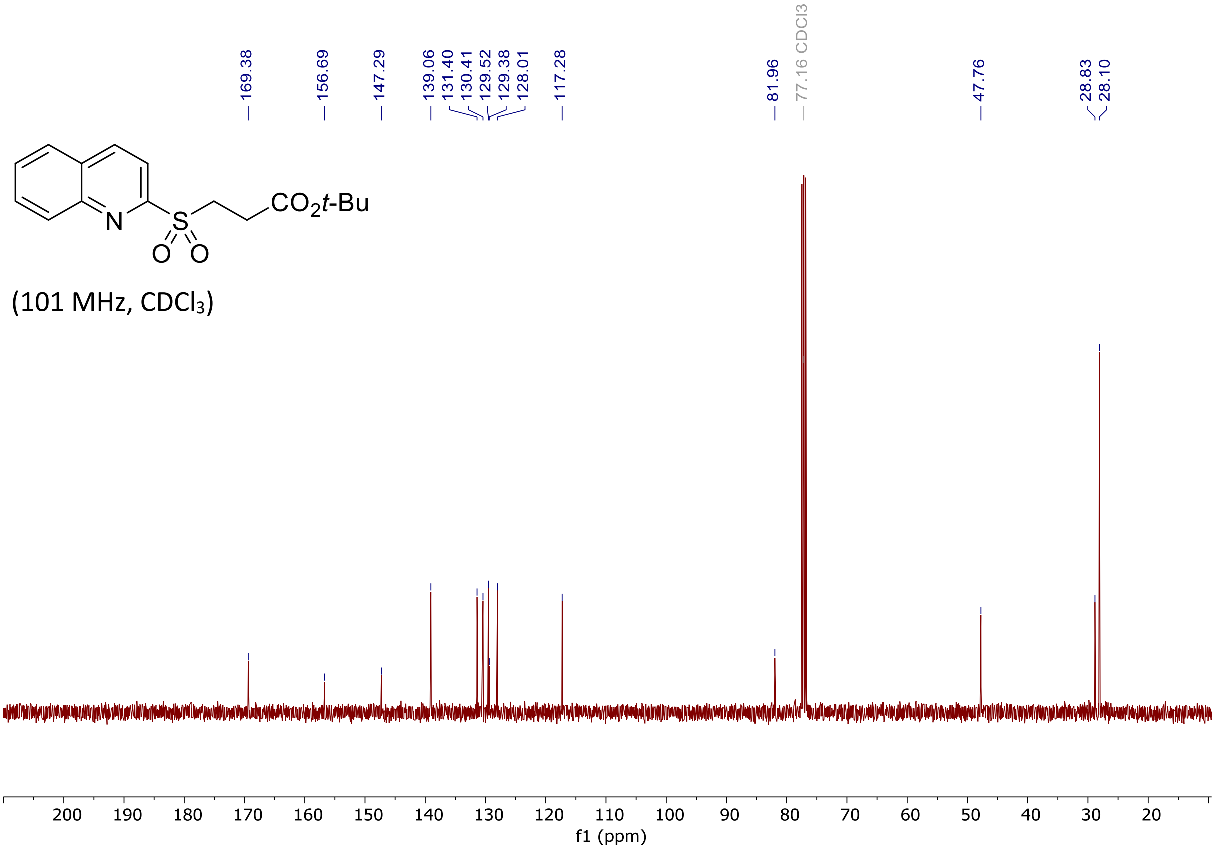

tert-Butyl 3-(phenylsulfonyl)propanoate (7c)

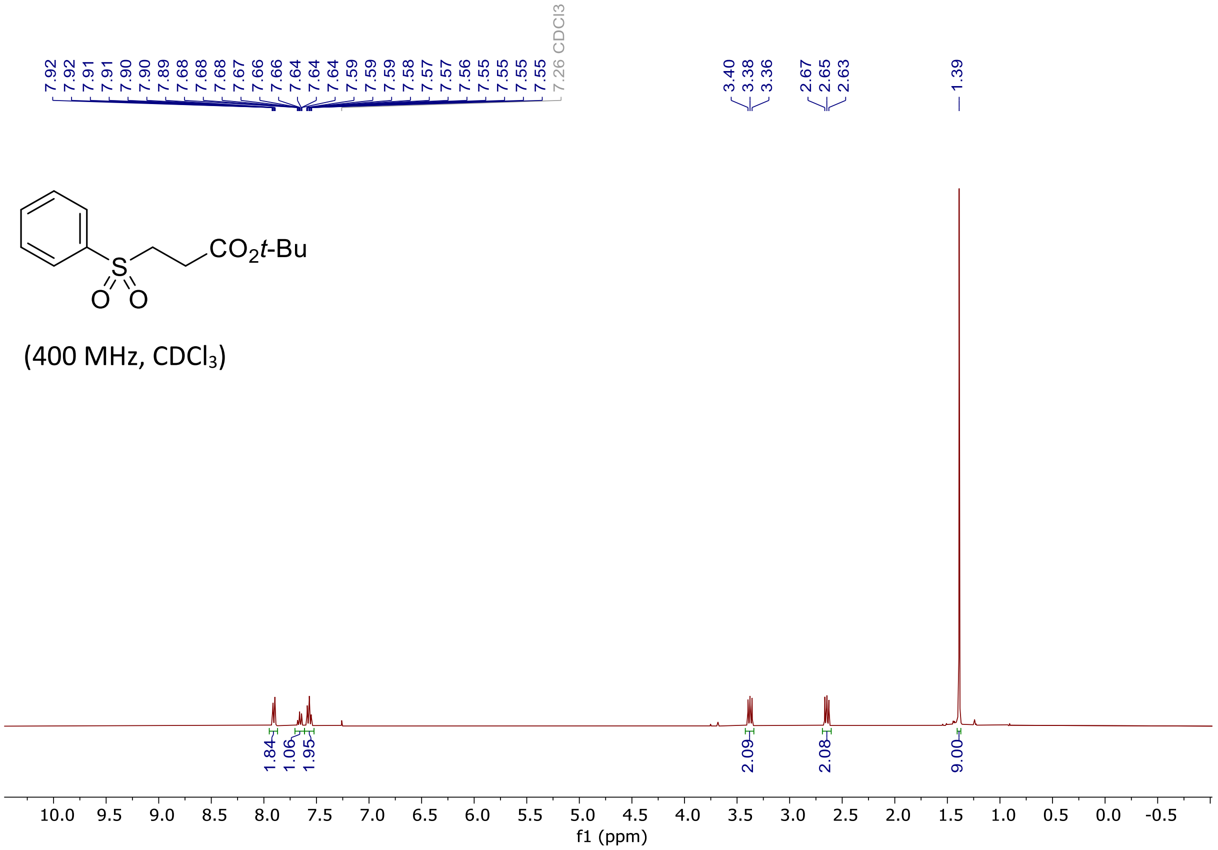

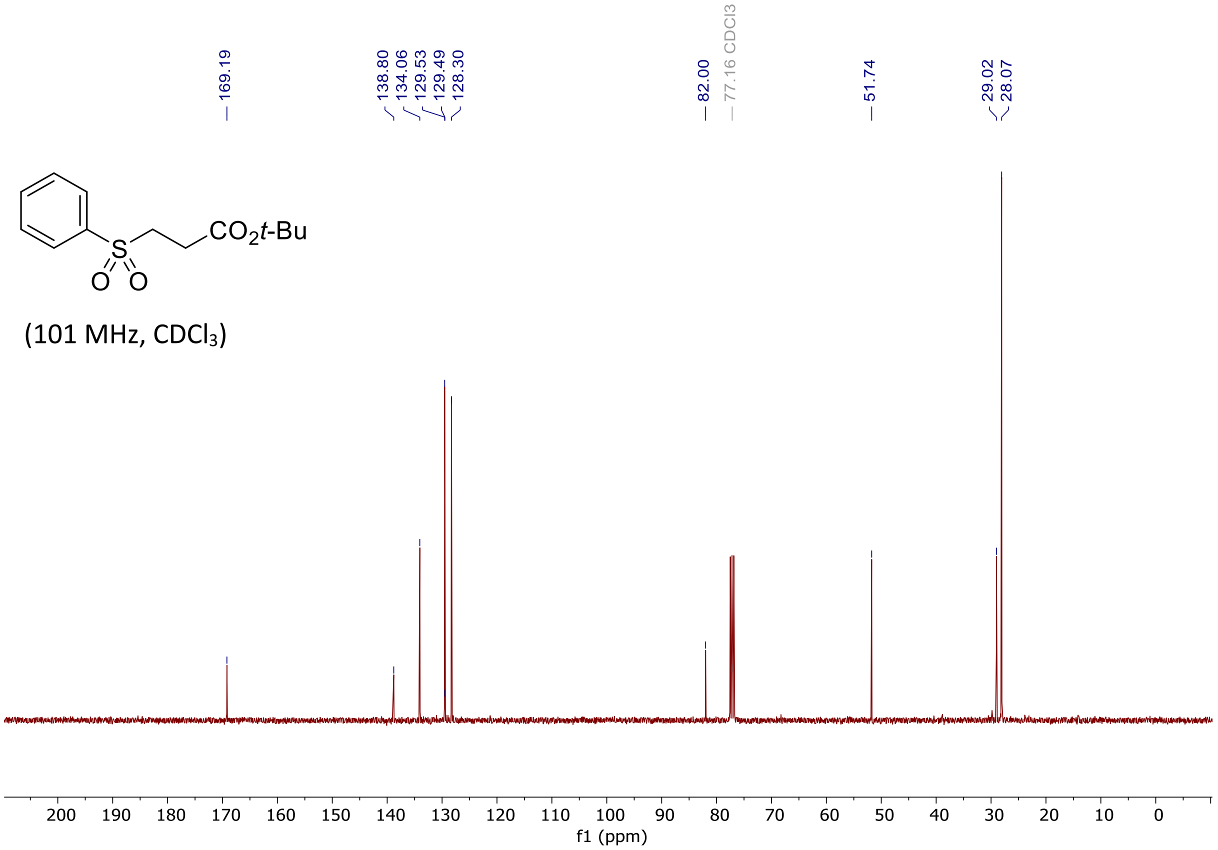

tert-Butyl 3-(naphthalen-2-ylsulfonyl)propanoate (7d)

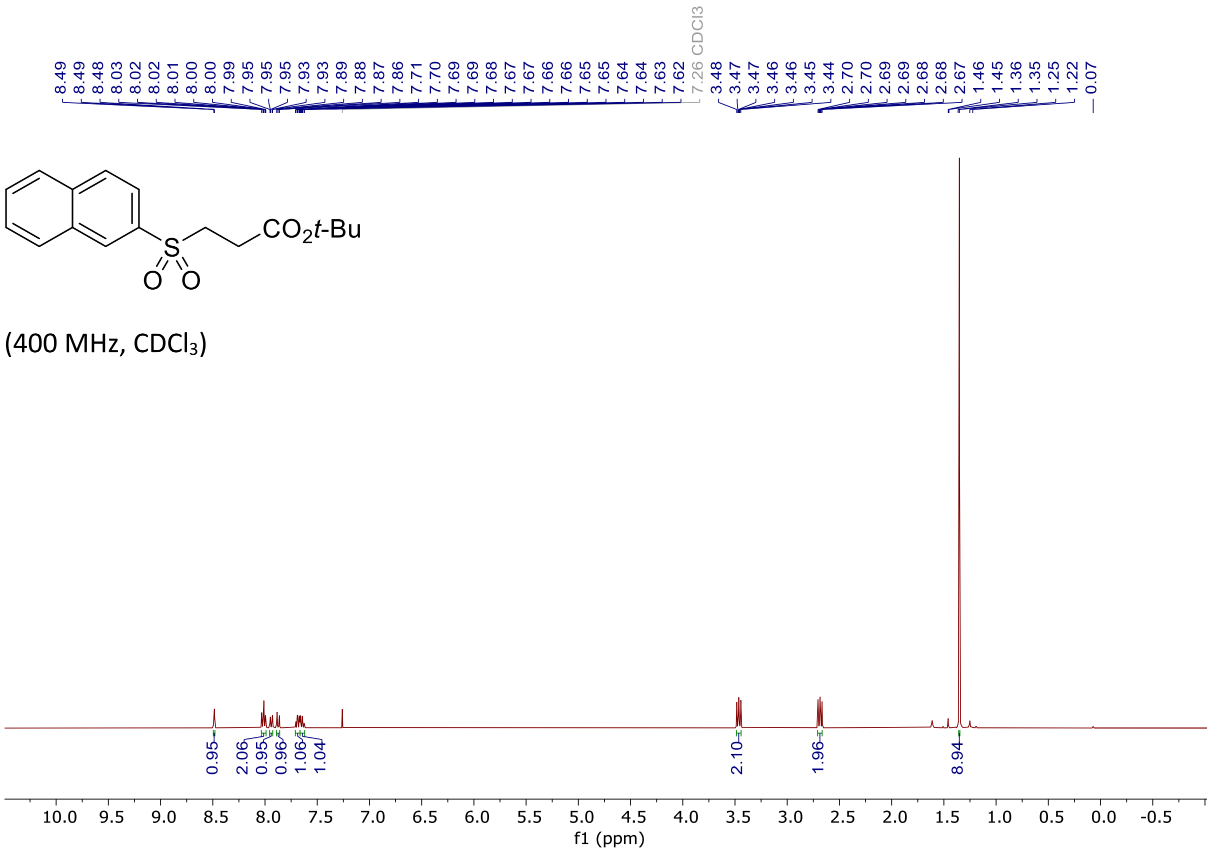

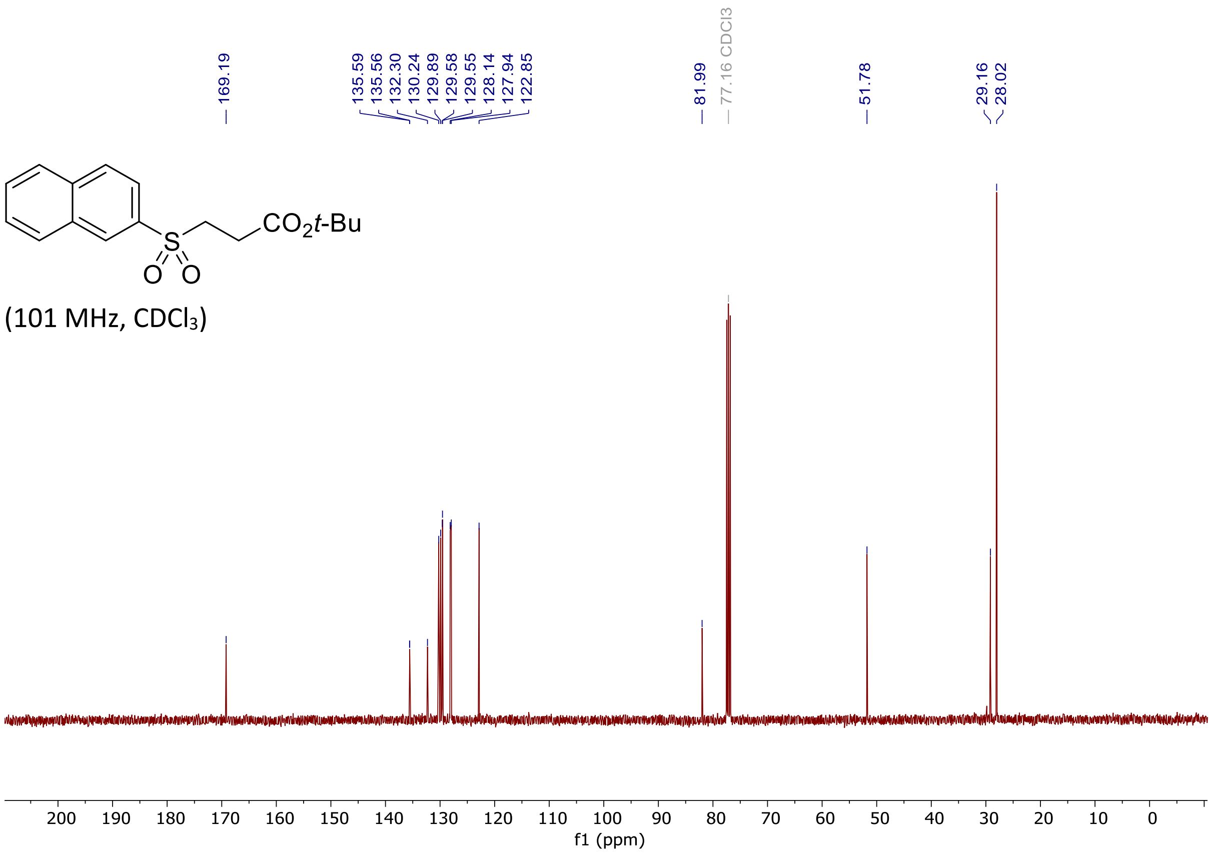

tert-Butyl 3-((3-chlorophenyl)sulfonyl)propanoate (7e)

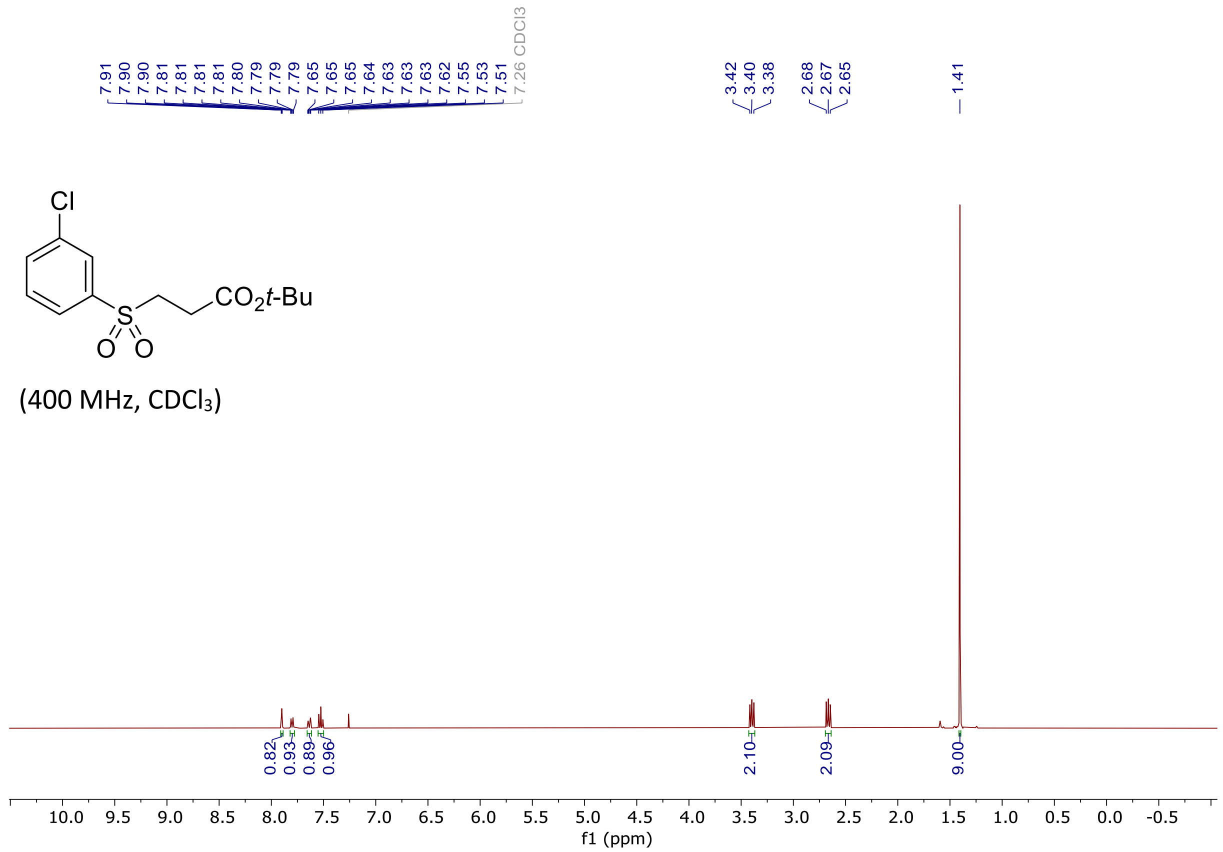

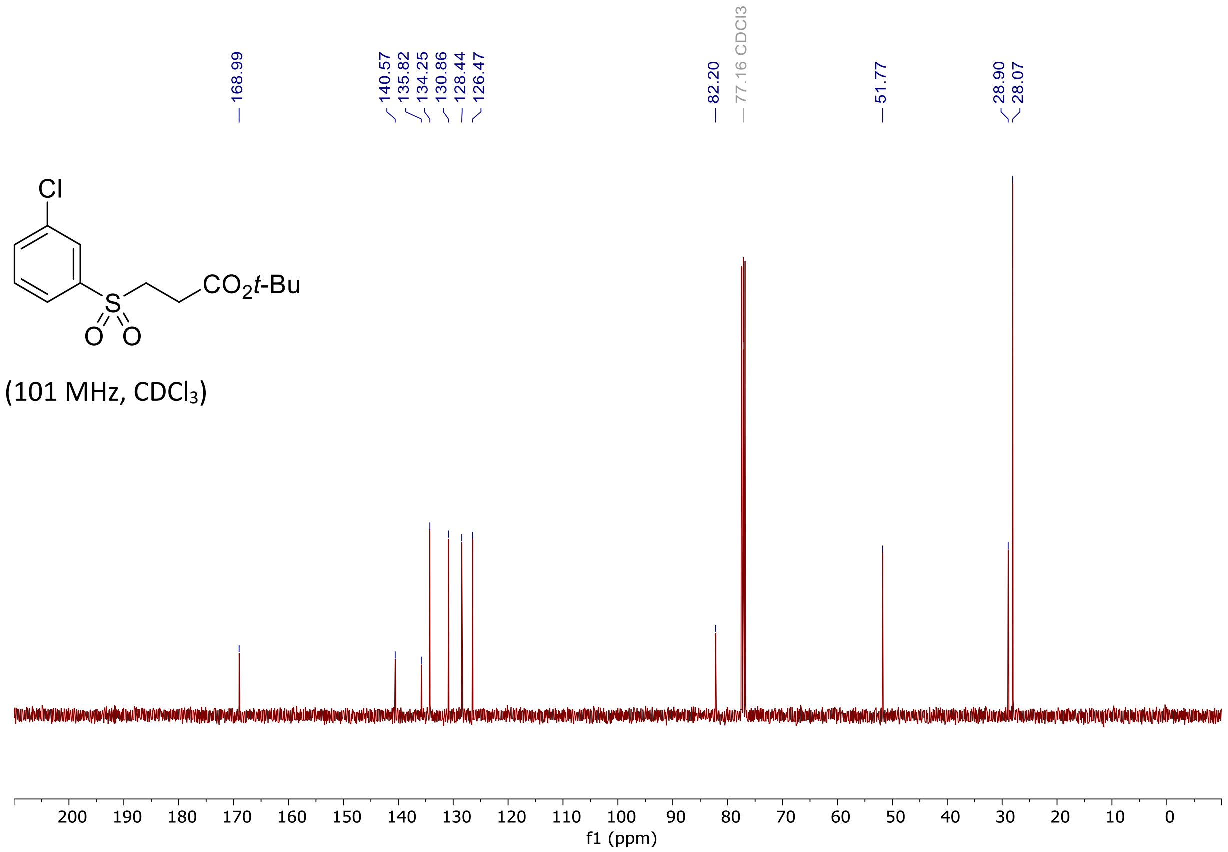

tert-Butyl 3-((3-(trimethylsilyl)phenyl)sulfonyl)propanoate (7f)

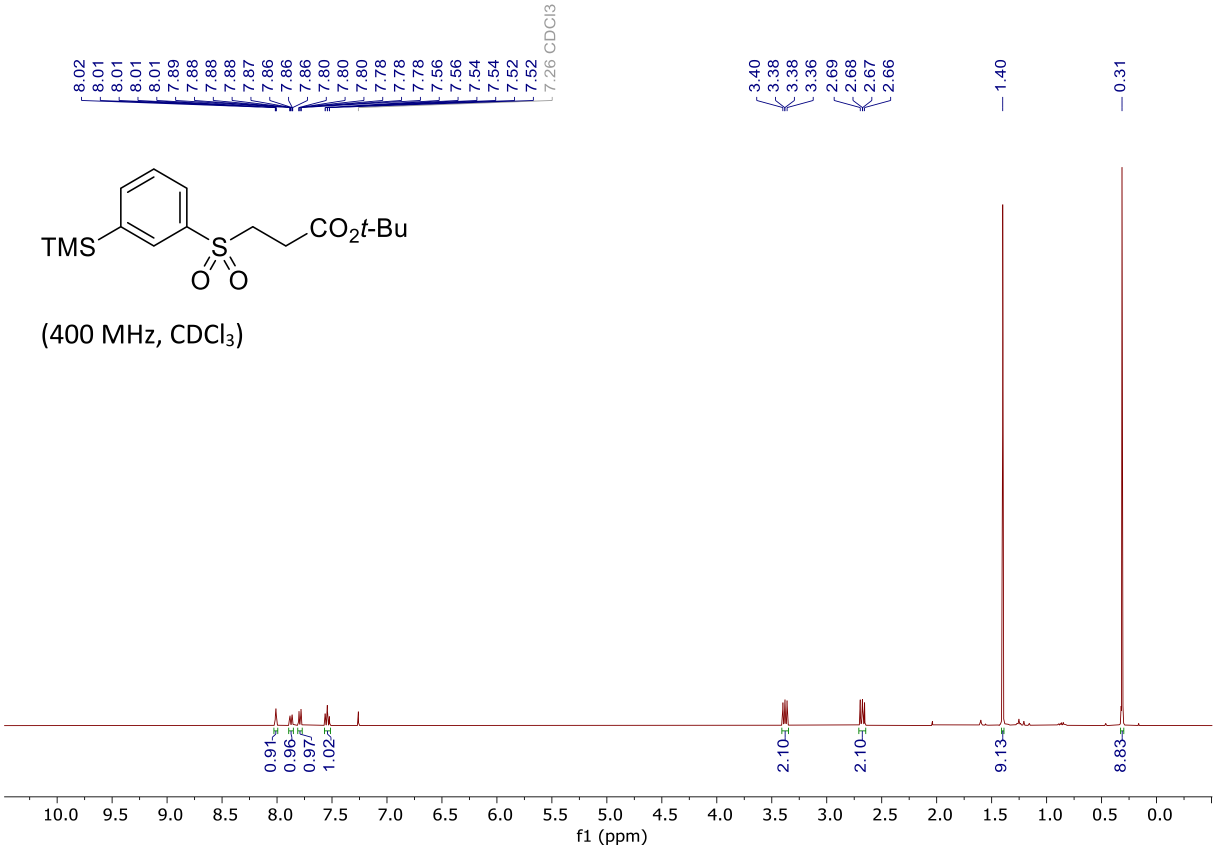

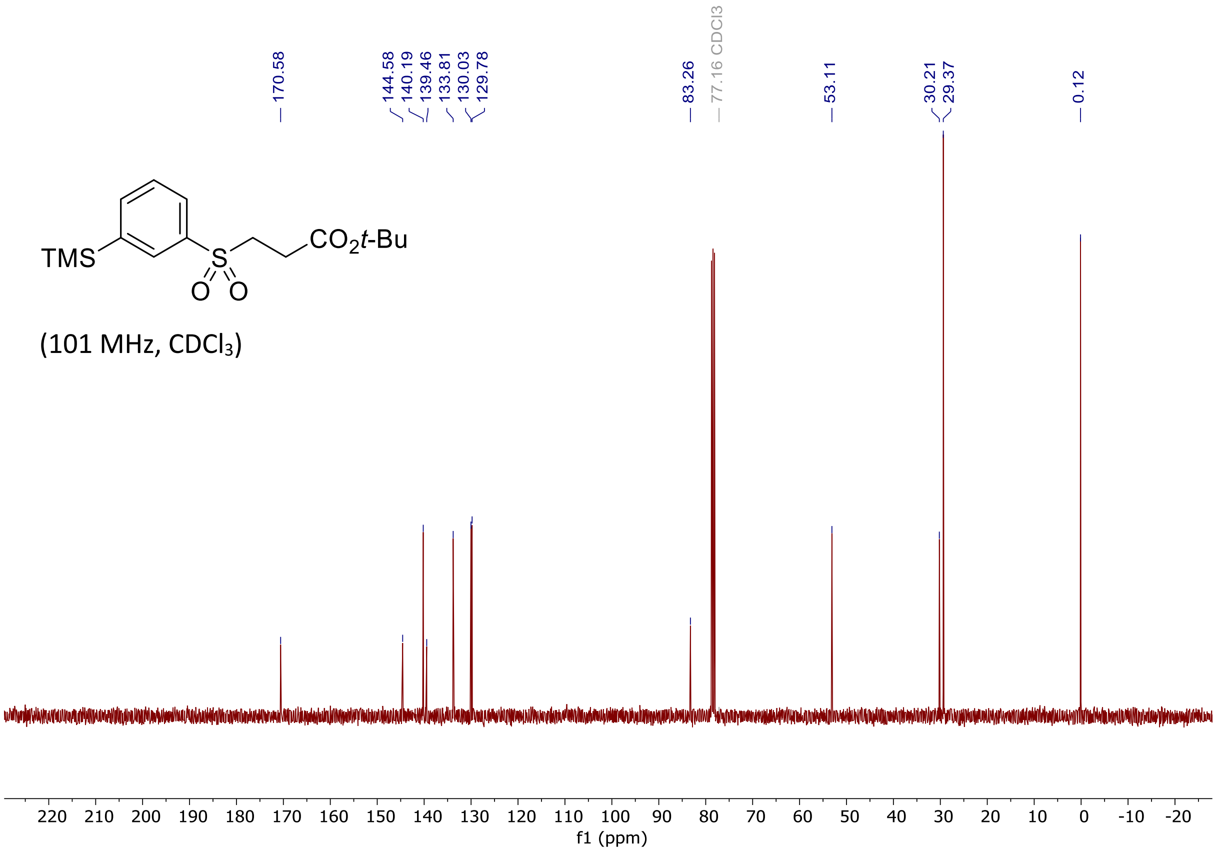

*t*ert-Butyl 3-((4-hydroxyphenyl)sulfonyl)propanoate (7g*)z

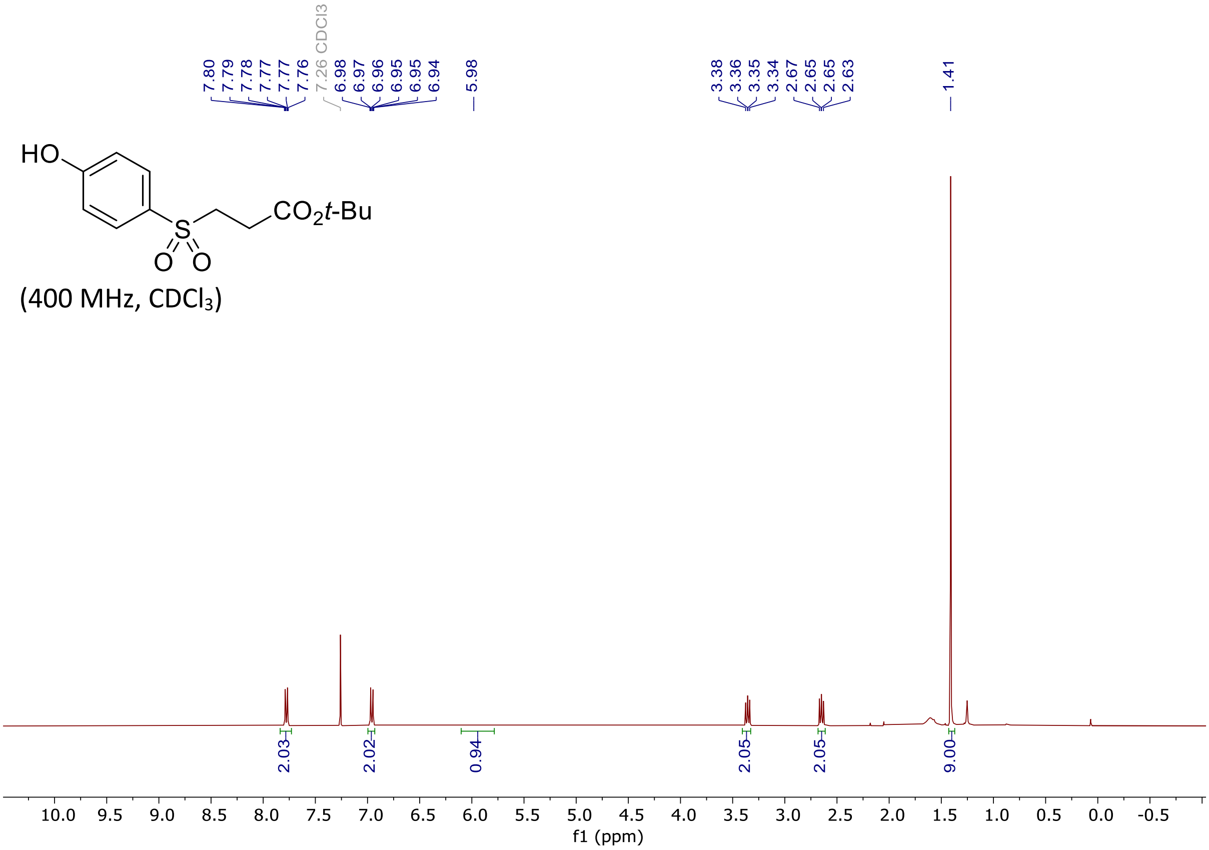

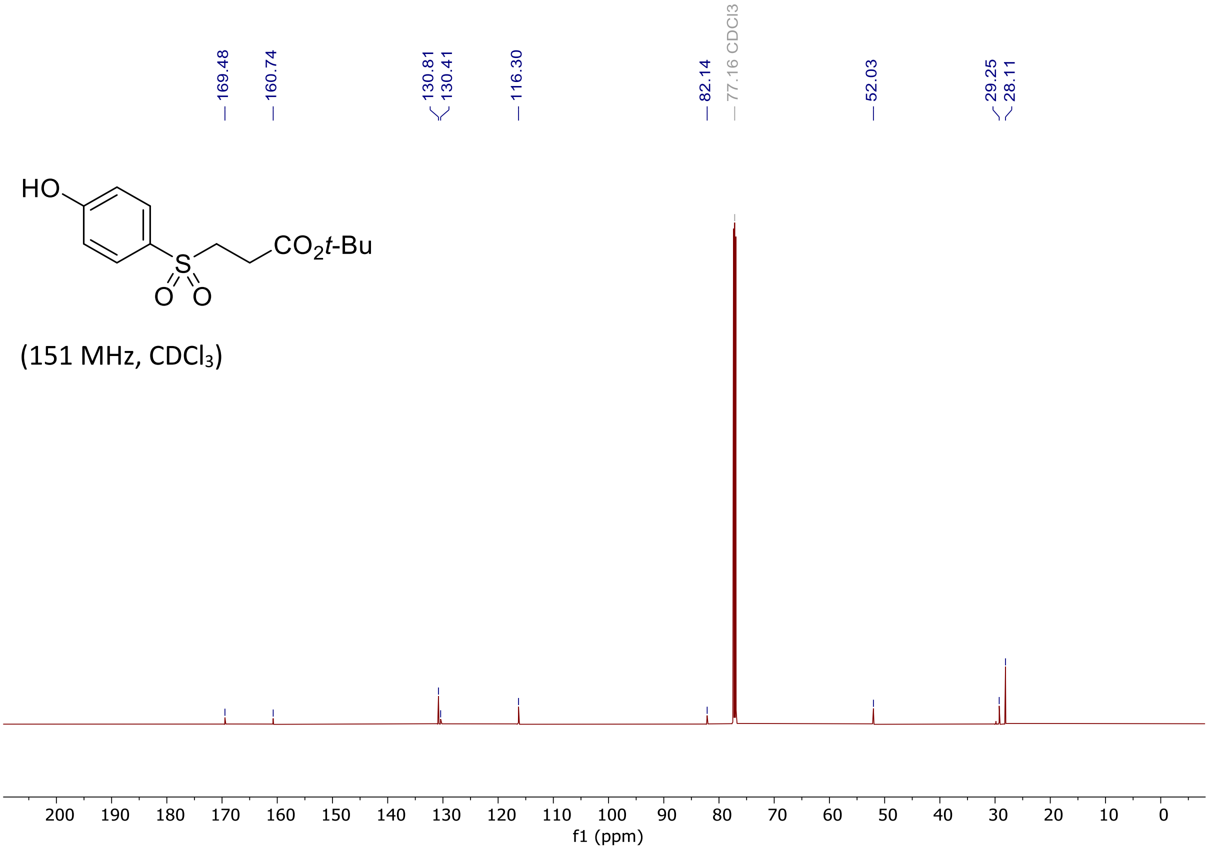

tert-Butyl 3-(thiophen-3-ylsulfonyl)propanoate (7h)

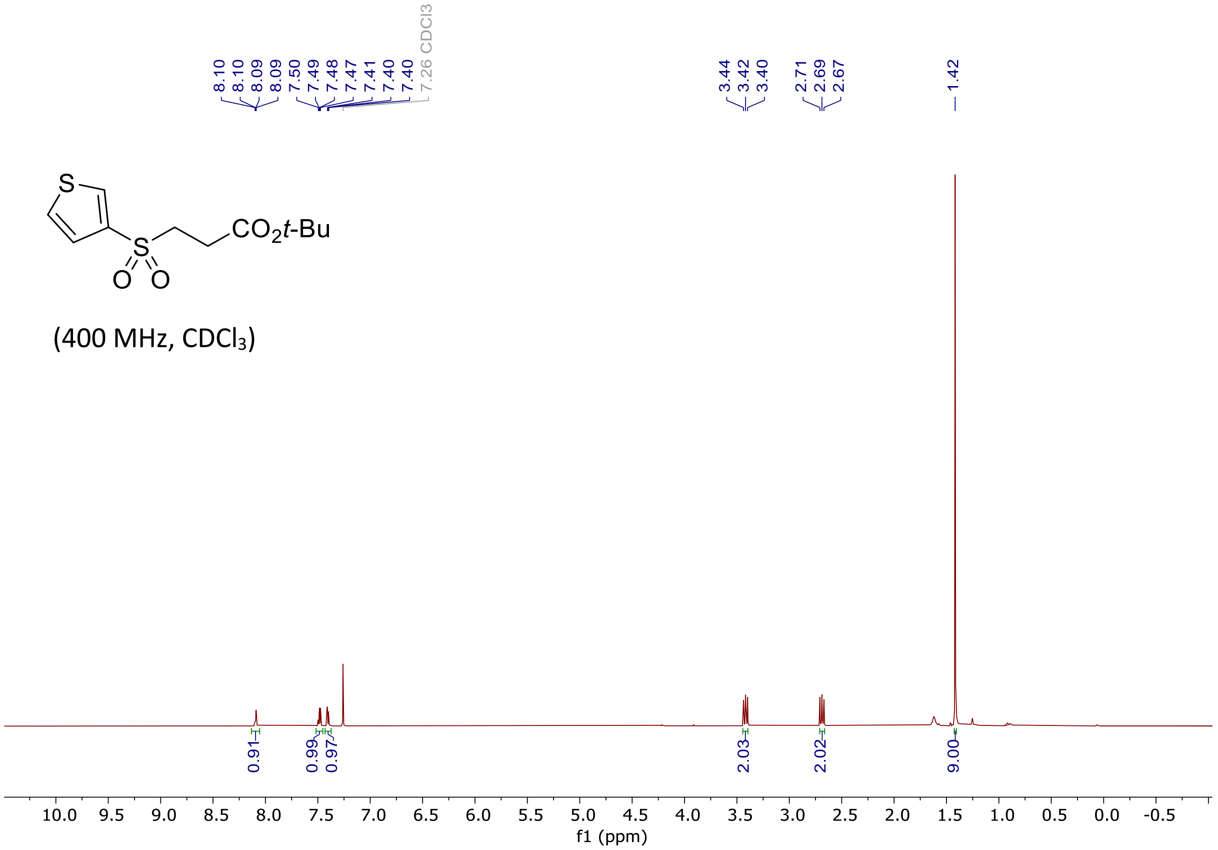

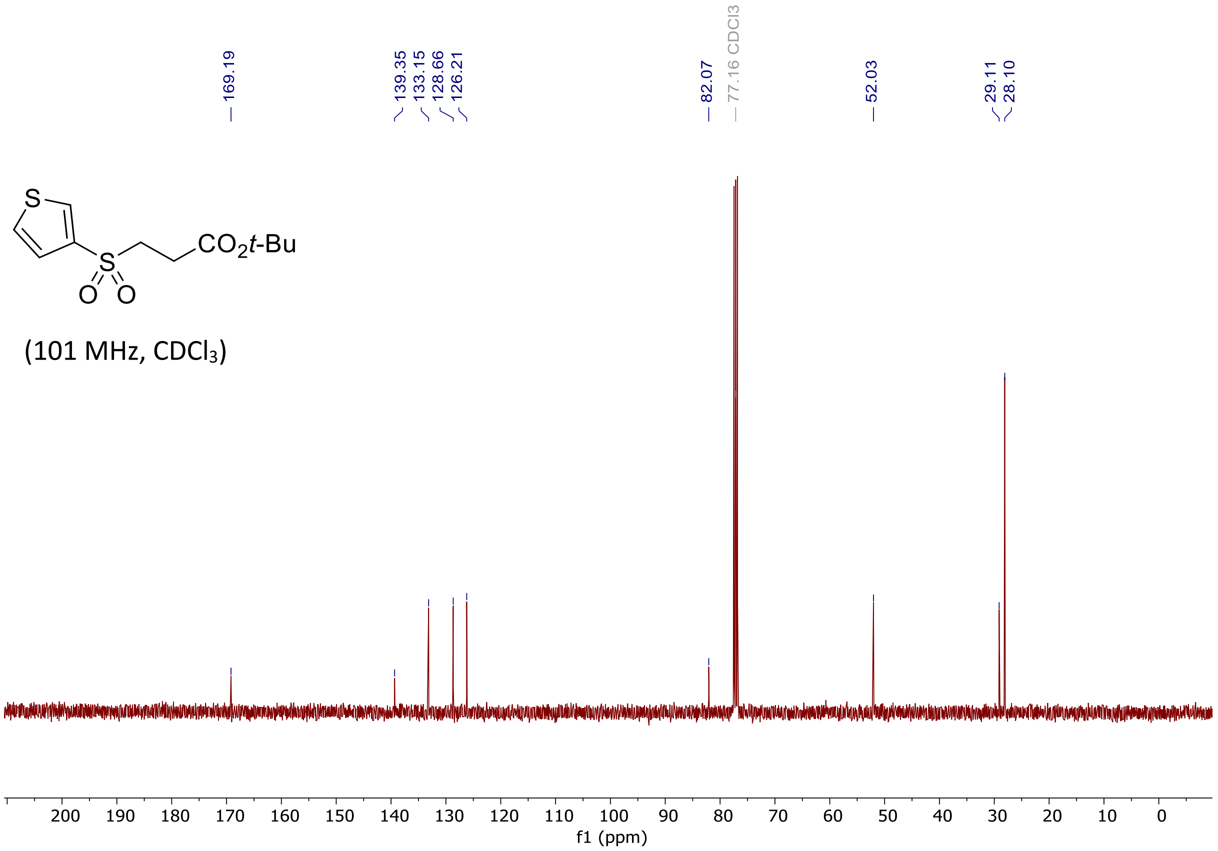

tert-Butyl 3-(furan-3-ylsulfonyl)propanoate (7i)

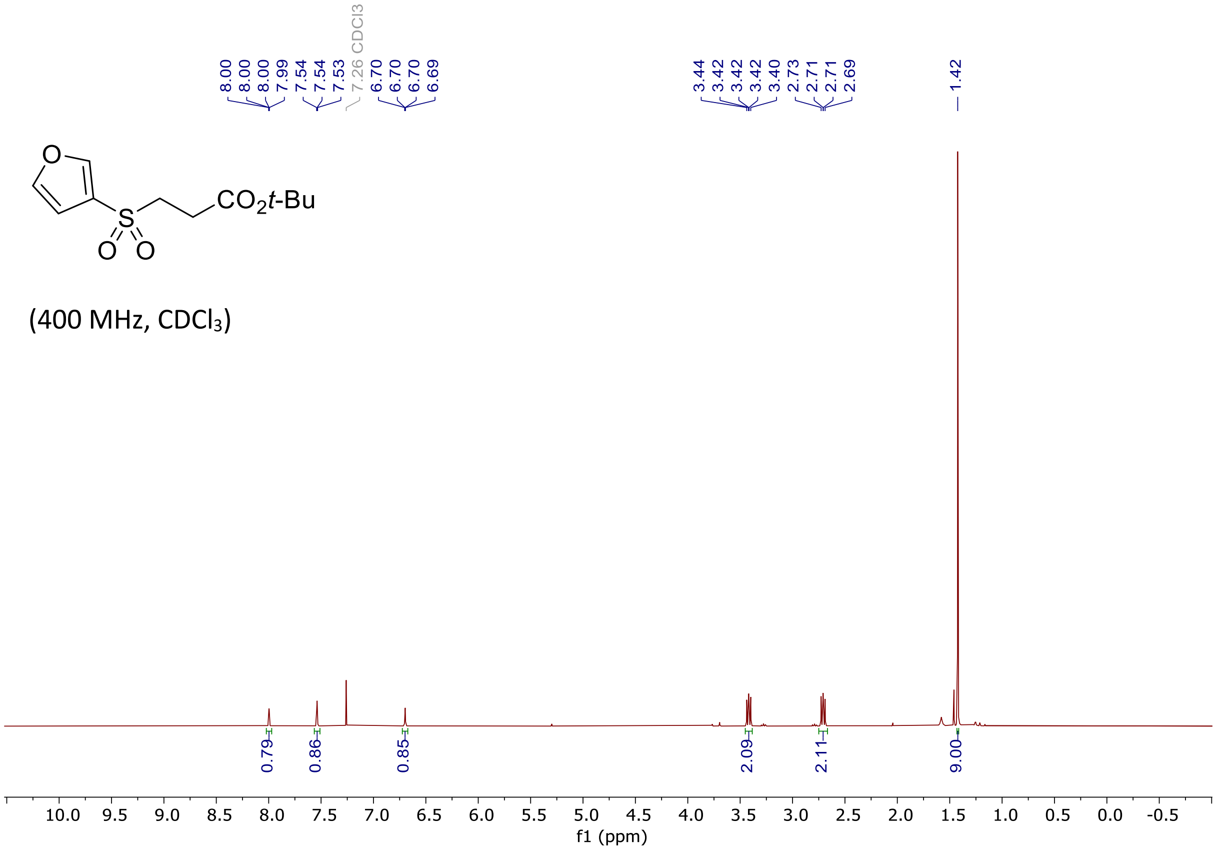

(400 MHz, $CDCl_3$)

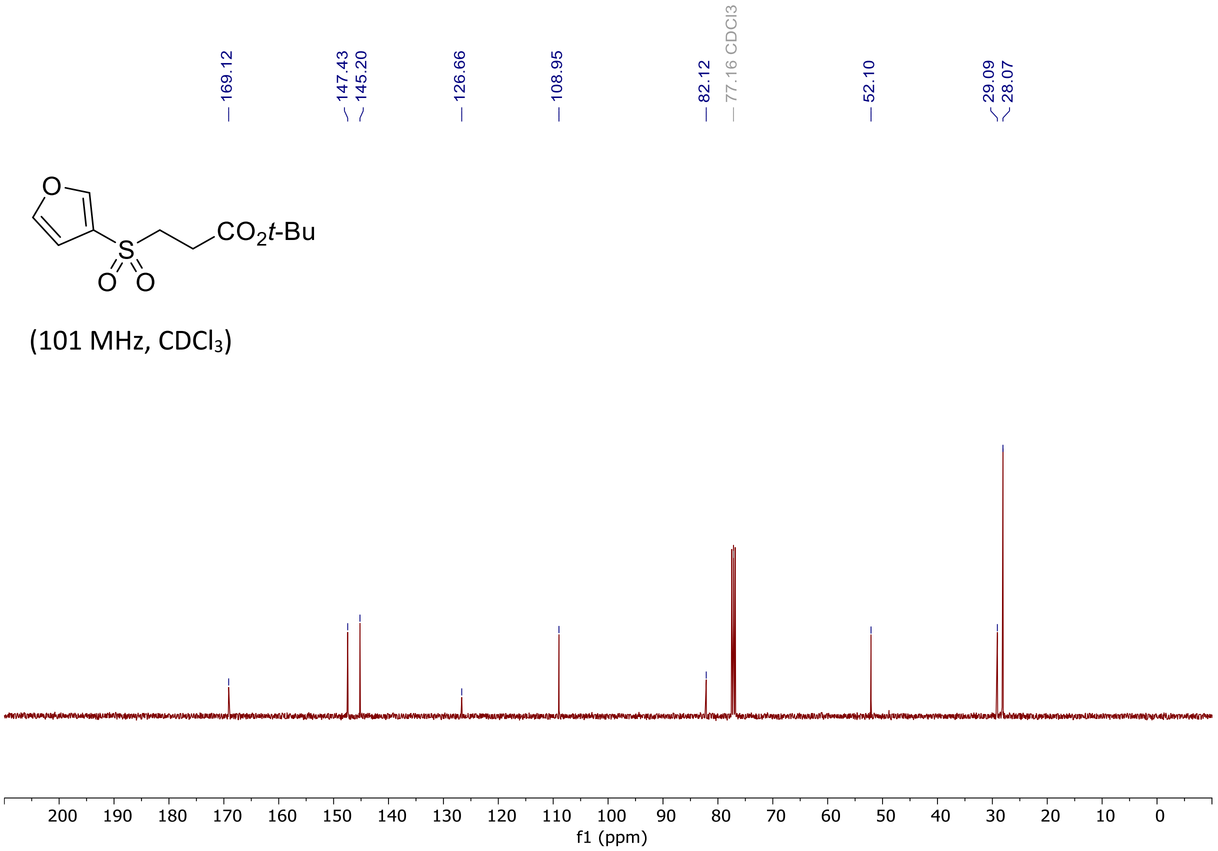

(101 MHz, $CDCl_3$)

tert-Butyl 2-(pyridin-2-ylsulfonyl)acetate (8a)

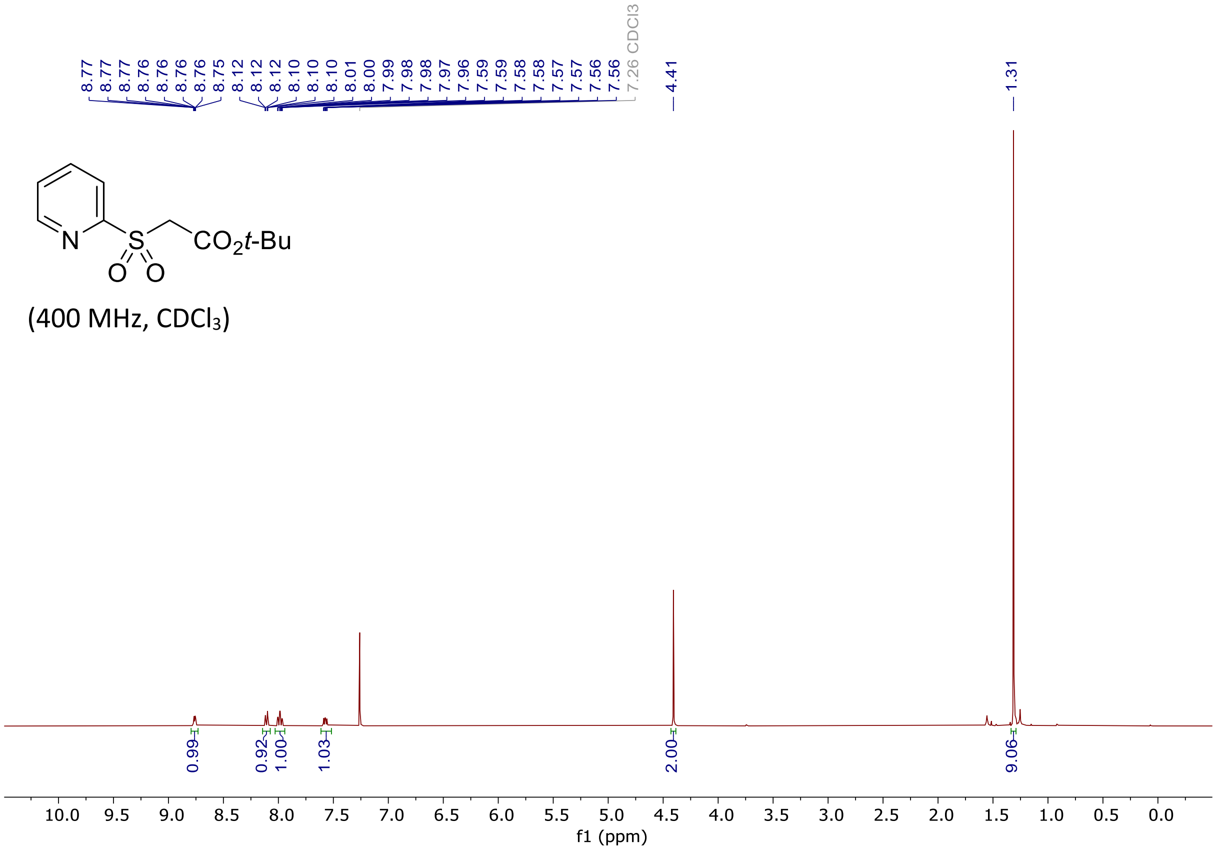

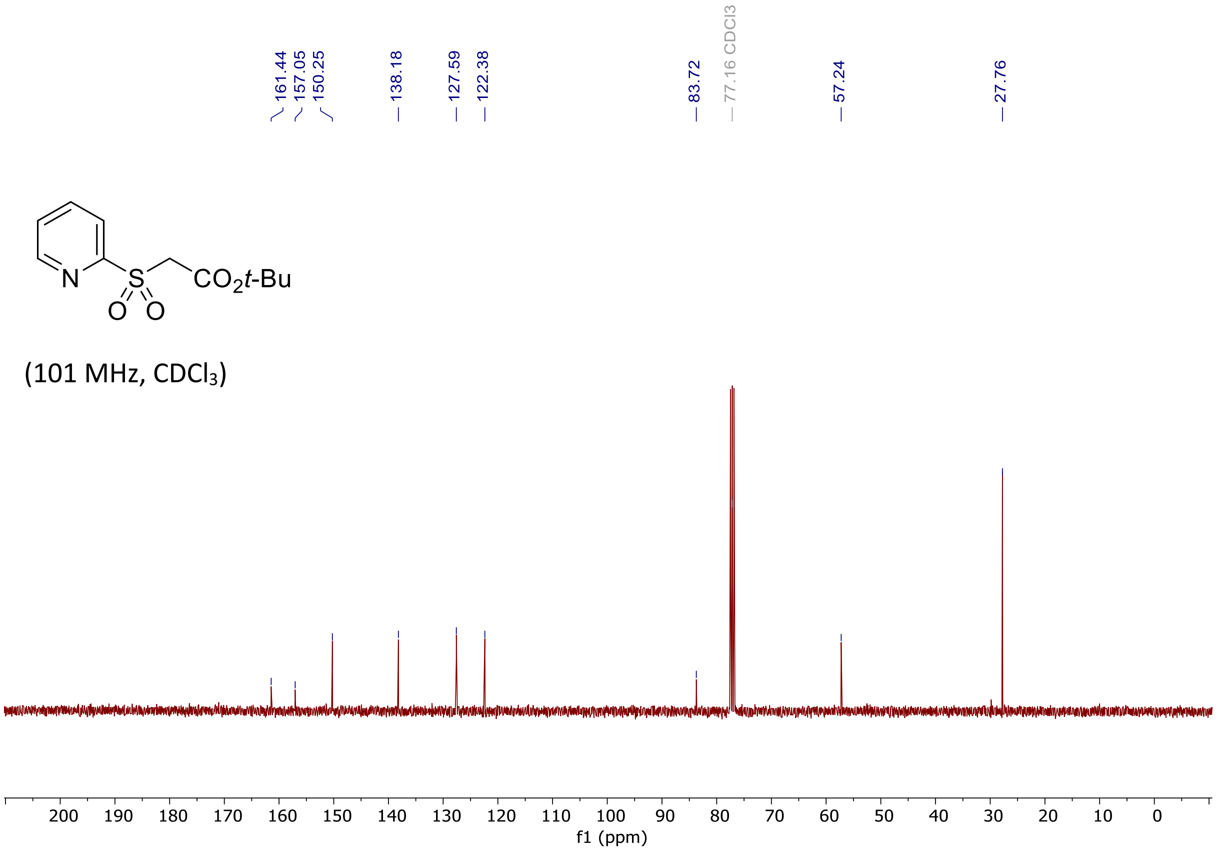

Pyridine-2-sulfonyl fluoride, PyFluor (8b)

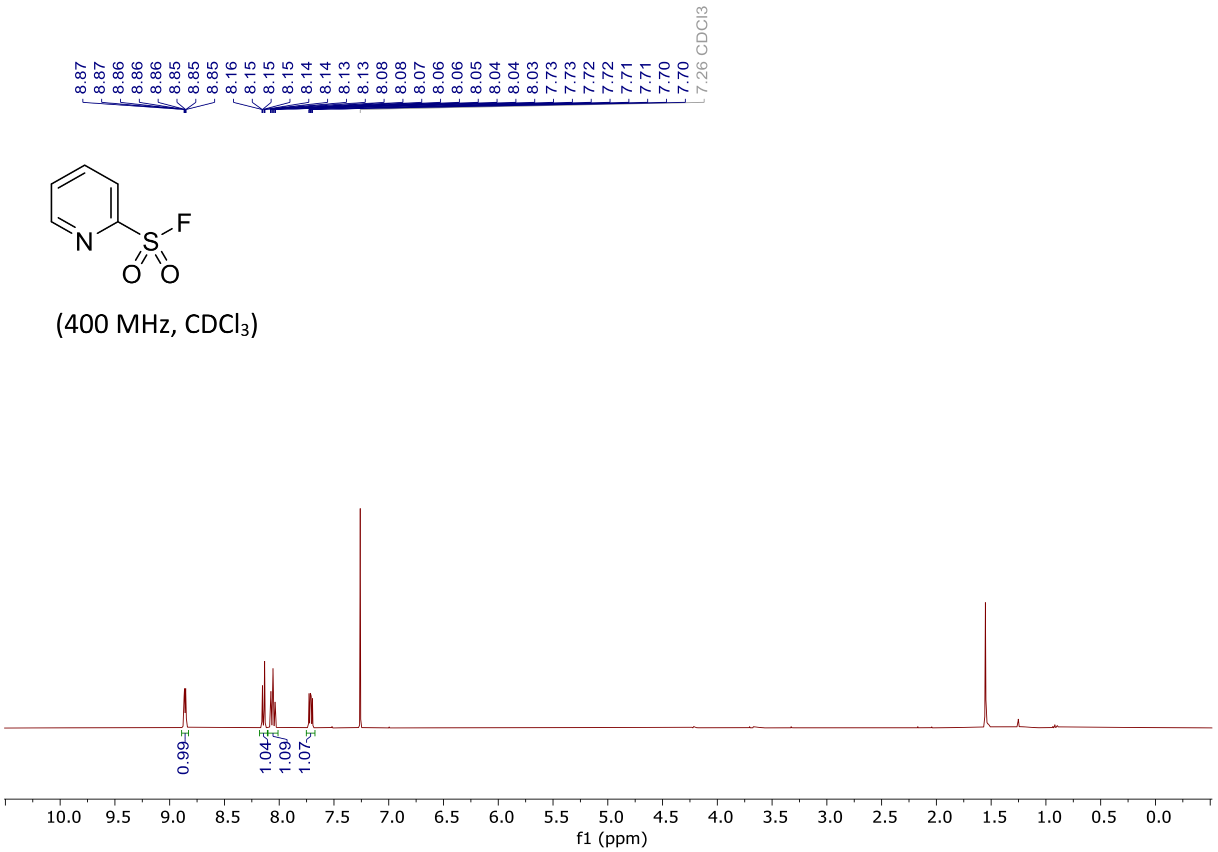

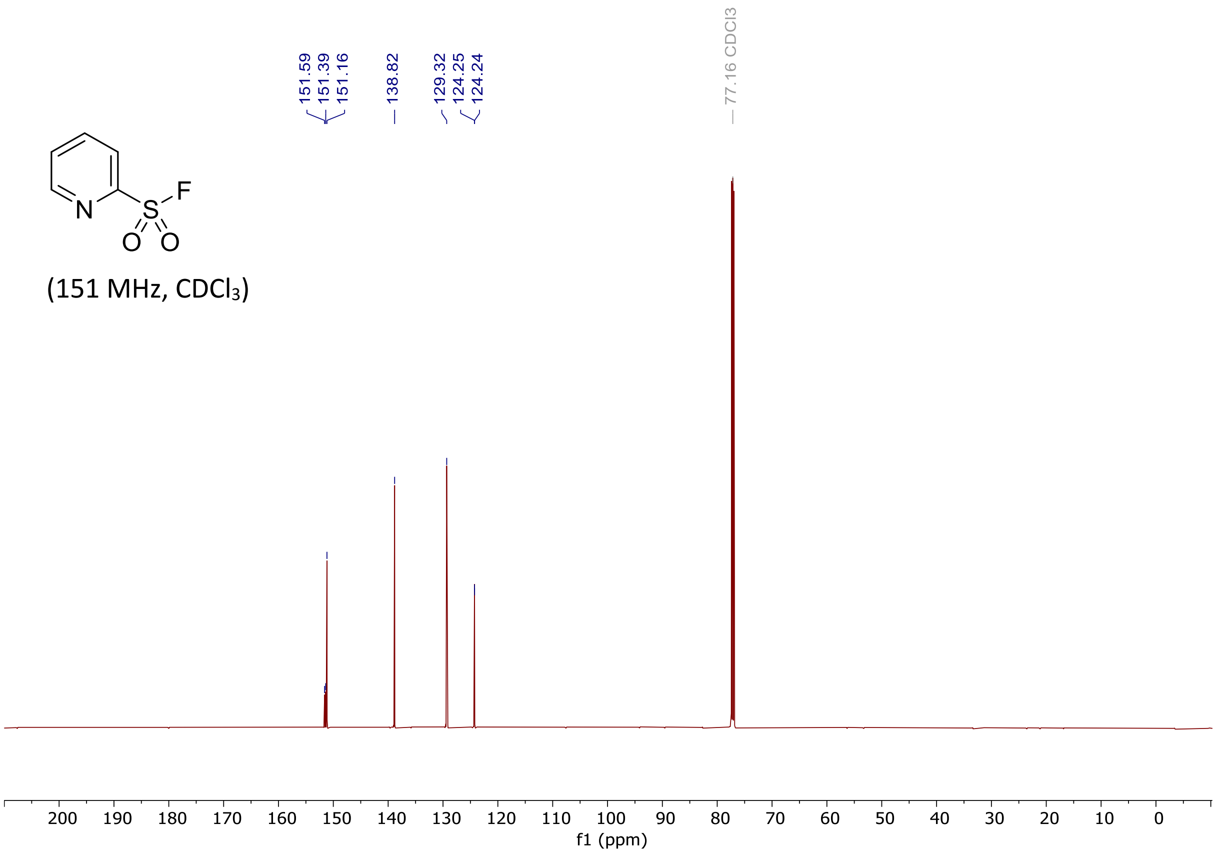

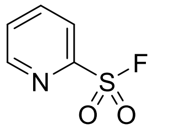

(377 MHz, $CDCl_3$)

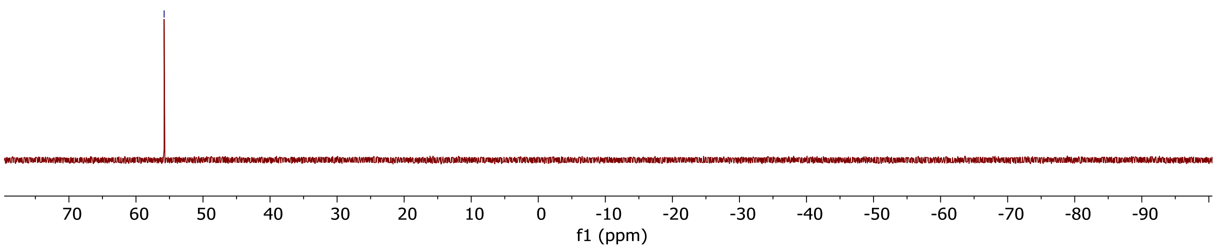

4-(Pyridin-2-ylsulfonyl)morpholine (8c)

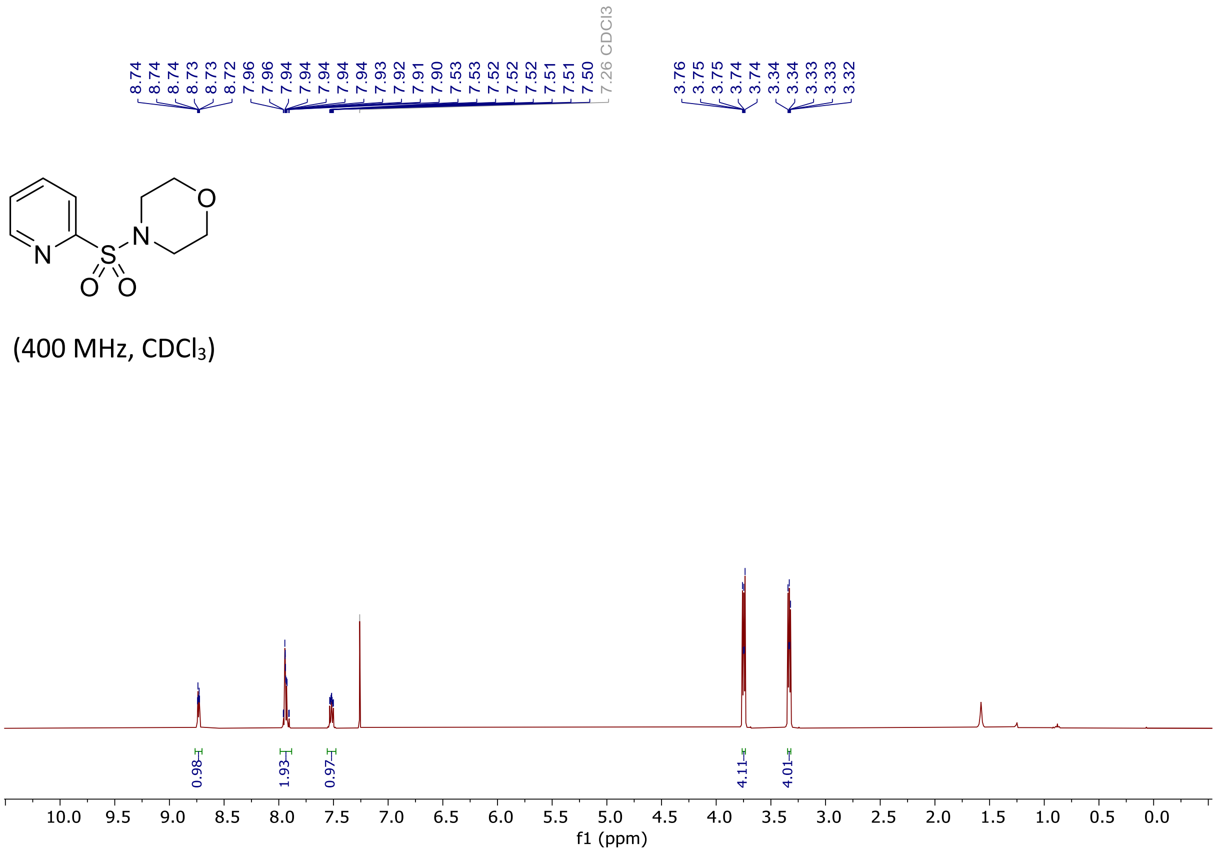

(400 MHz, $CDCl_3$)

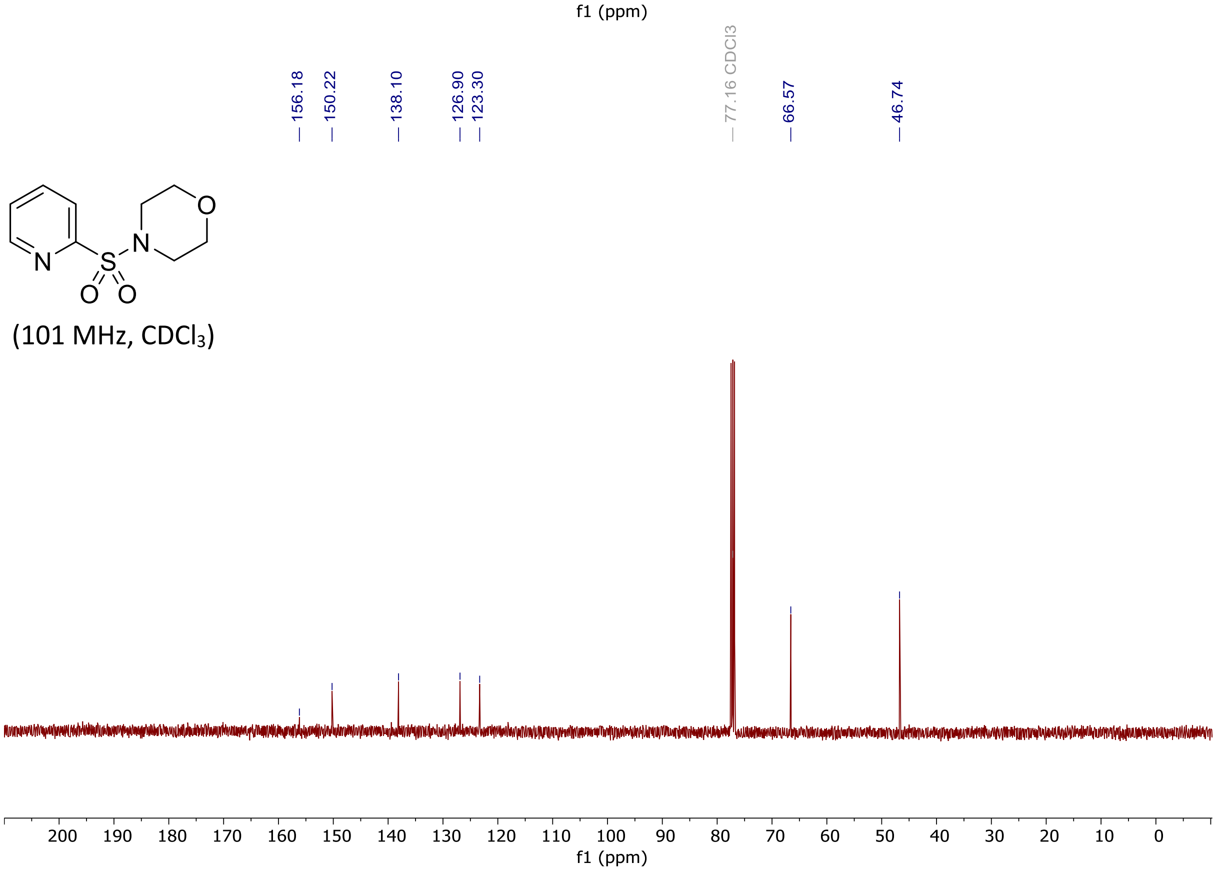

(101 MHz, $CDCl_3$)

7. References

(1) Bernhardson, D. J.; Widlicka, D. W.; Singer, R. A. Cu-Catalyzed Couplings of Heteroaryl Primary Amines and (Hetero)aryl Bromides with 6-Hydroxypicolinamide Ligands. *Org. Process Res. Dev.* **2019**, *23* (8), 1538-1551.
(2) Zhou, W.; Fan, M.; Yin, J.; Jiang, Y.; Ma, D. CuI/Oxalic Diamide Catalyzed Coupling Reaction of (Hetero)Aryl Chlorides and Amines. *J. Am. Chem. Soc.* **2015**, *137* (37), 11942-11945.
(3) Baskin, J. M.; Wang, Z. A mild, convenient synthesis of sulfinic acid salts and sulfonamides from alkyl and aryl halides. *Tetrahedron Lett.* **2002**, *43* (47), 8479-8483.
(4) Rule, N. G.; Detty, M. R.; Kaeding, J. E.; Sinicropi, J. A. Syntheses of 4H-Thiopyran-4-one 1,1-Dioxides as Precursors to Sulfone-Containing Analogs of Tetracyanoquinodimethane. *J. Org. Chem.* **1995**, *60* (6), 1665-1673.
(5) Cook, X. A. F.; Pantaine, L. R. E.; Blakemore, D. C.; Moses, I. B.; Sach, N. W.; Shavnya, A.; Willis, M. C. Base-Activated Latent Heteroaromatic Sulfinates as Nucleophilic Coupling Partners in Palladium-Catalyzed Cross-Coupling Reactions. *Angew. Chem.* **2021**, *60* (41), 22461-22468.
(6) Markovic, T.; Rocke, B. N.; Blakemore, D. C.; Mascitti, V.; Willis, M. C. Catalyst Selection Facilitates the Use of Heterocyclic Sulfinates as General Nucleophilic Coupling Partners in Palladium-Catalyzed Coupling Reactions. *Org. Lett.* **2017**, *19*, 6033-6035.
(7) Slutskyy, Y.; Overman, L. E. Generation of the Methoxycarbonyl Radical by Visible-Light Photoredox Catalysis and Its Conjugate Addition with Electron-Deficient Olefins. *Org. Lett.* **2016**, *18* (11), 2564-2567.
(8) De Gombert, A.; McKay, A. I.; Davis, C. J.; Wheelhouse, K. M.; Willis, M. C. Mechanistic Studies of the Palladium-Catalyzed Desulfinative Cross-Coupling of Aryl Bromides and (Hetero)Aryl Sulfinate Salts. *J. Am. Chem. Soc.* **2020**, *142* (7), 3564-3576.
(9) Higham, J. I.; Bull, J. A. Copper catalysed oxidative α-sulfonylation of branched aldehydes using the acid enhanced reactivity of manganese(iv) oxide. *Chem. Comm.* **2020**, *56* (33), 4587-4590.
(10) Bin, Y.; Hua, R. Synthesis of Alkyl Aryl Sulfones via Reaction of N-Arylsulfonyl Hydroxyamines with Electron-Deficient Alkenes. *Molecules* **2016**, *22* (1), 39.
(11) Rocke, B. N.; Bahnck, K. B.; Herr, M.; Lavergne, S.; Mascitti, V.; Perreault, C.; Polivkova, J.; Shavnya, A. Synthesis of Sulfones from Organozinc Reagents, DABSO, and Alkyl Halides. *Org. Lett.* **2014**, *16* (1), 154-157.
(12) Wang, L.; Cornella, J. A Unified Strategy for Arylsulfur(VI) Fluorides from Aryl Halides: Access to Ar-SOF3 Compounds. *Angew. Chem. Int. Ed.* **2020**, *59* (52), 23510-23515.
(13) Colombe, J. R.; DeBergh, J. R.; Buchwald, S. L. Synthesis of Heteroaryl Sulfonamides from Organozinc Reagents and 2,4,6-Trichlorophenyl Chlorosulfate. *Org. Lett.* **2015**, *17* (12), 3170-3173.
